# Cell shape regulates subcellular organelle location to control early Ca^2+^ signal dynamics in vascular smooth muscle cells

**DOI:** 10.1038/s41598-020-74700-x

**Published:** 2020-10-20

**Authors:** R. C. Calizo, M. K. Bell, A. Ron, M. Hu, S. Bhattacharya, N. J. Wong, W. G. M. Janssen, G. Perumal, P. Pederson, S. Scarlata, J. Hone, E. U. Azeloglu, P. Rangamani, R. Iyengar

**Affiliations:** 1 Department of Pharmacological Sciences, Institute for Systems Biomedicine, Icahn School of Medicine at Mount Sinai, One Gustave L. Levy Place, Box 1215, New York, NY 10029 USA; 2 Department of Mechanical Engineering, Columbia University, New York, NY 10027 USA; 3 Department of Chemistry and Biochemistry, Worcester Polytechnic Institute, Worcester, MA 01609 USA; 4 Department of Mechanical and Aerospace Engineering, University of California San Diego, La Jolla, CA 92093 USA; 5 Division of Nephrology, Department of Medicine, Icahn School of Medicine at Mount Sinai, New York, NY 10029 USA; 6 Carl Zeiss Microscopy LLC, White Plains, NY 10601 USA

**Keywords:** Cell signalling, Cellular signalling networks

## Abstract

The shape of the cell is connected to its function; however, we do not fully understand underlying mechanisms by which global shape regulates a cell’s functional capabilities. Using theory, experiments and simulation, we investigated how physiologically relevant cell shape changes affect subcellular organization, and consequently intracellular signaling, to control information flow needed for phenotypic function. Vascular smooth muscle cells going from a proliferative and motile circular shape to a contractile fusiform shape show changes in the location of the sarcoplasmic reticulum, inter-organelle distances, and differential distribution of receptors in the plasma membrane. These factors together lead to the modulation of signals transduced by the M_3_ muscarinic receptor/G_q_/PLCβ pathway at the plasma membrane, amplifying Ca^2+^ dynamics in the cytoplasm, and the nucleus resulting in phenotypic changes, as determined by increased activity of myosin light chain kinase in the cytoplasm and enhanced nuclear localization of the transcription factor NFAT. Taken together, our observations show a systems level phenomenon whereby global cell shape affects subcellular organization to modulate signaling that enables phenotypic changes.

## Introduction

Cells utilize receptors on the plasma membrane to transduce a range of extracellular signals to regulate function in the cytoplasm and the nucleus^1^. Reaction kinetics of the biochemical interactions that comprise the signaling networks regulate the temporal dynamics of activation and inactivation of signaling components and effectors^2^. However, information flow within cells is not just temporally regulated, it is also spatially regulated by the shape of the cell^3–5^ and surface-to-volume ratio at the plasma membrane^6,7^. An additional layer of complexity is conferred by the spatial transfer of information by signaling reactions that occur within or at intracellular organelles. Recently, studies have shown that signaling at the endosomes plays an important role in prolonging cAMP dynamics through GPCRs^8^ and in EGFR dynamics^9^. An important compartmental regulation of organelle-based signaling is Ca^2^^+^ dynamics, since endoplasmic/sarcoplasmic reticulum is a regulatable Ca^2+^ store in cells. Ca^2+^ is a ubiquitous signaling molecule that controls many cellular functions including fertilization, proliferation, migration and cell death^10–13^. Ca^2+^ is able to participate in controlling this diverse array of functions due to the precise control of Ca^2+^ concentration within the cell. In vascular smooth muscle cells (VSMC), Ca^2+^ regulates both contractility and gene expression through IP_3_-mediated Ca^2+^ release by IP_3_R receptors located on the membrane of the sarcoplasmic reticulum (SR) and through Ca^2+^ influx at the plasma membrane^14–16^. Ca^2+^-calmodulin activates myosin light chain kinase (MLCK), which phosphorylates the light chain of myosin, initiating contraction^17^. Ca^2+^ also activates protein kinases and phosphatases that regulate transcription regulators that define the phenotypic status of VSMC^18^. Ca^2+^ activates calcineurin, which dephosphorylates the transcription factor nuclear factor of activated T-cells (NFAT) in the cytoplasm, resulting in its nuclear accumulation and expression of NFAT-regulated genes^19^. Ca^2+^ also activates calmodulin kinase II (CaMKII), that phosphorylates the transcription factor serum response factor (SRF)^20^ which, as a complex with myocardin, controls the expression of proteins necessary for the contractile function of VSMC^21^.

VSMC in the medial layer of the walls of blood vessels are not terminally differentiated and can undergo phenotypic transitions during injury and disease states^22–24^. VSMC shape and function are closely related; increasing elongation or aspect ratio (AR, defined as the ratio of the short axis to long axis) is correlated with differentiation and contractility^25,26^. This fusiform shape represents the normal physiological state of contractile VSMCs^27^. Previous studies have shown that changes in cell shape are associated with major changes in subcellular organization, which may affect cell physiology and phenotype including contractility, proliferation, and differentiation states^28–30^. Recent experiments have shown that local microdomains Ca^2+^ are possible because of hindered diffusion of IP_3_^31^ and differential subcellular distribution of IP_3_R^32^. However, the mechanisms underlying the relationship between global cell shape and changes in cell physiology such as contractility, remain poorly understood.

Based on the observations that cell shape and Ca^2+^ signaling closely regulate the contractile phenotype of differentiated VSMCs, we hypothesized that cell shape regulates organelle location, including the relative distances between plasma membrane, endoplasmic/sarcoplasmic reticulum (ER/SR) and the nucleus, to modulate cellular functions. We sought to answer the following question—does cell-shape-dependent organelle localization drive spatial control of information flow and affect cellular function? The answer to this question is important for understanding how mechano-chemical relationships control cell shape, signaling, and phenotype over multiple length scales. We used 3D biochips to culture VSMCs in different shapes and found that different cell shapes have different PM–SR (plasma membrane—sarcoplasmic reticulum) distances. We then developed theoretical and computational models to represent the spatio-temporal dynamics of IP_3_/Ca^2+^ transients mediated by Muscarinic Receptor 3 (M_3_R)/Phospholipase Cβ (PLCβ) pathway. Activation of M_3_R mediates contractility in VSMC by activating PLCβ resulting in phosphoinositide hydrolysis and IP_3_ mediated Ca^2+^ release from the SR^33,34^. We tested the effect of cell shape on VSMC contractility and found an unexpected modulation of organelle location as a function of cell shape and that this change in organelle location results in signal amplification in the cytoplasm and nucleus.

## Methods

### Cell culture

A10 cells, which are VSMCs from thoracic/medial layer of rat aortas, were obtained from American Type Culture Collection (CRL-1476). A10 cells were maintained in Dulbecco’s modified eagle’s medium (DMEM, Gibco), supplemented with 10% Fetal Bovine Serum, 1% penicillin/streptomycin, at 37 °C and 5% CO_2_. Cells were transfected using Neon Transfection System (Life Technologies) according to manufacturer's instructions. Briefly, 5 × 10^5^ cells were electroporated with 1 µg DNA in suspension buffer, with the following electroporation settings: 1400 V, 2 pulses, 20 ms pulses each. Cells were then suspended in DMEM supplemented with 10% FBS and then allowed to adhere on 3D biochips. Forty-eight hours post-transfection, cells were imaged using Hanks Balanced Salt Solution (HBSS) supplemented with CaCl_2,_ MgCl_2_ and 10 mM HEPES.

### Fabrication of 3D biochips

Patterned surfaces were fabricated by conventional photolithography using SU8 photoresist^35^. Complete details are provided in^36^ (see Fig. 5 in this reference for details on the ellipsoid shape). Briefly, cover glass slides were cleaned by sonication in isopropanol and deionized water for 15 min and baked at 110 °C overnight and photolithography was subsequently performed using standard vacuum hard-contact mode. Before plating of cells onto 3D biochips, microfabricated surfaces were washed with 50 µg/mL gentamicin and then incubated with 0.5% Pluronic for at least 3 h. The 3D biochips were then washed with PBS and were seeded with cells.

### Immunofluorescence of cells in 3D biochips

Cells were seeded onto the 3D biochips and were allowed to adhere and comply with the patterns for at least 24 h. After assay treatments, cells were fixed with 4% paraformaldehyde (Electron Microscopy Sciences) for 15 min at room temperature, washed with PBS, permeabilized with 0.2% saponin for 30 min and blocked with 4% normal goat serum doped with 0.05% saponin for 1 h. Cells were then incubated overnight with primary antibodies (sources and catalog numbers shown in table below) that had been diluted in blocking solution at 4 °C. Cells were washed with PBS and samples were incubated with secondary antibodies (Alexa 488, Alexa 568 and/or Alexa 647) for 1 h at room temperature. For organelle staining, cells were counter-stained with Actin Green and DAPI counterstains in addition to the secondary antibodies. Cells were then imaged on a Zeiss LSM 880 confocal microscope equipped with 63 x 1.4NA oil immersion objective. Same acquisition settings were applied across different conditions that were compared (laser power, gain settings, magnification, zoom, pixel size and slice thickness). For quantitative immunofluorescence of M_3_R, a Z-stack of 30–40 slices using a slice thickness of 0.5 µm were obtained for each cell. Z-stack datasets were then pared down to 21–22 slices encompassing the entire height of the cell (mean cell height ~ 10 µm). Alignment, registration and cropping were performed to ensure each image had the same x–y dimensions (circular cells = 253 × 246 pixels, elliptical cells (AR 1:10) = 106 × 512 pixels). Per condition, images of cells obtained from the same z-plane (3.0 µm from the confocal slice corresponding to the bottom region of the plasma membrane), were averaged to obtain the averaged distribution of M_3_R in different regions of the cell. For immunofluorescence of transcription factors, multi-channel images consisting of DAPI, Actin Green (Invitrogen), and primary antibodies for NFAT, SRF or myocardin were aligned and stitched using the ZEN 2014 software. Image analysis and quantification was performed using ImageJ scripts. Briefly, nuclei were segmented in the DAPI channel. Corresponding cytosol and whole cell objects were outlined utilizing the contrast enhanced phalloidin channel to define cell boundaries. Nuclear-to-cytoplasmic transcription factor ratio was defined as the ratio of the mean transcription factor intensity colocalizing with the nuclear object divided by the mean intensity of the corresponding cytosol object. All measurements were exported directly to csv files and were subsequently analyzed using MATLAB to generate plots.

**Table:** 

Antibody	Dilution	Source
Calnexin	IF 1:100	Abcam, cat # ab22595
Protein disulfide isomerase (PDI) (C81H6)	IF 1:100	Cell signaling*,* cat # 3501
Nogo-A/Reticulon-4	IF 1:100	Cell signaling, cat # ab47085
α-tubulin	IF 1:100	Cell signaling, cat # 2144
AIF mitochondrial marker (D39D2)	IF 1:100	Cell signaling, cat # 5318
EEA1—early endosome marker (C45B10)	IF 1:100	Cell signaling, cat # 3288
RCAS1 (D2B6N) Golgi marker	IF 1:100	Cell signaling, cat # 9091
Muscarinic acetylcholine receptor 3 (M_3_R)	IF 1:100	Abcam, cat # ab126168
NFATc1 antibody	IF 1:100	Abcam, cat # ab2722
SRF antibody	IF 1:100	Cell signaling, cat # 4261
Myocardin	IF 1:100	Abcam, cat # 22073

### Airyscan imaging of live cells

VSMC conforming in the 3D biochips were simultaneously labeled with 1 µM CellMask Plasma Membrane tracker (Life Technologies), 1 µM CellMask ER marker (BODIPY TR Glibenclamide), in HBSS buffer supplemented with 1% Pyruvate, 1% HEPES and 1 mM Trolox, for 5 min at room temperature. Images were acquired using Zeiss LSM 880 using Airyscan super-resolution imaging equipped with 63 x 1.4 Plan-Apochromat Oil objective lens at 30 °C. Z-stacks with an interval of 0.15 µm were collected for the entire cell height which approximated 10–12 µm. Z-stack analyses and other post-acquisition processing were performed on ZEN Black software (Carl Zeiss).

### Calcium measurements

VSMC were seeded on 3D biochips. Calcium measurements in 3D biochips were performed as previously described with modifications^37^. Briefly, cells in 3D biochips were serum-starved for 12 h and loaded with 5 µM of calcium green (dissolved in DMSO) for 30 min at room temperature, with Hanks Balanced Salt solution, (HBSS) supplemented with CaCl_2,_ MgCl_2_ and 10 mM HEPES. Calcium Green was imaged using Zeiss 510 equipped with 40 x Apochromat objective at acquisition frame rate of 4 fps (250 ms acquisition time), and Calcium Green was excited using Argon ion laser 488 at low transmittivity (1%) to prevent photobleaching. Image stacks acquired were then imported into Fiji/ImageJ. Background subtraction was performed on the time stacks by using a rolling ball radius of 50 pixels. Cytoplasm and nuclear regions of interest (ROI) were chosen by performing a maximum intensity projection of the time-stack and specifying a 5 µm radius circle within the nuclear and cytoplasmic regions. To convert intensity values to Ca^2+^ concentration, modified Grynkiewicz equation was used, defined as: $\left[ {{\text{Calcium}}} \right] \, = {\text{ K}}_{{\text{D}}} \left[ {\frac{{\left( {F - Fmin} \right)}}{{\left( {Fmax - F} \right)}}} \right]$. Where *F*_*min*_ is the average fluorescence intensity of the ROI after addition of 100 µM BAPTA AM, *F*_*max*_ is the average fluorescence intensity of the ROI after addition of 0.100 µM A23187. Integrated Ca^2+^ was calculated using the trapz() function in MATLAB.

### FRET imaging

MLCK-FRET plasmid is a kind gift from Dr. James T. Stull (University of Texas Southwestern Medical Center). The MLCK-FRET plasmid is a calmodulin-binding based sensor, where calmodulin binding sequence is flanked with eCFP and eYFP and exhibits decreased FRET upon binding with calmodulin^19,38^. Cells expressing MLCK-FRET were imaged using Zeiss LSM 880 (Carl Zeiss, Jena, Germany), at 37 °C incubator, fitted with Plan-Apochromat 20 x, equipped with 458 nm and 514 nm Argon ion laser lines for excitation of eCFP and eYFP respectively. Incident excitation light was split using an MBS 458 nm/514 nm beam splitter and collected on a 32-spectral array GaAsp detector. The fluorescence emission was collected from 463–520 nm (ECFP), 544–620 nm (FRET channel and eYFP channel). Intensity based ratiometric FRET were obtained using custom-written scripts in ImageJ and MATLAB. Since MLCK-FRET is a single-chain construct, decrease in FRET, and increase in MLCK binding to calmodulin, was expressed as the ratio of emission intensity at 520 nm/emission intensity at 510 nm normalized at the basal levels.

### NFAT imaging

HA-NFAT1(4-460)-GFP was a gift from Anjana Rao (Addgene plasmid # 11107). Patterned cells expressing NFAT-GFP was imaged using Zeiss LSM 880, using Argon ion laser 488 nm, as described above and 63 x 1.4 NA oil objective, with an acquisition rate of 1 frame every 10 s. Time series image stacks were analyzed using ImageJ. Regions of interest of identical size were drawn in the cytoplasmic and nuclear regions of interest and the ratios of these intensities were computed over time.

### Electron microscopy

3D biochips containing fixed A10 cells were embedded in Embed 812 resin (Electron Microscopy Sciences (EMS), Hatfield, PA) using the following protocol. Cells were rinsed in 200 mM phosphate buffer (PB), osmicated with 1% osmium tetroxide/PB, washed with distilled water (dH20), and *en bloc* stained with aqueous 2% uranyl acetate, washed with dH2O and dehydrated via increasing ethanol (ETOH) series /distilled water (25%, 60%, 75%, 95% and 100% ETOH). Cells were further dehydrated using propylene oxide (PO), and embedded using ascending PO:EPON resin concentrations (2:1, 1:1, 1:2, pure). Prior to solidification, the coverslips were placed on 1″ x 3″ microscope slides, and multiple open ended embedding capsules with a 1 × 1 cm face (EMS) were placed on the coverslips covering the areas of interest. The resin was then polymerized in a vacuum oven at 65 °C for 8–12 h. After the first layer was solidified, the capsule was topped off with more resin and put back in the oven for another 8–12 h. Capsules containing cells within 3D biochips were removed from coverslips using previously described methods^39^. Briefly, to separate the block from the coverslip, a hot plate was heated to 60 °C and the microscope slide was placed directly on a pre-heated hot plate for exactly 3 min and 30 s. The slide was removed from the hot plate and the capsules carefully dislodged free from the coverslips. Once separated, the block face retains the cells within the 3D biochips. The block was coarsely trimmed with a double-edged razor blade, and a Diatome cryotrim 45° mesa-trimming knife (EMS) was used to finely trim the block. Using as large a block face as possible, 70 nm ultrathin sections were cut from the block surface using an ultra-thin diamond knife (EMS), and a Leica EM UC7 ultramicrotome (Buffalo Grove, IL). All sections coming off the block face were collected. Sections were collected using a Perfect Loop (EMS) and transferred to a 2 × 1 mm formvar-carbon coated reinforced slot grid (EMS). The sample was dried on the grid and transferred to Hiraoka Staining Mats (EMS) for heavy metal staining. Grids were stained with 3% uranyl acetate in water for 40 min, washed and stained with Reynold’s lead citrate for 3 min, washed and allowed to dry. Electron microscopy images were taken using a Hitachi 7000 Electron Microscope (Hitachi High Technologies America, Inc.) equipped with an AMT Advantage CCD camera. Cells were viewed at low-magnification to identify areas of interest (cell tip versus cell body) before high magnification imaging. Images were transferred to Adobe Photoshop CS3 (version 10), and adjusted for brightness and contrast. Measurement of plasma membrane to ER distances from electron microscopy images were performed blindly. Briefly, sample information from images were removed and images were saved with a randomized filename. Image contrast was further enhanced using ImageJ using contrast-limited adaptive histogram equalization (CLAHE). Only images with discernible smooth ER closely apposed to the plasma membrane were analyzed and distances were measured at optimal xy orientations at 50 nm intervals using ImageJ. Data was graphed using MATLAB. For 3D reconstruction of ER-PM distances, 3D serial block face scanning electron microscopy was performed following the above protocol with the following modifications: prior to sample embedding, high-contrast tissue pre-staining was performed using 2% Osmium tetroxide, 2.5% potassium ferricyanide, 1% thiocarbohydrazide, and 1% uranyl acetate in cacodylic buffer. Blocks were serial sectioned and imaged at 1600 × magnification using a 3View Gatan system coupled with a Zeiss Gemini FE scanning electron microscope (Carl Zeiss Microscopy, LLC). Final tiled pixel frame store of 32 k-by-32 k with a 650 µm field of view lead to lateral and axial resolutions of 20 and 75 nm, respectively. ER-PM distances were manually segmented for each axial image by selecting dyads perpendicular to the cell surface in a blinded fashion.

### Statistics

Results are presented as mean ± standard error of the mean from at least three independent experiments. Normality was determined using Shapiro–Wilk test using a *p* value ≥ 0.05. If the distribution was normal, a two-tailed Student’s t-test was performed. For datasets with non-normal distribution, two-tailed Mann–Whitney test was used. *p* < 0.05 was considered statistically significant.

### Model development

We formulated a phenomenological model to study the role of PM–SR distances. The complete derivation and mathematical solution is given in the Supplementary Information. Complete details of the simulations using finite-element methods in COMSOL and using finite-volume methods in *Virtual Cell* are given in the Supplementary Information.

## Results

### Reaction–diffusion model predicts that the distance between the PM and SR membrane affects signaling dynamics in the cytoplasmic volume

**Figure 1:**
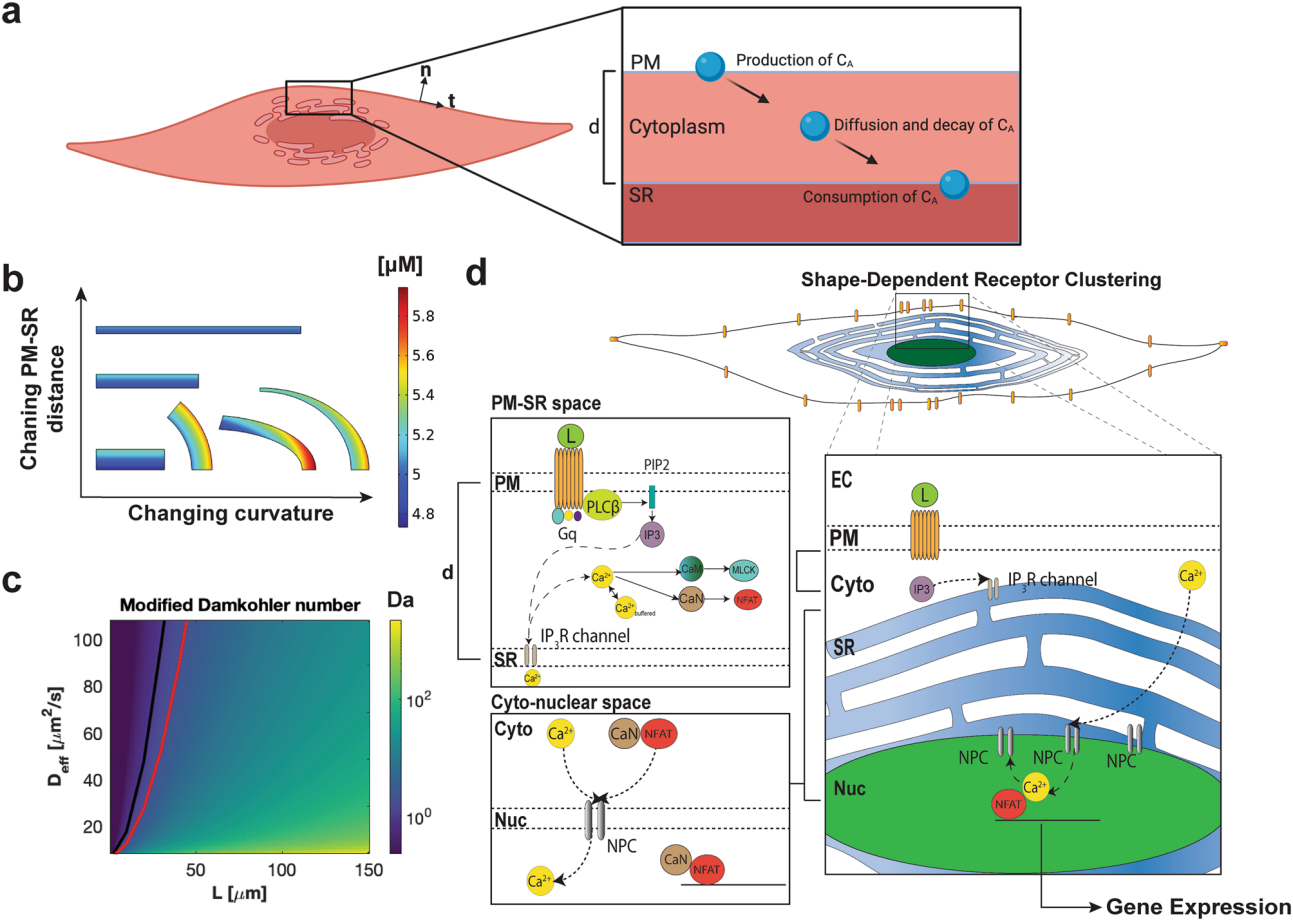
Phenomenological model describing the dynamics of a signaling molecule produced at a membrane and consumed at an organelle membrane compartment as a function of curvature and PM–SR distance. (**a**) Dynamics of a signaling molecule can be described by its production, diffusion, degradation in the cytoplasmic space, and consumption at an intracellular organelle membrane. Both curvature (denoted as t and n) and PM–SR distance differences (denoted as d) can play important roles in signaling dynamics. The interplay between sources, sinks, and boundary fluxes determines signaling dynamics. Schematic created with Biorender.com. (**b**) Six different geometries with the same area were used to test how curvature of the membrane and distance between the plasma membrane (PM) and the inner membrane, a sarcoplasmic reticulum (SR) in this case, affect the spatio-temporal dynamics of the signaling molecule ‘C_A_’. Reaction–diffusion modeling in COMSOL predicts that curvature and distance between the PM and SR will both affect the spatial distribution of C_A_. Here we show transient snapshots at 50 ms for a toy model of C_A_ as defined in Eqs. 1–3 where k_deg_ = 0.5 [1/s], k_on_ = k_off_ = 7.5e−10 [μm/s], D_Ca_ = 1 [μm^2^/s], and Ca_cytoIC_ = 5 [μM] is the initial concentration of calcium everywhere in the cytosol. The top surface for the rectangles and rightmost surface for the curved sections is the PM as the emission surface, and the bottom surface for the rectangles and leftmost surface for the curved sections is the SR membrane as the absorption surface. The concentration ranges from ~ 4.7 to 5.9 μM. (**c**) The modified Damkohler number for different values of PM–SR distance, ‘L’, and effective diffusion coefficient, ‘D_eff_.’ k is taken as 0.1 1/s in this example. For physiological distances shown in this study, the dynamics of A are dominated by diffusion. The threshold between reaction-dominated and diffusion-dominated regimes is defined at Da = 1, shown as a black line. If a system has diffusion-trapping, for the same diffusion coefficient, the system can shift regimes due to geometric and kinetic trapping factors (compare black line to red line). Therefore it is clear that considering effective diffusion could shift the system between a diffusion-dominated to reaction-dominated system, particularly for small crowded intracellular regions. (**d**) Biochemical events that govern Ca^2+^ signaling in VSMC including GPCR-G_αq_ cycling on the membrane, PLCβ activation, IP_3_ production and IP_3_R-mediated Ca^2+^ release from SR.

We tested the hypothesis that a change in membrane curvature and distance between two membranes affects signaling dynamics using a phenomenological reaction–diffusion model in COMSOL (Fig. 1a). A signaling molecule of interest, C_A_, is produced at the PM with an on-rate k_on_ (μm/s) and binds to a receptor located at the SR membrane, with a rate k_off_ (μm/s) and is free to diffuse in the sandwiched cytoplasmic space and is degraded by a degrading enzyme with a rate k_deg_ (1/s). This model essentially captures the lifecycle of a second messenger such as IP_3_ that is produced at the PM through PIP_2_ hydrolysis by phospholipases, binds to inositol 3 phosphate receptor (IP_3_R) channel at the SR membrane and is degraded in the cytoplasm by 1,4,5-trisphosphate-5-phosphatase^32^. These events can be mathematically represented by the following system of reaction–diffusion equations. The dynamics of C_A_ in the cytoplasm are governed by diffusion and degradation and is given by,

1 $$ \frac{{\partial C_{A} }}{\partial t} = D\nabla^{2} C_{A} - k_{\deg } C_{A} $$

where D is the diffusion coefficient of C_A_ (μm^2^/s), and C_A_ is the concentration of C_A_ (μM). The boundary condition at the PM is a balance between the rate of production of C_A_ at the membrane and the diffusive flux from the membrane to the cellular interior and is given by,

2 $$ D({\mathbf{n}} \cdot \nabla C_{A} )|_{PM} = k_{on} C_{A} |_{PM} $$

here n is the normal vector to the surface and ∇ represents the gradient operator. At the SR membrane, similarly, we can write the boundary condition for the consumption of C_A_ as the balance of diffusive flux to the SR and consumption rate at the SR.

3 $$ - D({\mathbf{n}} \cdot \nabla C_{A} )|_{SR} = k_{off} C_{A} |_{SR} $$

We note that C_A_ = 0 is the trivial solution to this system, so we assume a nonzero initial condition. For illustrative purposes, we solved these equations using finite element methods on six geometries (1) a control rectangle (constant distance, zero curvature) (2) two additional rectangles with different PM–SR distances, (constant distance, zero curvature), (3) a circular sector (constant distance, constant curvature), and (4) an elliptical sector, (constant distance, varying curvature), and (5) a elliptical sector (varying distance, varying curvature) (Fig. 1b). In cases (1–3), the gradient of C_A_ is only along the normal direction (Fig. 1b). Cases (4–5), where curvature varies and both curvature and PM–SR distance vary, results in two-dimensional gradients. The curvature of the membrane and the PM–SR distance will affect both the production and consumption of C_A_ at the PM and SR respectively. Hence, C_A_ varies both in the radial and angular directions, indicating that curvature and varying distances between the two membranes amplifies signaling gradients. Even though the observations here are in 2D, the effect of curvature and distance holds true in 3D as has been demonstrated by us and others^3,40,41^.

Dimensional analysis of Eq. 1 produces the Damkohler number ($k_{deg} L^{2} /D$), a nondimensional number relating the contribution of reaction rate to diffusive transport, which identifies the system as either diffusion- or reaction-dominated^42^. Considering the model system, species C_A_ travels a distance, L (d in Fig. 1), from the PM to the SR during which time it can be degraded at some rate k_deg_. However, the cell cytoplasm is a dense mixture of proteins, ions, and other cellular components that can interact with and trap species C_A_ in various direct or indirect ways^43,44^. This trapping, whether through binding or diffusion barriers, produces an effective diffusion coefficient that can be written as $D_{eff} = \frac{D}{{1 + \gamma_{geo} \left( {1 + \beta_{kin} } \right)}}$, where $\gamma_{geo}$ represents the geometric effects of traps and $\beta_{kin}$ represents kinetics of binding at traps if applicable^43^. Inserting this effective diffusion coefficient into the Damkholer number provides a more realistic analysis of the species C_A_, accounting for diffusion trapping, consistent with approaches from statistical physics. Diffusion-trapping can modify the effects of diffusion however, altering the dynamics of the system. In Fig. 1c, the black line denotes Da = 1, the usual threshold between reaction-dominated and diffusion-dominated regimes. If one considers two cases for the same diffusion coefficient, one without diffusion-trapping (black line) and one with diffusion-trapping (red line), it is clear that considering effective diffusion can shift the system between reaction- and diffusion-dominated regimes, particularly in crowded intracellular regions.

From this simple phenomenological model, we predict that second messenger signaling pathways such as receptor/phospholipase C/IP_3_, where signaling occurs between PM and SR, will be impacted by both cell shape and distance between the PM and the SR. This prediction raises the following questions: (1) does cell shape affect PM–SR distance? (2) Does changing PM–SR distance affect intracellular signaling dynamics? And (3) what is the impact of changing the distances between organelles on VSMC contractility through MCLK activity?

We asked whether changing the PM–SR distances can impact the dynamics of IP_3_ signaling. Since it is currently not possible to experimentally manipulate PM–SR distances with precise control in cells, we used numerical models of IP_3_ and Ca^2+^ in the context of VSMC to study the relationship between PM–SR distances and IP_3_ dynamics. This model is composed of a system of multi-compartmental partial differential equations, representing the reaction–diffusion of cytoplasmic species in the volume, coupled with boundary fluxes at the membrane. The reactions capture the biochemical interactions from the ligand binding and activation of M_3_R to IP_3_ production by PLC-mediated hydrolysis of PIP_2_ release from the SR (Fig. 1d, subset of Supplementary Table 1–2, R1–R16). The simulations were conducted in the commercially available finite element software COMSOL to enable investigation of small PM–SR distances ranging from 50 to 150 nm.

**Figure 2:**
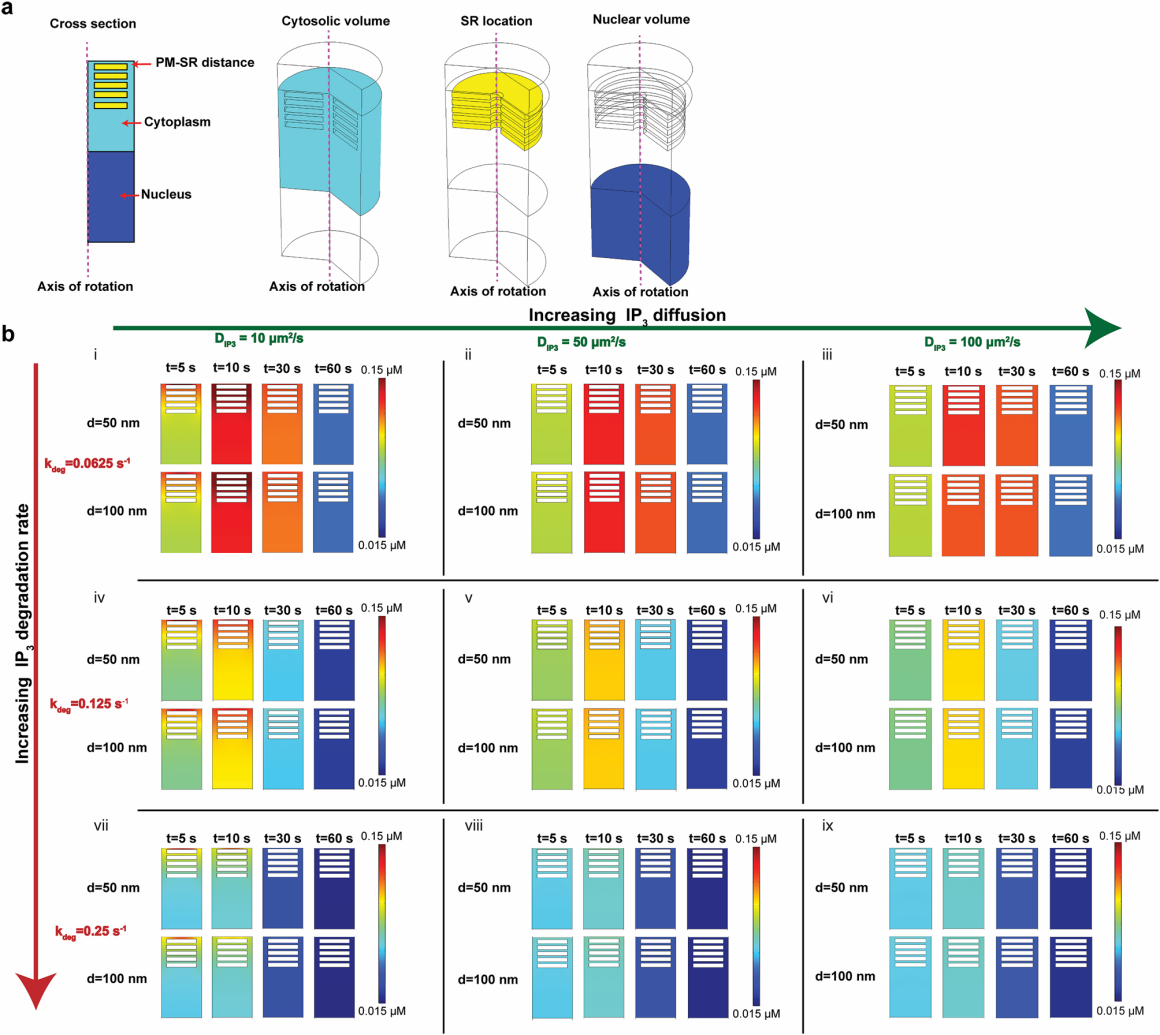
Small length scale simulations in COMSOL show a critical relationship between IP_3_ and PM–SR distance. (**a**) Cross-section and geometries representing different compartments governing biochemical events. We model a small part of the cell as a cylinder. The cytosolic volume is shown in cyan, the SR modeled as cylindrical disks shown in yellow and the nucleus is shown in blue and the axis of symmetry is shown in pink. The PM–SR distance, denoted as d in Fig. 1d, is measured between the plasma membrane and the first SR disk. (**b**) Effect of IP_3_ diffusion and degradation rate are shown for two different distances between the PM and the SR. Panels (i–iii) show the effect of increasing IP_3_ diffusion [(i) 10 μm^2^ s^−1^, (ii) 50 μm^2 ^s^−1^, and (iii) 100 μm^2^ s^−1^] on IP_3_ concentration at 5 s, 10 s, 30 s, and 60 s for a degradation rate k_deg_ = 0.0625 s^−1^ for two different values of d (50 nm and 100 nm). Panels (iv–vi) show similar calculations for k_deg_ = 0.125 s^−1^ and panels (vii–ix) for k_deg_ = 0.25 s^−1^.

In order to investigate how PM–SR distances affect IP_3_ dynamics, we constructed a cylindrical geometry to represent a portion of the cell and the ER as discs (Fig. 2a). This idealized geometry enables us to study reaction–diffusion in 3D using COMSOL while gaining insight into the stacked ultrastructure of the SR. We varied the following parameters: (a) PM–SR distance, (b) diffusion constant of IP_3_, and (c) IP_3_ degradation rate. These three parameters govern the production, diffusion, and consumption of IP_3_. Our results can be summarized as follows—across all parameter variations, shorter distances between PM–SR gave rise to high IP_3_ gradients (Fig. 2b i,iv,vii, Supplementary Fig. 7, Supplementary Fig. 11). Increasing the diffusion coefficient resulted in rapid dissipation of the gradient and increasing IP_3_ degradation rate resulting in a lower concentration of IP_3_, as expected. We found that the shortest distance between the PM–SR (50 nm), with the smallest diffusion coefficient (10 μm^2^/s), and largest IP_3_ degradation rate (0.0625 s^−1^) (Fig. 2b vii, top row) had the strongest IP_3_ gradient at 5 s. Increasing the PM–SR distance to 100 nm altered the extent of the observed IP_3_ gradient at 5 s (Fig. 2b i, bottom row) but did not alter this gradient very much at later times. From these simulations, we predict that changing the distances between organelles can alter IP_3_/Ca^2+^ signaling dynamics and potentially VSMC contractility as measured by MLCK activity. We used VSMCs grown on 3D biochips to test these predictions.

### Cell shape also affects cytoskeleton organization

**Figure 3:**
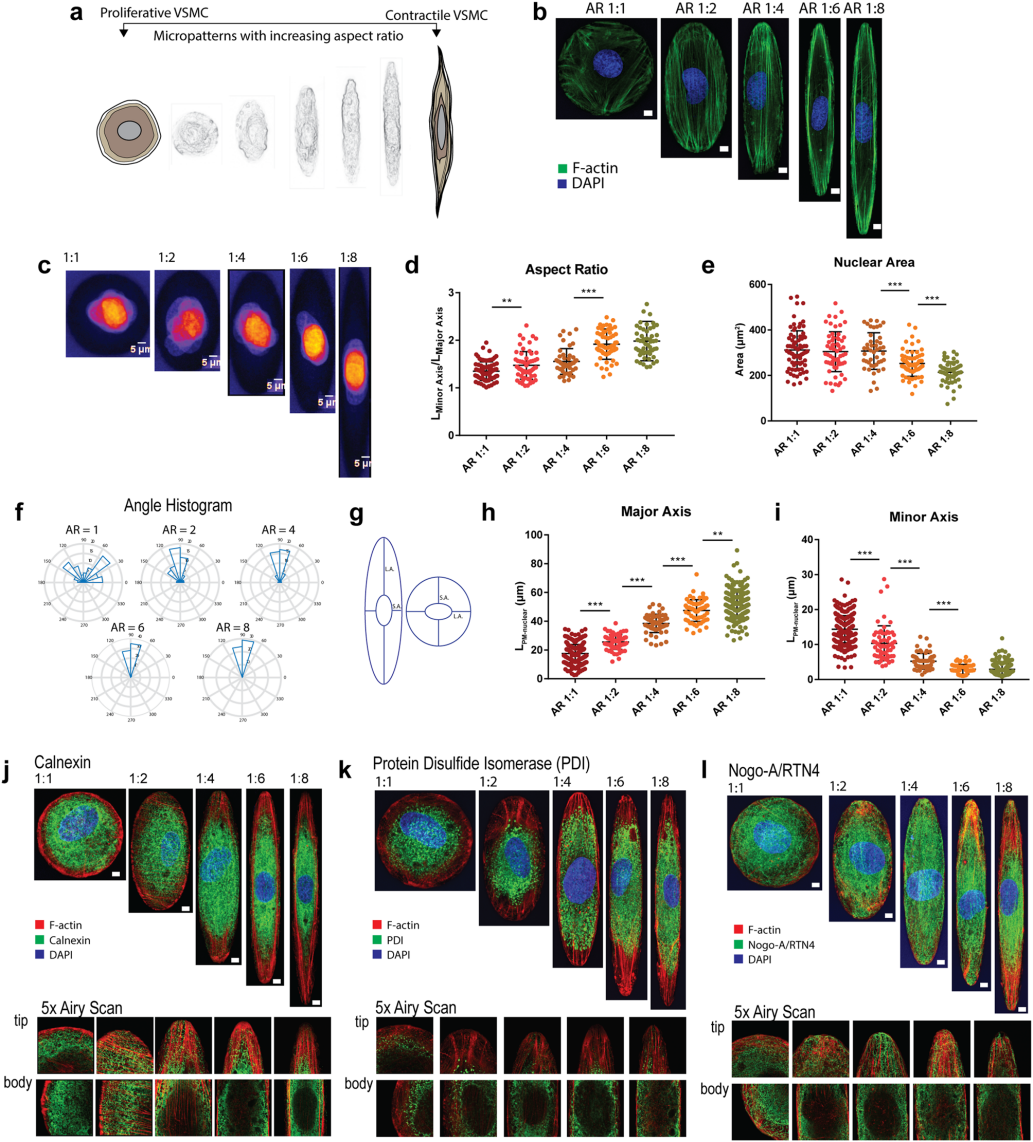
Cell shape impacts cytoskeletal organization and organelle location. Whole cell shape changes location and distribution of subcellular organelles in a systematic manner. (**a**) Biomimetic, microfabricated surfaces with graded aspect ratios fine-tune the shape of VSMC and its enclosed organelles. (**b**) Actin organization is dependent on cell shape. (**c**) Cell shape affects nuclear shape, size and distance to PM. Representative images of VSMC compliant in patterns with increasing AR stained for DAPI. Scatterplots of whole aspect ratio versus (**d**) nuclear aspect ratio (**e**) size (area µm^2^) (**f**) orientation with respect to the major axis of the cell (*N*_*AR1:1*_ = *82*, *N*_*AR1:2*_ = *60*, *N*_*AR1:4*_ = *45*, *N*_*AR1:6*_ = *65*, *N*_*AR1:8*_ = *54*). (g) Relationship between whole cell shape and PM-nuclear distance was determined by segmenting cell and nuclear boundaries with Actin Green and DAPI respectively. Distances between nuclear and plasma membrane boundaries were measured along the long and short axes of the nucleus. (**h**) Increasing the cellular aspect ratio increases the distance between the nucleus and the plasma membrane in the major axis of the cell while (**i**) decreasing the PM-nuclear distance in the minor axis of the cell (**p ≤ 0.05, ***p ≤ 0.0001, two-tailed t-test). (**j**–**l**) SR distribution in VSMC seeded in AR 1:1 to 1:8 staining for SR membrane markers (panels below show 5 x magnified Airy scan images of tip and body of images shown) (**j**) calnexin (**k**) protein disulfide isomerase (PDI) and (**l**) reticulon-4. Scale bars shown are 10 µm.

We determined if changing the cell shape affects the organization of the cytoskeleton and the distribution of other organelles. In order to control the large scale cell shape, we used 3D biochips with the same surface area but increasing aspect ratio (AR, circles 1:1 to ellipses 1:8) (Fig. 3a). We investigated how cell shape affects cytoskeletal organization since the two are tightly interwoven^45–47^. Actin stress fibers increasingly oriented themselves along the long axis of the cell as the aspect ratio increased (Fig. 3b), indicating that the cells were responding to the mechanical forces and tension exerted by the substrate^48^. It has been shown in several cell types that nuclear shape is tightly coupled to cell shape^49^. Nuclear aspect ratio increased with cellular aspect ratio (Fig. 3c,d) while nuclear size (area, in µm^2^) decreased with increasing cell aspect ratio (Fig. 3e). The nuclei became increasingly oriented along the major axis of the cell as the whole-cell aspect ratio increased, evidenced by polar graphs showing the orientation histograms of nuclei of cells in increasing cell AR (Fig. 3f). Circular shaped cells showed a random nuclear orientation whereas increasing the cellular aspect ratio progressively oriented the nucleus in the geometric center of the cell. PM-nuclear distance in the major axis of the cell increased with cell aspect ratio (Fig. 3h) while the PM-nuclear distance in the minor axis decreased with cell aspect ratio (Fig. 3i). These results indicate that in VSMC, cell elongation resulted in nuclear elongation, reduced nuclear size, and a decrease in the PM-nuclear distance in the minor axis of the cell (i.e. near the center of the cell). In addition to the nucleus, spatial arrangement of the microtubules also changed with aspect ratio. Microtubules became highly aligned and increasingly sparser in the cell tips compared to the cell body as the cell aspect ratio increased (Supplementary Fig. 1). Because cytoskeletal organization plays an important role in the distribution of many organelles^50,51^, we visualized the effect of cell shape on the bulk distribution and location of the mitochondria (Supplementary Fig. 2), endosomes (Supplementary Fig. 3) and Golgi membrane (Supplementary Fig. 4). No clear trends were seen in the distribution of these organelles for different cell shapes. It has been reported that well-differentiated VSMCs have a characteristic central distribution of the endomembrane system^25^. Because SR stores Ca^2+^, which controls both excitation-transcription coupling and contractility in VSMC^52^ , we focused on SR distribution as a function of cell shape, using calnexin (Fig. 3j), protein disulfide isomerase (Fig. 3k), reticulon-4 (Fig. 3l) and bodipy glibenclamide (Supplementary Fig. 5) as SR markers. All four markers show that in circular cells, the SR was spread uniformly throughout the cell; increased aspect ratio induced the SR to localize in the perinuclear region and become significantly sparser, and mainly tubular, in the cell tips (Fig. 3j–l, Supplementary Fig. 5). Upon close inspection of Airy scan images of these SR markers, we qualitatively observe that in circular cells, the SR appeared to be equidistant from the plasma membrane along the periphery, i.e. there were no angular variations of PM–SR distance, while elliptical cells show a large angular variation in the PM–SR distance (Fig. 3d,e bottom panels and Supplementary Fig. 5, inset). Thus, we show that cell shape affects not only cytoskeletal organization, but also affects nuclear shape and PM-nuclear distances, and organelle distribution.

**Figure 4:**
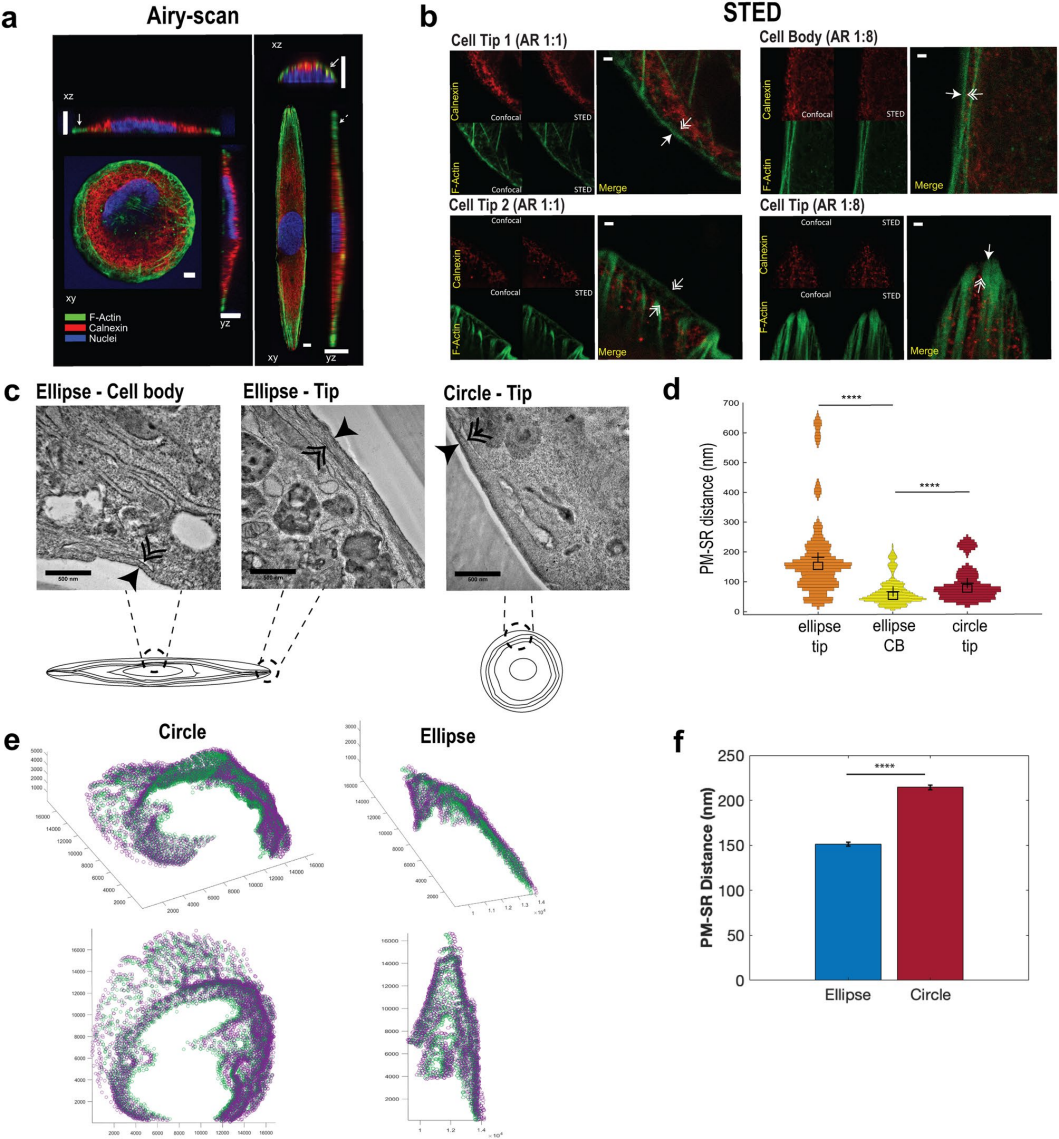
Shape impacts PM–SR distances in 3D. (**a**) (Left panel) 3D confocal volume scans of VSMC cultured in circle and ellipse (Aspect ratio of 1:8) patterns showing F-Actin (green), ER membrane marker calnexin (red) and nuclei (blue). Increased co-localization of red and green pixels are seen in the xz (short axis) cross-section of elliptical cells (solid double arrow heads), while little co-localization is evident in tips of both circular and elliptical cells (solid single-arrow heads and dashed single-arrow heads, respectively). Scale bars shown are 10 µm. (**b**) (Right panel) Super-resolution STED imaging of F-actin (green), calnexin (red), in cell tips of circular cells (AR 1:1) and in cell tip and cell body of elliptical cells (AR 1:8). Scale bars shown are 1 µm. (**c**) Representative TEM micrographs sampled from corresponding regions in micropatterned cells (cartoon). Single-headed arrows indicate the plasma membrane and double-arrows indicate smooth, peripheral SR, apposed to the plasma membrane. Scale bars shown are 500 nm. (**d**) Distribution plots of PM–SR distances obtained from TEM images, mean and median are shown as crosses and squares respectively. (*N*_*ellipse*_ _*tip*_ = 329, *N*_*cell*_ _*body*_ = 254, *N*_*circle*_ _*tip*_ = 115, ****p < 0.0001, two-tailed Mann–Whitney test). (**e**) In order to quantify the 3D distribution of PM–SR distances, spatial dyads were manually segmented in each axial image for representative circular and elliptical cells. Green points represent the SR segments while purple points represent the PM. (**f**) The average PM–SR distance and standard error mean (SEM) for a representative circular and elliptical cell was computed from the 3D serial block face scanning electron microscopy images (N_ellipse_ = 1692, N_circle_ = 5309, N refers to PM–SR distances taken within the representative cells, ****p < 0.0001, two sample t-test).

**Figure 5:**
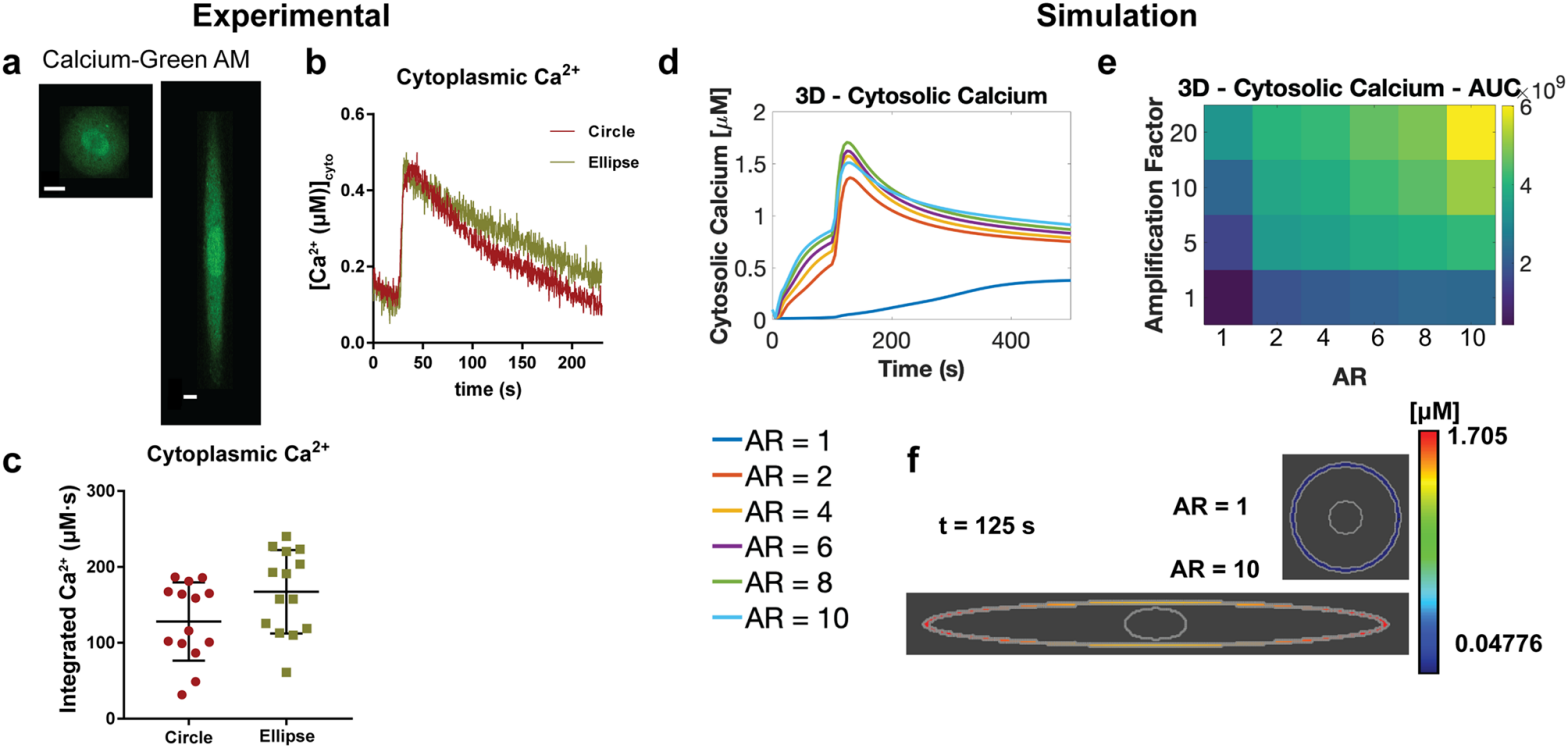
Cell aspect ratio affects cytosolic calcium levels. (**a**) Circular and elliptical cells were loaded with Calcium-Green AM dye, to measure Ca^2+^ signaling in response to 10 µM carbachol. Scale bars shown are 10 µm. (**b**) average time-course of cytoplasmic Ca^2+^ in response to CCh (circle, N = 14, oval, N = 14, stimulated at arrow indicated) (**c**) Scatterplot of cytoplasmic integrated Ca^2+^ (area-under-the-curve) per cell in circular and elliptical cells. Temporal (**d**) and AUC results (**e**) of cytosolic calcium for simulations of various aspect ratios and SR flux amplification factors in *Virtual Cell*. Inset below: legend for temporal simulation results showing the various aspect ratios, AR. (**f**) Cytosolic calcium spatial results for AR of 1 and 10 at 125 s.

### Cell shape changes PM–SR distances in 3D

Two-dimensional images of peripheral SR membrane and plasma membrane indicate that cell shape alters SR abundance and PM–SR distance on the equatorial region (Fig. 3j–l). We investigated more closely whether PM–SR distances are altered by the global shape of cells in 3D. Confocal volume scans of VSMC cultured in circular and ellipse patterns show increased colocalization between calnexin and plasma membrane stains in the equatorial region of elongated cells, as opposed to the cell tips of elongated cells and circular cells, suggesting increased proximity of the two membranes at the equatorial region of elongated cells (Fig. 4a). Superresolution STED measurements reveal that SR puncta are closer to the cell cortex in the equatorial region of elongated cells (upper right panel, Fig. 4b), compared to both cell tips of elongated and circular cells (upper left and lower right panels, Fig. 4b). To quantitatively confirm that the PM–SR distance is shape dependent, we used transmission electron microscopy to visualize peripheral SR to PM distance (Supplementary Fig. 6). TEM showed that the cell periphery of circular cells and the cell body of elliptical cells showed long patches of smooth SR that were positioned close to the plasma membrane. PM–SR distance was indeed dependent on the shape of the cell: PM–SR distance in the cell body of elliptical cells was significantly smaller compared to circular cells (Fig. 4c). In the tips of elliptical cells, the SR membrane formed fewer contacts with the plasma membrane, and showed significantly higher PM–SR distance (Fig. 4d). 3D serial block face scanning electron microscopy reveals that the distance between puncta on the PM and SR is aspect ratio dependent (Fig. 4e left versus right). The difference between SR–PM distance in circular and elliptical cells was greatest within the first few microns of the basal surface, and it was progressive reduced towards the apical surface (Supplementary Fig. 20). However, we observed that for a given cell shape, the effect across was small when compared to differences between cell shapes (Fig. 4f). These results are consistent with the recent observation in neurons that PM–ER contacts are more extensive in the cell body compared to elongated projections such as dendrites and axons^53^. While other groups have reported that cell shape can affect organelle location^49,54^, here, we quantitatively determine PM–SR distances and the SR abundance along the juxtamembrane region with controlled cell shape variation.

**Figure 6:**
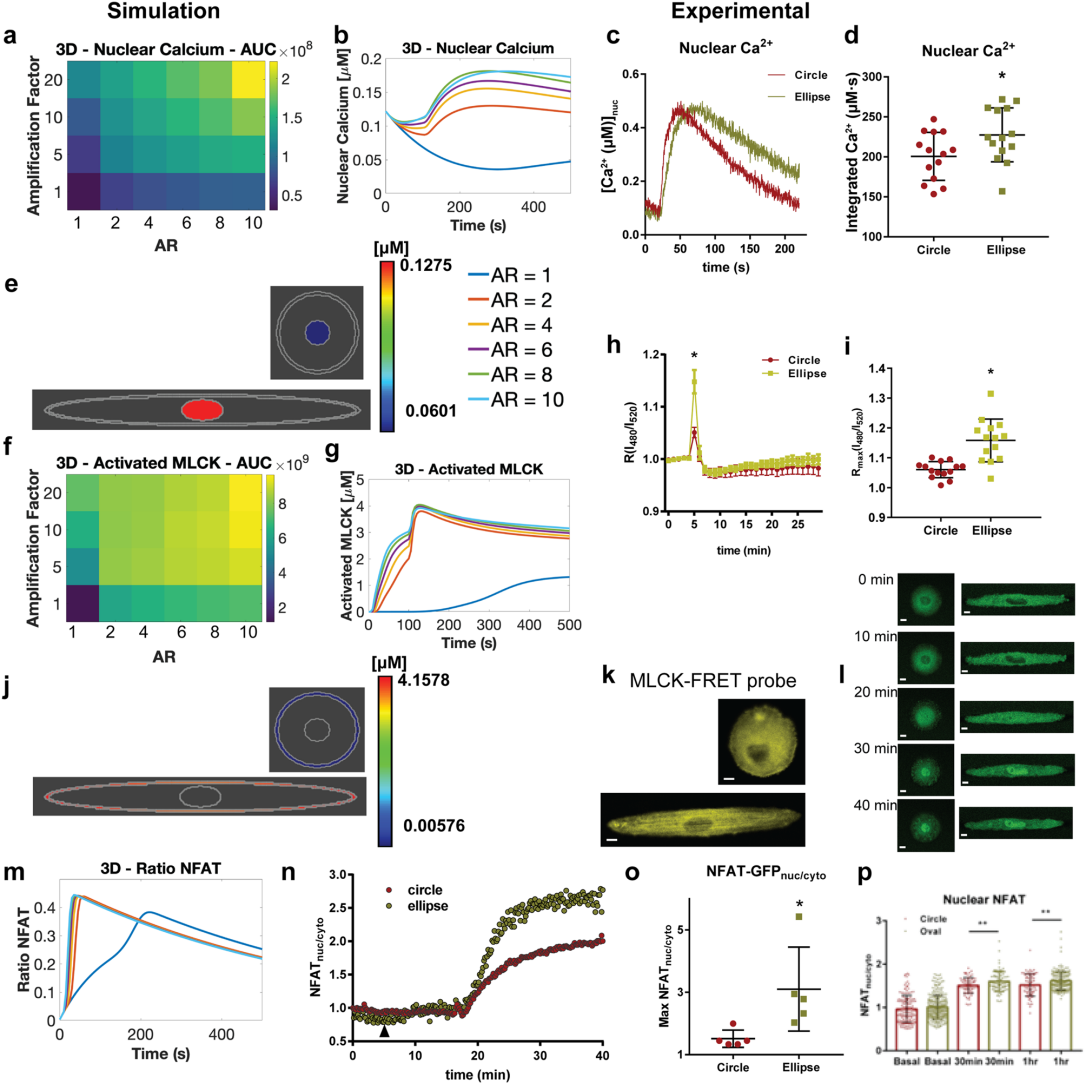
Downstream signaling effects of shape on nuclear Ca^2+^/MLCK/NFAT. (**a**) Simulation results in *Virtual Cell* for effects of SR amplification factor and AR on nuclear calcium AUC. (**b**) Temporal dynamics of nuclear calcium for models with various ARs. (**c**) Average time-course of nuclear Ca^2+^ in response to CCh (**d**) scatterplot of nuclear integrated Ca^2+^ (area-under-the-curve) in circular and elliptical VSMC. (*p = 0.035, two-tailed t-test). (**e**) Simulation spatial results of nuclear calcium at t = 125 s for AR = 1 (upper panel) and 10 (lower panel). Right inset: legend for all temporal simulation results (**b**,**g**,**l**). (**f**) Effects of SR flux amplification factor and AR on activated MLCK AUC. (**g**) Temporal dynamics of activated MLCK for various ARs. (**h**) Temporal MLCK activation in circular and ellipse VSMC upon stimulation with carbachol, activation was measured by decrease in FRET and increase in R480/520 (**i**) scatterplot of maximal R(I_480_/I_520_) from individual cells expressing MLCK-FRET probe. (*p ≤ 0.0001, two-tailed Mann Whitney test). (**j**) Spatial results of activated MLCK at t = 125 s for AR = 1 (upper panel) and 10 (lower panel). (**k**) Circle and elliptical cells expressing MLCK-FRET probe. (**l**) Representative VSMC complying to circle and elliptical micropatterns expressing NFAT-GFP, in basal and after stimulation with carbachol. (**m**) Model results for NFAT ratio for different ARs. (**n**) Representative NFAT-GFP time-course (**o**) scatterplot of maximal NFAT_nuc/cyt_ ratio in circle and elliptical cells. Max NFAT^circle^_nuc/cyto_ = 1.51 ± 0.12, *N* = *5*, Max NFAT^ellipse^_nuc/cyto_ = 3.10 ± 0.60, *N* = *5,* *p* value = 0.0325, two-tailed t-test (*p = 0.0325, two-tailed t-test, N = 5 for each group). (**p**) Single cell quantification of endogenous NFAT translocation (NFAT_nuc/cyt_) in basal and M_3_R stimulated states, in circle and ellipse VSMC. Bar plots shown are mean ± SD with corresponding dot plot overlays (at basal, *N*_*circle*_ = 138,* N*_*ellipse*_ = 284, at time = 30 min, *N*_*circle*_ = 72,* N*_*oval*_ = *81*, **P = 0.024, at time = 1 h, *N*_*circle*_ = 69,* N*_*oval*_ = 193, **p = 0.010, two-tailed Mann–Whitney test). Scalebars shown are 10 µm.

### Cell shape affects receptor activation on the membrane and intracellular calcium dynamics

We tested the model predictions that distance between PM and SR can affect the dynamics of Ca^2+^ mediated by the M_3_R/IP_3_/Ca^2+^ pathway^14,55^ using computational and experimental methods. We stained for M_3_R in circular and elliptical cells under three different conditions—unstimulated, stimulated with carbachol, and stimulated with carbachol in the presence of hypertonic sucrose, which inhibits receptor endocytosis^56–58^. We also constructed 3D spatial models of different aspect ratios in *Virtual Cell* (VCell) from carbachol stimulation to MLCK activation (see Supp for model details). We switched from COMSOL to *Virtual Cell* for the following reasons: *Virtual Cell* allows for experimental cell images to be imported and used as geometries for the computational simulations (see Supplementary Fig. 10 and Material for more information on model geometries). This allows us to make closer comparisons between experiments and simulations. *Virtual Cell* is also computationally less expensive than COMSOL and is compatible for running simulations that involve whole cells. While we are unable to resolve the PM–SR distances in great detail in *Virtual Cell*, the benefits of conducting simulations in realistic geometries and for the entire signaling cascade were deemed significant to make the switch.

Experimental results show that in both the basal state and stimulated states, M_3_R was uniformly distributed on the plasma membrane of both circular and elliptical cells (Supplementary Fig. 8). Interestingly, in elliptical cells, when M_3_R was stimulated and endocytosis was inhibited, M_3_R accumulated in the cell body compared to the cell tips while there was no observable spatial asymmetry in the distribution of M_3_R in circular cells (Supplementary Fig. 8 and 12), consistent with our previous observations and modeling^3^. We then investigated the effect of shape on cytoplasmic Ca^2+^ dynamics upon stimulation of M_3_R in patterned VSMC (Fig. 5a–c). In the cytoplasmic region, circular and elliptical showed similar peak Ca^2+^ amplitudes. However, elliptical cells showed a slower rate of decrease in Ca^2+^ compared to circular cells, resulting in a reproducibly observable higher temporally integrated Ca^2+^ compared to circular cells, although the differences were near the threshold for statistical significance (*p* = 0.057, two-tailed t-test n = 14).

Recent ultrastructural analyses have shown that the ER is a highly dynamic, tubular network that occupies roughly 10% of the cytosolic volume and extends from the nucleus to the cell periphery^59^. Currently, geometries used in spatial models typically do not capture the complexity of the organelle ultrastructure^53^, however, advancements in meshing algorithms and modeling pipelines pave the way for more complex geometries as model inputs^60,61^. To represent the potential effect of the convoluted ultrastructure of the SR membrane, we introduce an SR flux amplification factor to our model. This amplification factor scales the SR flux term for IP_3_ activated calcium release from the SR, R17 in Supplementary Table 1^39^. This amplification factor provides some insight into how the spatial system would be affected by a modified flux term due to differences in SR geometry, specifically folds in the SR membrane, and allows us to explore the signaling consequences of varying the SR surface area. As a result, we can investigate the role of larger flux through the SR membrane without explicitly changing the SR model geometry (see Supplementary Material for more details). Model results (Fig. 5d–f) show a similar trend of higher cytoplasmic Ca^2+^ in larger ARs compared to smaller AR. A parametric sensitivity analysis of the model reveals that the rate of Ca^2+^ release from the SR due to IP_3_R is a key parameter in the model (Supplementary Figs. 16–19). We investigate this geometric complexity through the aforementioned amplification factor. Increasing this amplification factor, which effectively captures a large surface area of the SR, also leads to increasing cytoplasmic Ca^2+^. Therefore, cell shape, SR surface area, and the PM–SR distance together influence short-timescale signaling events associated with the muscarinic receptor pathway.

### Cell shape affects downstream signaling dynamics

Since small changes in calcium signals can have large functional effects as a result of amplification by downstream signaling networks^62^, we predicted that changes in cytoplasmic calcium levels can lead to additional downstream signaling changes. Our model predicts that higher ARs and larger shape factors will lead to large nuclear Ca^2+^ peaks and AUC (Area under the curve) (Fig. 6a,b,e). AUC is a common measure of signaling over time that quantifies accumulated signaling in the cell^63^. We measured downstream effector activities in the cytoplasm and in the nucleus to verify these predictions experimentally. We first measured nuclear Ca^2+^ levels in circular and elliptical cells (Fig. 6). The differences in nuclear Ca^2+^ between circular and elliptical cells were distinct from cytoplasmic Ca^2+^ (Fig. 6c). In the nuclear region, the peak Ca^2+^ amplitudes of circular and elliptical cells were similar. However, there is a notable delay in the rate of nuclear Ca^2+^ increase to maximum in elliptical cells compared to circular cells, and the decay times in elliptical cells were slower as well, resulting in a significantly higher concentrations of temporally integrated Ca^2+^ in elliptical cells compared to circular cells (Fig. 6d). These results indicate that cell shape affects nuclear Ca^2+^ transients triggered by M_3_R/PLCβ and that such transients are more prolonged in elliptical cells. We similarly predicted elevated activated MLCK AUC and peaks for higher AR and shape factor (Fig. 6f,g,j) because MLCK is an immediate downstream effector of Ca^2+^^64,65^. We measured myosin light chain kinase (MLCK) activity using a CaM-sensor FRET probe^38,66^ (Fig. 6h,i,k) to obtain estimates of actin cytoskeletal dynamics associated with contractility. Elliptical cells showed a higher degree of MLCK FRET probe localization on actin filaments (Fig. 6k) and higher maximal activation compared to circular cells (Fig. 6h,i), indicating that the shape-induced increase in cytoplasmic Ca^2+^ signal propagates and is amplified downstream through MLCK activation to control contractility. This is consistent with the previous finding that higher aspect ratio VSMC are more contractile^29,67^ . This observation supports the relationship between cell shape, Ca^2+^ signaling and contractility which has been demonstrated by previous studies^29,30^. Here, we show that geometric factors affect the relationship between upstream signaling and downstream transcription factor activation, providing a physical link between two chemical pathways.

Increase in nuclear Ca^2+^ in elliptical cells is likely to impact the nucleo-cytoplasmic transport of NFAT, which exhibits a Ca^2+^/calcineurin dependent translocation and residence time in the nucleus^19,68^. We predict that increased AR leads to a modest increase in rate of increase of NFAT ratio over 500 s (Fig. 6m). We measured NFAT-GFP localization dynamics in live VSMCs in elliptical and circular 3D biochips in response to Gα_q_ activation through M_3_R stimulation (Fig. 6l,n,o,p). Elliptical cells exhibited greater NFAT-GFP nuclear localization compared to circular cells (Fig. 6n) and on average displayed higher maximal NFAT_nuc/cyto_ (Fig. 6o). We independently validated these differences in NFAT localization by immunofluorescent antibody-based visualization of NFAT1 (Fig. 6p, Supplementary Fig. 9a). At basal levels, NFAT_nuc/cyto_ were similar between circular and elliptical cells. However, elliptical cells displayed higher nuclear NFAT compared to circular cells at 30 min and 1 h after stimulation, consistent with live-cell NFAT-GFP translocation results.

To determine whether the effects of Ca^2+^ were general, we studied the localization of SRF in the nucleus. Ca^2+^ also triggers the nuclear localization of SRF through nuclear Ca^2+^/CaMKIV^20^ and actin dynamics^69,70^. Elliptical VSMCs show increased nuclear SRF compared to circular cells at both basal and stimulated levels (Supplementary Fig. 9b). In contrast there was no difference in the localization of myocardin, a transcription factor known to be unaffected by Ca^2+^ dynamics, between circular and elliptical cells (Supplementary Fig. 9c). Myocardin has been shown to be constitutively active^21,71^. Taken together, these results indicate that shape-induced modulation of Ca^2+^ signaling alters Ca^2+^ dependent physiological activities in both the cytoplasm and the nucleus.

### An integrated model of global cell shape and organelle location provides insight into curvature coupling of Ca^2+^ dynamics in VSMCs

We used spatial models constructed in both COMSOL and *Virtual Cell* to study the effects of cell curvature and PM–SR distance on calcium and downstream signaling dynamics. Our experimental observations find a qualitative association between cell aspect ratio and PM–SR distance (Fig. 4). Our model results predict that changing aspect ratio and PM–SR distance will modify signaling dynamics on both short- and long-timescales, and experimental observations validate these predictions (Figs. 5,6). We note that this qualitative trend of increased signaling AUC with increasing aspect ratio and increasing amplification factor was seen in both 2D and 3D models (Figs. 5,6 and Supp Fig. 13–15). Therefore, we conclude that cell shape and curvature regulates cell signaling in VSMCs, through changes in PM–SR distance. Briefly, it is important to note that currently there is some debate over the source of increased cytoplasmic calcium seen experimentally in VSMCs, with two competing hypotheses. The first explored in this paper is that cell shape and reduced PM–SR distances affects IP_3_ dynamics and triggers calcium release from the SR. The second is that a reduction in SR calcium triggers SOCE, Store Operated Ca^2+^ Entry, and extracellular Ca^2+^ influx through an interaction of STIM and ORAI channels^72,73^. In both of these cases, PM–SR distance would play a key role in regulating cytosolic Ca^2+^, whether through IP_3_ dynamics or STIM–ORAI interaction. However, it remains experimentally difficult to differentiate between these two methods of Ca^2+^ increase. Furthermore, in our model, we do not see extreme amounts of SR calcium depletion and SR calcium depletion is believed to be necessary for SOCE (see Fig. 3 in^74^). As we are operating in this regime and due to the continued controversy and lack of experimental evidence, we have omitted SOCE from the model.

## Discussion

**Figure 7:**
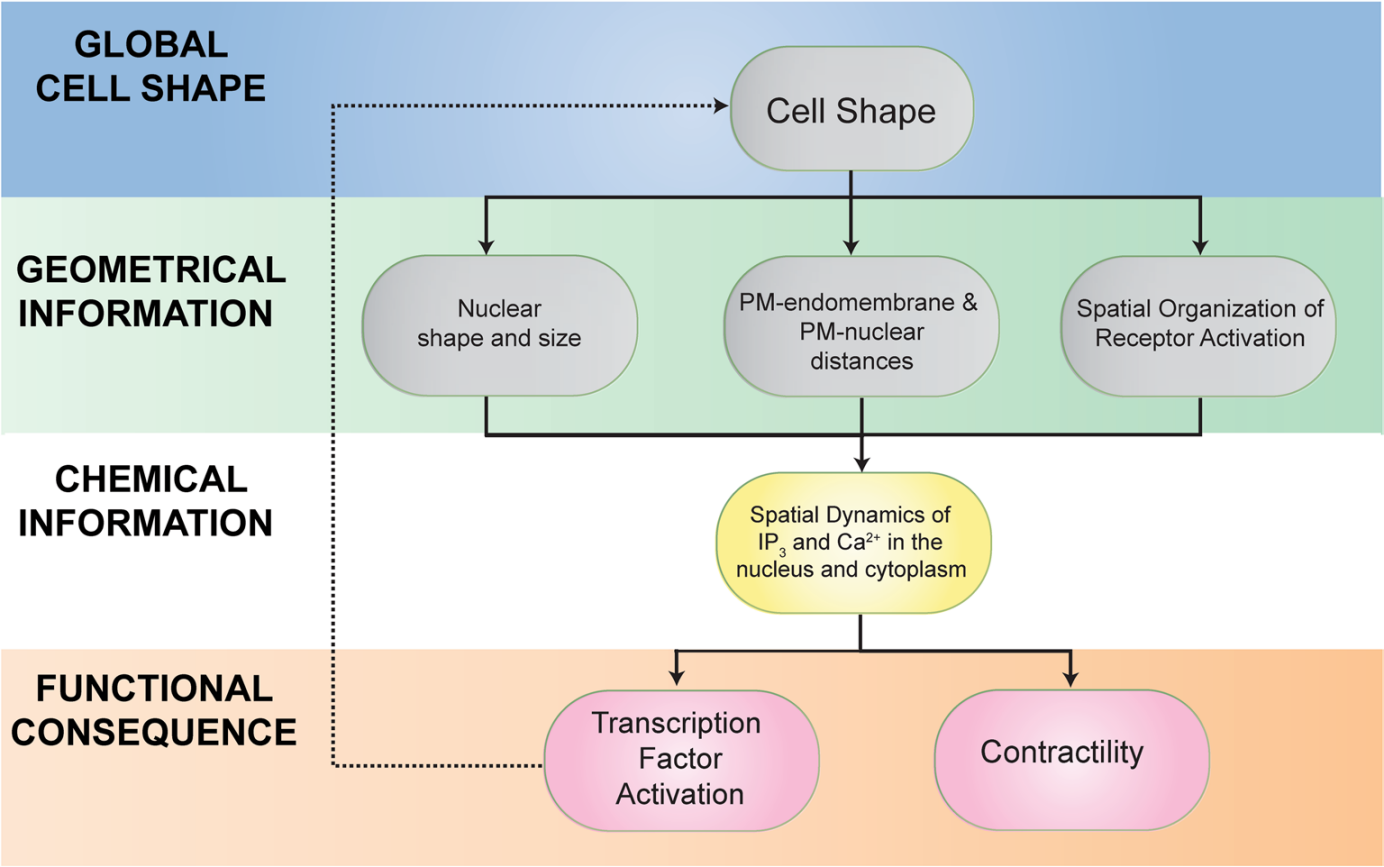
Systems approaches to controlling signaling through subcellular organization hierarchy. Physical determinants such as shape and geometrical information within the cell interact with organelle location and chemical information transfer to impact cellular function and gene expression, which in turn, feedback into cell shape. While each of these effects—shape, organelle location, and chemical information—may separately lead to small changes in signaling; collectively, these effects can lead to altered cellular function including transcription factor activation and contractility.

One of the key features of intracellular signal flow is the spatial organization of information propagation. This feature is used in multiple cell types to closely regulate the temporal dynamics of second messengers such as calcium and cAMP. For example, in cardiac and skeletal muscle cells, T-tubules, which are tubular invaginations that penetrate into the center of the cell are enriched in L-type calcium channels^75,76^. Mature dendritic spines in neurons have a specialized ER called the spine apparatus, which is thought to play a role in regulating calcium concentrations in spines^77^. Here, we bring together several seemingly independent effects of global cell shape to provide an integrated view of how curvature affects organelle location as well as distribution of receptors in the plane of the membrane to modulate signal transduction and thus affect cellular function (Fig. 7). Shape and biochemical signaling are coupled together in a feedback loop to maintain phenotype: cell shape integrates external mechanical and chemical signals on the plasma membrane^41^ while intracellular signaling cascades containing chemical information in the form of reaction kinetics, in turn regulate cell shape^78,79^. We propose that shape-dependent endomembrane and nuclear organization serve as the critical link that connect these two components in the feedback loop. This intricate, non-linear coupling of geometric and chemical information can potentially lead to signal amplification to control phenotype.

We used VSMC as a model system to unravel the complex relationship between global cell curvature, signaling, and endomembrane organization. During atherogenesis, disruption of local microenvironment in the medial layer of blood vessels causes VSMC to lose its native spindle shape, and subsequently lose contractile function^25,80^. It was not clear how loss of shape can lead to a decrease in contractile function. This study provides a mechanistic explanation for the functional role of shape in VSMC contractile function. Cell shape governs membrane curvature, which enables the emergence of systems level properties; cell elongation simultaneously concentrates plasma membrane receptors in the flatter regions of the membrane and reduces the distance between the PM–SR and SR-nucleus in the same region, hence effectively forming a diffusion-restricted domain where receptors, SR, and the nucleus become closer to each other, establishing high effective IP_3_ and Ca^2+^ concentration in the cell center. Given the slow diffusion coefficient of IP_3_ (≤ 10 µm^2^/s) which limits the range of action over which it can exert its signal^31^ and the dimensions of typical spindle-shaped VSMC (long axis ≥ 150 µm and short axis length of ≤ 10 µm), control of endomembrane organization by cell shape is a physical, non-genomic mechanism by which IP_3_/Ca^2+^ signals can be locally amplified in order to achieve high concentration of Ca^2+^ globally. These can further regulate contractility through amplification of downstream signaling pathways of MLCK and Ca^2+^-dependent gene expression in the nucleus. Although the effect of cell shape on upstream signaling events and global calcium are small, the progressive amplification during signal flow to physiological effectors through coupled reaction kinetics and spatial organelle organization can produce very different phenotypic effects. This relationship between small changes in levels of second messenger resulting in altered phenotypic response is not unique to calcium signaling pathways. Back in 1994, we had shown that small changes in cAMP levels by activated Gαs could inhibit transformation of NIH-3T3 fibroblasts by oncogenic Ras^81^.

While we have extensively explored the role of cell shape in signaling here and previously^3,4^, we have taken an experimental approach using engineered shapes to identify the effects of cell shape by itself. At a conceptual level this is like studying the biochemical activity of a purified protein without the influence of its regulators. In the case of our cell shape studies, features that we have not considered here, such as the forces exerted by the extracellular microenvironment on the cell, and vice versa, also play a critical role in transmitting geometric information^82,83^, trafficking^84^, and signal transduction^85^. Furthermore, the interaction between signaling and cytoskeletal remodeling can lead to changes in cell shape and local curvature^86,87^. Our observations of increasing anisotropy and robustness in expression of actin myofibrils, along with increased nuclear SRF localization with aspect ratio indicate that cytoskeletal signaling also contributes towards the contractile phenotype of VSMC. Cytoskeletal and Ca^2+^ signaling may act in concert in maintaining the differentiated phenotype of spindle-shaped VSMC. We have able to uncover unique aspects of signal flow regulation in cells based on geometry (cell shape) and chemical reaction cascades alone, and coupling these effects with the effects of extracellular microenvironment and the role of intracellular cytoskeletal interactions is a focus of future studies. We conclude that being at the right place at the right time is critical for information to flow from one cellular compartment to another and for short term signals like Ca^2+^ to have long lasting effects such as contractility and transcription factor activity.

## Supplementary information

Supplementary Information.

## Data Availability

Data supporting the findings of this study are available within the paper and its supplementary information.
