# Supplementary material for: Cell shape regulates subcellular organelle location to control early Ca2+ signal dynamics in vascular smooth muscle cells
Source: Sci Rep. 2020 Oct 20;10:17866. doi: 10.1038/s41598-020-74700-x (PMC7576209; doi:10.1038/s41598-020-74700-x)
Supplement: Supplementary file 1 — Supplementary Information. [file 41598_2020_74700_MOESM1_ESM.pdf]

SUPPLEMENTARY INFORMATION

Cell shape regulates subcellular organelle location to control early Ca^{2+} signal dynamics in Vascular Smooth Muscle Cells

Authors: R. C. Calizo[1,$], M. K. Bell[4,$], A. Ron[2], M. Hu[2], S. Bhattacharya[2,5], N. J. Wong[5], W.G.M. Janssen[1], G. Perumal[6], P. Pederson[6], S. Scarlata[3], J. Hone[2], E. U. Azeloglu[1,5], P. Rangamani[4]*, and R. Iyengar[1]*

Affiliations:

[1]Department of Pharmacological Sciences and, Institutefor Systems Biomedicine, Icahn School of Medicine at Mount Sinai, New York, NY 10029

[2]Department of Mechanical Engineering, Columbia University, New York, NY 10027.

[3]Department of Chemistry and Biochemistry, Worcester Polytechnic Institute, Worcester, MA 01609.

[4]Department of Mechanical and Aerospace Engineering, University of California San Diego, La Jolla, CA 92093.

[5]Division of Nephrology, Department of Medicine, Icahn School of Medicine at Mount Sinai, New York, NY 10029

[6] Carl Zeiss Microscopy LLC, White Plains, NY 10601

[$]Contributed equally to this work.

*To whom correspondence should be addressed:

Ravi Iyengar, Ph.D.
Department of Pharmacological Sciences
Icahn School of Medicine at Mount Sinai
One Gustave L. Levy Place, Box 1215
New York NY 10029
Phone: 212-659-1707
Fax: 212-831-0114
e-mail: ravi.iyengar@mssm.edu

or

Padmini Rangamani, Ph.D.
Department of Mechanical and Aerospace Engineering
University of California San Diego
La Jolla, CA 92093
Phone: 858-534-4734
e-mail: prangamani@ucsd.edu

NUMERICAL SOLUTIONS FOR REACTION DIFFUSION EQUATIONS WITH ROBIN BOUNDARY CONDITIONS.

We investigate the dynamics of a molecule C_A, which is formed at the PM, freely diffuses in the cytoplasm where it can be degraded, and is consumed at the SR membrane. This phenomenon can be represented by the following reaction-diffusion equation with the accompanying boundary conditions.

$$\frac{\partial [C_A]}{\partial t} = D_{C_A} \nabla^2 [C_A] - k_{deg}[C_A]$$

$$D(\mathbf{n} \cdot \nabla [C_A])|_{PM} = k_{on}[C_A]$$

$$D(\mathbf{n} \cdot \nabla [C_A])|_{SR} = -k_{off}[C_A]$$

One of the features of this system of equations is that the boundary conditions at both ends are time-dependent and belong to a class of boundary conditions known as Robin boundary conditions [1]. Furthermore, the boundary conditions depend on the local shape, since the surface normal will change as a function of curvature[2]. If the boundary conditions at both membranes were constant values of the concentration of A, the solution to the partial differential equation (Eq. 1) is a linear distribution from one boundary to the other[1]. However, in the current case, where both boundary conditions have Robin boundary conditions, the solution to these equations depends *both* on the diffusion distance and the reaction rates. The Robin boundary conditions that accompany the reaction-diffusion equation are of the mixed-type and are time dependent. Therefore, writing analytical solutions for them is challenging. Therefore, we use numerical methods to solve this equation. We use finite-element methods to solve the equations in the commercially available software COMSOL Multiphysics®.

Briefly, we used the General Form PDE interface to model the partial differential equations and the flux boundary conditions implemented using the Flux mode with the source term set to the reaction rate. We used six different geometries to test the role of curvature and PM-SR distance. The mesh size was set to 'Extremely Fine' and the tolerance was set to relative tolerance was set to 0.01. For the toy model displayed in Fig. 1b, we used parameter values of k_{deg} = 0.5 [1/s], k_{on} = k_{off} = 0.75 [μm/s)], D_{Ca} = 1 [μm^2/s], and Ca_{IC} = 5 [μM]. Area was conserved across all shapes. Rectangles had heights and widths of 150 nm and 500 nm, 100 nm and 750 nm, and 50 nm and 1500 nm. The circle section had an inner radius of 500 nm, a width of 150 nm, and a sector angle of 50 degrees. The constant elliptical section had a width of 100 nm and sector angle of 73 degrees. The tapering elliptical section had a starting width of 132 nm, an ending height of 25 nm, and a sector angle of 90 degrees. The plot in Fig. 1b is at 50 ms and has a range of values from ~4.7 to 5.9 μM. This toy model served to demonstrate the intricate relationship between curvature and PM-SR distance.

For a rectangle and a circle, solving the PDE with the boundary conditions shown above amounts to solving a 1-dimensional problem (either in the z-direction or in the radial direction). As a result, a gradient is observed only in one direction. For an elliptical cross section (Fig. 1A), a triangle (Supplementary Fig. 1A) and a trapezium (Supplementary Fig. 1B), the geometric variation is in two-dimensions. Therefore, the spatio-temporal dynamics of C_A is different in

these patterns. Furthermore, in an ellipse, the curvature is varying along the membrane, and therefore, the diffusive flux also varies. This is because the diffusive flux is dependent on the local normal and curvature captures the rate of change of the normal along the curve[2].

Dimensional analysis

Using the PM-SR distance 'L' as a characteristic length scale and k_{deg} as the characteristic time scale, we non-dimensionalized the partial differential equation for C_A to obtain a dimensionless number $(k_{deg}L^2)/D$ which is the Damkohler number[1]. When the Damkohler number is much greater than 1, then the system is diffusion-dominated and when the Damkohler number is much less than 1, it is reaction-dominated. In cellular environments, various protein interactions and obstacles can lead to diffusion-trapping, which slows diffusion to an effective diffusion given by $D_{eff} = \frac{D}{1+\gamma_{geo}(1+\beta_{kin})}$, where γ_{geo} represents the geometric effects of traps and β_{kin} represents kinetics of binding at traps if applicable [3,4]. Replacing D in the Damkohler number with this D_{eff} captures the effects of geometric and kinetic barriers to diffusion that can alter signaling dynamics.

IP_3 model implementation in COMSOL

The dynamics of IP_3 were modeled using the reaction network model presented in Cooling et al.[5] and implemented in COMSOL using the protocol presented in Vollmer et al.[6]. We modeled multiple compartments – the extracellular space, PM, SR, SR membrane, the nucleus and the nuclear membrane. The various reactions can be found in Supplemental Table 1 from R1-R16. The Robin boundary conditions were implemented using the Flux boundary condition node for the volume and the general boundary form PDE node for the membranes. The mesh size was set to 'Extremely fine' such that the minimum element size is 0.1 nm and relative tolerance was set to 0.01. These values of mesh size and tolerance were chosen such that smaller values of tolerance or mesh refinement didn't alter the numerical results.

INTEGRATIVE WHOLE-CELL REACTION-DIFFUSION MODEL

To understand how global cell shape can modulate the dynamics of IP_3 and calcium signaling, we developed mathematical models which analyze the relationships between the binding of extracellular ligand to GPCR, G-protein activation and cycling, PLCβ activation, IP_3 activation of IP_3R in the SR, intracellular calcium dynamics, MLCK activation, and NFAT dynamics. Reactions were initially implemented as a system of ordinary differential equation in the *Virtual Cell* environment (http://www.nrcam.uchc.edu/)[7], and then modeled as a system of partial differential equations (PDEs) in two-dimensions, and then implemented in three dimensions. The complete list of model equations, parameters, units and references of the kinetic parameters are listed in Table 1 and the complete list of initial conditions and diffusion coefficients are listed in Table 2.

GPCR Signaling-PLCβ cycling

We adapted the GPCR signaling modules described by Cooling et al[5] and Eungdamrong et al[8] with modifications. Reactions R1-R6 represent GPCR-Ligand-G protein interactions on the plasma membrane. The reactions are modeled as kinetic fluxes following the mass action and [9]Michaelis-Menten kinetics (see Bhalla and Iyengar). A ligand stimulus of 10 μM was applied from 100s to 150s. The binding of extracellular ligand to cell-surface receptors are represented

by reactions R1 and R4. Binding of the ligand to muscarinic acetylcholine receptors causes a conformational change on the $G\alpha_q$ subunit of the heterotrimeric G-protein, causing replacement of GDP with GTP (R5) and dissociation of the $G\alpha$ subunit from the heterotrimeric G-protein. The binding of activated $G\alpha_q$ to the enzyme PLCβ are represented by R8 and R10. $G\alpha_q$-GTP is hydrolyzed to $G\alpha_q$-GDP by autocatalysis represented by R7. Reactions R14-R16 represent IP3 dynamics, including its production at the plasma membrane, membrane diffusion and degradation at the cytosol. R14 and R15 shows the hydrolysis of PIP_2 to IP_3 by PLCβ-Ca^{2+} and PLCβ-Ca^{2+}-$G\alpha_q$(GTP) respectively. The degradation of IP_3, which involves the dephosphorylation and phosphorylation of IP3 to form inositol biphosphate and inositol tetraphosphate are represented by R16. The metabolic recycling of IP_3 from these products is not considered. PLCβ inactivation is represented by R13.

IP_3/Calcium Dynamics

In vascular smooth muscle cells, agonist-induced calcium release from the SR/ER is the main source of intracellular Ca^{2+} transient. The opening of the inositol triphosphate receptor channels (IP_3R) from the ER membrane, leads to an increase in cytoplasmic calcium. We used the simplified version of channel kinetics [10,11] by De Young and Keizer [12] which is represented by R17. Calcium leak from the ER to the cytosol is represented by R20. Calcium is pumped back to the ER by the sarcoplasmic reticulum calcium ATPase (SERCA) pump, represented by R21. Calcium is also pumped into the nucleus through the NPC, represented by R41. We also model downstream signaling dependent on cytosolic calcium including activation of Calmodulin (CaM), Calcineurin (CaN), CaMKII, MLCK, and NFAT (R22-40).

Geometries

The spatial geometries in *Virtual Cell* of the cell and organelles were depicted as idealized geometries represented as a series of concentric ellipsoids that represent the whole cell, SR and the nucleus. We assumed that cells complying with shapes of increasing aspect ratios (AR) conserve the cell volume and increase the plasma membrane surface area with increasing AR. Hence, whole cell, cytoplasmic and SR areas were kept constant with increasing aspect ratios for 2D simulations, while whole cell, cytoplasmic and SR volumes were kept constant with increasing aspect ratios for 3D simulations. The cellular geometries were approximated from experimentally observed VSMC confined from AR 1:1 to AR 1:8 (Figure 3a), whereby the SR is closer to the PM in the perinuclear region compared to the cell tips. Furthermore, as the aspect ratio increased, both PM-SR and PM-nuclear distances decreased in the minor axis and increased in the major axis of the ellipse. Nuclear shapes were based on experimentally observed geometries (Fig. 3c-e). Supplementary Fig. 7 show the resulting 3D and 2D geometries, respectively. To solve the PDEs, geometries were discretized into 0.5 μm x 0.5 μm spatial steps. The systems of equations were solved within the Virtual Cell framework using fully-implicit, finite volume, with variable time step solver, with a maximum time step of 0.1 s and an output interval of 5.0 second.

We note that different geometric assumptions were made in simulations for *Virtual Cell* versus COMSOL. COMSOL simulations included disc geometries for the SR that layered on top of each other next to the PM. *Virtual Cell* involved concentric ellipsoids where the SR was modeled as sectors of ellipsoids based on experimental imaging. We note however that real SR structures can be highly complex with layers and folding, which cannot yet be captured fully with either framework (see [28]). The *Virtual Cell* framework was not able to resolve small distances between

PM and SR such as 50-100 nm. This is why we used COMSOL for studying the small length scale effects. Therefore, the COMSOL model was used to implement a simplified biochemical study, but with additional geometric complexity through the layered SR disc stacks.

Amplification factors for the SR

Idealized geometries were used for the cell body and internal organelles. However, it is well-known that the SR in particular has complex structures with large surface area. To account for this large area and to investigate its role in regulating the IP_3-mediated calcium flux at the ER membrane, an amplification factor was included in the membrane flux of IP_3 activated calcium release from the SR, R17, and effectively scales the magnitude of R17. By varying this parameter, we investigated the effect of different surface areas of the SR in the model, without explicitly simulating the model equations in these complex geometries.

Parametric Sensitivity Analysis

A parametric sensitivity analysis for cytoplasmic calcium and activated MLCK was carried out with respect to all model parameters and initial conditions (Supplementary Figs. 18-21). The analysis was carried out in the software COPASI (www.copasi.org) where the model was implemented as a system of ordinary differential equations.

Area under the curve Analysis

We used several methods to convey our spatiotemporal signaling results including temporal plots, spatial images, and Area under the Curve (AUC) analysis. AUC is a commonly used measurement of signaling over time. In computational spatial models, it is typically computed by integrating concentration over the whole geometry at each time point, then integrating the temporal plot of moles (or molecules) over time, producing an AUC with units of moles*sec for example. Experimental results usually integrate temporal concentration over time, producing an AUC with units of μM*sec. As AUC is an additive measure, that is it accumulates the signal over time, AUC will always be increasing or constant.

Supplementary Table 1. Model reactions and parameter values

Reaction	Description	Model equation	Parameter	Value	Units	Reference
		Membrane Receptor Reactions				
R1	Ligand + GPCR <-> Ligand-GPCR<br>*Ligand binding*	Kf_{r1}[Ligand] [GPCR] -<br>Kr_{r1}[Ligand-GPCR]	Kf_{r1}<br>Kr_{r1}	3.00×10^{-4}<br>4.50×10^{-7}	$s^{-1}\cdot\mu M^{-1}$<br>s^{-1}	[5,13]
R2	Ligand-GPCR + Gαq-GDP <-> Ligand-GPCR- Gαq-GDP<br>*GPCR cycling*	Kf_{r2}[Gαq-GDP][Ligand-GPCR] –<br>Kr_{r2}[Ligand-GPCR- Gαq-GDP]	Kf_{r2}<br>Kr_{r2}	1.00×10^{-0}<br>1.00×10^{-3}	$\mu m^2\cdot s^{-1}\cdot molecules^{-1}$<br>$s^{-1}$	[5,13]
R3	GPCR + Gαq-GDP <-> GPCR-Gαq-GDP<br>*GPCR cycling*	Kf_{r3}[Gαq-GDP][GPCR]-<br>Kr_{r3}*[GPCR-* Gαq-GDP *]*	Kf_{r3}<br>Kr_{r3}	2.75×10^{-4}<br>7.54×10^{0}	$\mu m^2\cdot s^{-1}\cdot molecules^{-1}$<br>$s^{-1}$	[5,13]
R4	GPCR-Gαq-GDP + Ligand <-> Ligand-GPCR- Gαq-GDP<br>*Ligand binding*	Kf_{r4}*[GPCR-* Gαq-GDP *][Ligand]* -<br>Kr_{r4}*[Ligand-GPCR-* Gαq-GDP*]*	Kf_{r4}<br>Kr_{r4}	6.02×10^{-1}<br>9.03×10^{-4}	$s^{-1}\cdot\mu M^{-1}$<br>s^{-1}	[5,13]
R5	Ligand-GPCR-Gαq-GDP <-> Ligand-GPCR+Gαq<br>*Gαq activation*	Kf_{r5}*[Ligand-GPCR-*Gαq-GDP*]* –<br>Kr_{r5}*[Gαq-GTP][Ligand-GPCR]*	Kf_{r5}<br>Kr_{r5}	2.22×10^{1}<br>0.00×10^{0}	s^{-1}<br>$\mu m^2\cdot s^{-1}\cdot molecules^{-1}$	[5,13]
R6	Ligand-GPCR-Gαq-GDP <-> Ligand-GPCR- Gαq-GDP -P<br>*GPCR phosphorylation*	Kf_{r6}[Ligand- GPCR-Gαq-GDP] -<br>Kr_{r6}[Ligand-GPCR-Gαq-GDP-P]	Kf_{r6}<br>Kr_{r6}	6.22×10^{-2}<br>0.00×10^{0}	s^{-1}<br>s^{-1}	[5,13]
R7	Gαq-GTP <-> Gαq-GDP<br>*Gαq deactivation*	Kf_{r7}[Gαq-GTP] - Kr_{r7}*[Gαq-GDP]*	Kf_{r7}<br>Kr_{r7}	1.50×10^{-1}<br>0.00×10^{0}	s^{-1}<br>s^{-1}	[5,13]
R8	Gαq-GTP + PLCβ <-> PLCβ-Gαq-GTP<br>*Gαq binding to PLCβ*	Kf_{r8}[PLCβ][Gαq-GTP] - Kr_{r8}[PLCβ-Gαq-GTP]	Kf_{r8}<br>Kr_{r8}	4.20×10^{-2}<br>1.00×10^{0}	$\mu m^2\cdot s^{-1}\cdot molecules^{-1}$<br>$s^{-1}$	[5,13]
R9	Ca_{cyto} + PLCβ <-> PLCβ-Ca<br>*Calcium binding to PLCβ*	Kf_{r9}[Ca_{cyto}][PLCβ]- Kr_{r9}[PLCβ-Ca]	Kf_{r9}<br>Kr_{r9}	4.18×10^{-3}<br>1.67×10^{-2}	$s^{-1}\cdot\mu M^{-1}$<br>s^{-1}	[5,13] modified
R10	Gαq-GTP+PLCβ-Ca <-> PLCβ-Ca-Gαq-GTP<br>*PLCβ activation by Gαq*	Kf_{r10} [Gαq-GTP][PLCβ-Ca] - Kr_{r10}[PLCβ-Ca-Gαq-GTP]	Kf_{r10}<br>Kr_{r10}	4.20×10^{-2}<br>1.00×10^{0}	$\mu m^2\cdot s^{-1}\cdot molecules^{-1}$<br>$s^{-1}$	[5,13]
R11	Ca_{cyto} + PLCβ-Gαq-GTP <-> PLCβ-Ca-Gαq-GTP<br>*PLCβ- Gαq activation by Ca^{2+}*	Kf_{r11}[Ca_{cyto}][PLCβ-Gαq-GTP] - Kr_{r11}[PLCβ-Ca-Gαq-GTP]	Kf_{r11}<br>Kr_{r11}	3.34×10^{-2}<br>3.34×10^{-3}	$s^{-1}\cdot\mu M^{-1}$<br>s^{-1}	[5,13]
R12	PLCβ-Ca-Gαq-GTP <-> Gαq-GDP + PLCβ-Ca<br>*PLCβ dissociation from Gαq*	Kf_{r12}*[*PLCβ-Ca-Gαq-GTP*]* –<br>Kr_{r12} [Gαq-GDP][PLCβ-Ca]	Kf_{r12}<br>Kr_{r12}	6.00×10^{0}<br>0.00×10^{0}	s^{-1}<br>$\mu m^2\cdot s^{-1}\cdot molecules^{-1}$	[5,13]
R13	PLCβ-Gαq-GTP <-> Gαq-GDP + PLCβ<br>*PLCβ deactivation*	Kf_{r13}*[*PLCβ-Gαq-GTP*]* –<br>Kr_{r13} [Gαq-GDP][PLCβ]	Kf_{r13}<br>Kr_{r13}	6.00×10^{0}<br>0.00×10^{0}	s^{-1}<br>$\mu m^2\cdot s^{-1}\cdot molecules^{-1}$	[5,13]

		PIP₂/IP₃ Metabolism				
R14	PIP_2 + PLCβ-Ca-Gαq-GTP <-> IP_3 + DAG<br>*PIP_2 hydrolysis by PLCβ*	$\frac{Kcat_{r14}[PLC\beta - Ca - GTP][PIP_2]}{Km_{r14} + [PIP_2]}$	$Kcat_{r14}$<br>Km_{r14}	1.00×10^{1}<br>6.53×10^{2}	s^{-1}<br>molecules· μm^{-2}	[5,13],converte to appropriat units
R15	PIP_2 + PLCβ-Ca <-> IP_3 + DAG<br>*PIP_2 hydrolysis by PLCβ*	$\frac{Kcat_{r15}[PLC\beta - Ca][PIP_2]}{Km_{r15} + [PIP_2]}$	$Kcat_{r14}$<br>Km_{r14}	3.33×10^{-1}<br>2.587×10^{3}	s^{-1}<br>molecules· μm^{-2}	[5,13], modifiec converted tc appropriate units
R16	IP_3 <-> IP_3*<br>*IP_3 degradation*	$Kf_{r16}([IP_3]- [IP_3]_0)$	Kf_{r16}<br>IP_{30}	1.25×10^{-1}<br>1.50×10^{-2}	s^{-1}<br>μM	5
		Calcium Signaling				
R17	Ca_{cyt} <->Ca_{ER}<br>*IP3R channel open probability*	$-10 * ER_{erMembrane} * shapeER * (RactCa + Ract) * (Ca_{ER} - Ca_{cyt}) * (\frac{([IP3] * Ract * Rinh)}{[IP3 + dI](RactCa + Ract)(RinhCa + Rinh)})^3 singleChanFlux$	*singlechanFlux*<br>*dI*	1.45×10^{0}<br>2.50×10^{-1}	$\frac{\mu m^5}{molecules^2 s}$<br>μM	8
R18	*Ract + Ca_{cyto} <-> RactCa*	$Kfr18*Ract*[Ca_{cyt}]-Kr_{r18}*RactCa$	Kf_{r18}<br>Kr_{r18}	1.00×10^{3}<br>2.00×10^{2}	$\mu M^{-1}s^{-1}$<br>s^{-1}	8,11,14
R19	*Rinh + Ca_{cyto} <-> RinhCa*	$K_{onr19}*Rinh*Ca_{cyt} - K_{offr19}*RinhCa$	K_{onr19}<br>K_{offr19}	2.10×10^{0}<br>2.31×10^{-1}	$\mu M^{-1}s^{-1}$<br>s^{-1}	8,11,14
R20	Ca_{ER} -> Ca_{cyto}<br>*Calcium leak from the ER*	$-ER_{erMembrane}(Ca_{ER} - Ca_{cyt})*vL$	*vL*	3.00×10^{-7}	$\frac{\mu m^3}{molecules \cdot s}$	10
R21	Ca_{cyt} <-> Ca_{ER}<br>*SERCA channel*	$\frac{ER_{erMembrane}[SERCA] * vP * [Ca_{cyto}]^2}{(kP^2 + [Ca_{cyto}]^2)}$	*kP*<br>*vP*	2.30×10^{0}<br>2.92×10^{-2}	μM<br>$\frac{\mu M \cdot \mu m^5}{molecules^2 \cdot s}$	10
R22	$2Ca_{cyt}$ + CaM <-> $Ca_{cyt}{}^{C}{}_2$/CaM<br>*Calcium binding CaM C-term*	$k_1[CaM][Ca_{cyto}]^2 - k_{-1}[Ca_2^C CaM]$	k_1<br>k_{-1}	2.8×10^{0}<br>1.5×10^{0}	$\mu M^{-2}s^{-1}$<br>s^{-1}	15
R23	$2Ca_{cyt}$ + CaM <-> $Ca_{cyt}{}^{N}{}_2$/CaM<br>*Calcium binding CaM N-term*	$k_2[CaM][Ca_{cyto}]^2 - k_{-2}[Ca_2^N CaM]$	k_2<br>k_{-2}	1.80×10^{2}<br>4.80×10^{2}	$\mu M^{-2}s^{-1}$<br>s^{-1}	15
R24	$2Ca_{cyt}$ + $Ca_{cyt}{}^{C}{}_2$/CaM <-> Ca_{cyt4}/CaM<br>*Calcium binding $Ca^C{}_2$ CaM*	$k_2[Ca_2^C CaM][Ca_{cyto}]^2 - k_{-2}[Ca_4 CaM]$	k_2<br>k_{-2}	1.8×10^{2}<br>4.8×10^{2}	$\mu M^{-2}s^{-1}$<br>s^{-1}	15
R25	$2Ca_{cyt}$ + $Ca_{cyt}{}^{N}{}_2$/CaM <-> Ca_{cyt4}/CaM<br>*Calcium binding $Ca^N{}_2$ CaM*	$k_1[Ca_2^N CaM][Ca_{cyto}]^2 - k_{-1}[Ca_4 CaM]$	k_1<br>k_{-1}	2.8×10^{0}<br>1.5×10^{0}	$\mu M^{-2}s^{-1}$<br>s^{-1}	15

R26	MLCK + $Ca_{cyt}{}^{C}{}_{2}$/CaM <-> $Ca_{cyt}{}^{C}{}_{2}$/CaM/MLCK *Calcium binding $Ca^{C}{}_{2}$ CaM*	$k_3[Ca_2^C CaM][MLCK] - k_{-3}[Ca_2^C CaM - MLCK]$	k_3<br>k_{-3}	2.8×10^1<br>3×10^0	$\mu M^{-1}s^{-1}$<br>s^{-1}	15
R27	MLCK + $Ca_{cyt}{}^{N}{}_{2}$/CaM <-> $Ca_{cyt}{}^{N}{}_{2}$/CaM/MLCK *MLCK binding $Ca^{N}{}_{2}$ CaM*	$k_3[Ca_2^N CaM][MLCK] - k_{-3}[Ca_2^N CaM - MLCK]$	k_3<br>k_{-3}	2.8×10^1<br>3×10^0	$\mu M^{-1}s^{-1}$<br>s^{-1}	15
R28	$2Ca_{cyt}$ + $Ca_{cyt}{}^{C}{}_{2}$/CaM/MLCK <-> MLCKact *Calcium binding $Ca^{C}{}_{2}$ CaM-MLCK*	$k_4[Ca_2^C CaM - MLCK][Ca_{cyto}]^2 - k_{-4}[MLCK_{act}]$	k_4<br>k_{-4}	1.8×10^1<br>2.8×10^0	$\mu M^{-2}s^{-1}$<br>s^{-1}	15
R29	$2Ca_{cyt}$ + $Ca_{cyt}{}^{N}{}_{2}$/CaM/MLCK <-> MLCKact *Calcium binding $Ca^{N}{}_{2}$ CaM-MLCK*	$k_5[Ca_2^N CaM - MLCK][Ca_{cyto}]^2 - k_{-5}[MLCK_{act}]$	k_5<br>k_{-5}	2.0×10^{-1}<br>2.5×10^{-1}	$\mu M^{-2}s^{-1}$<br>s^{-1}	15
R30	Ca_{cyt4}/CaM + MLCK <-> MLCKact *Ca_4CaM binding MLCK*	$k_6[Ca_4CaM][MLCK] - k_{-6}[MLCK_{act}]$	k_6<br>k_{-6}	2.8×10^1<br>3.0×10^{-2}	$\mu M^{-1}s^{-1}$<br>s^{-1}	15
R31	Ca_{cyt4}/CaM + CaN <-> Ca_{cyt4}/CaM/CaN Ca_{cyt4}/CaM *binding CaN*	$k_8[Ca_4CaM][CaN] - k_{-8}[Ca_4CaM - CaN]$	k_8<br>k_{-8}	2.6×10^1<br>1.2×10^{-3}	$\mu M^{-1}s^{-1}$<br>s^{-1}	16
R32	Ca_{cyt4}/CaM/CaN +NFAT-p <-> Ca_{cyt4}/CaM/CaN + NFAT *CaN complex activating NFAT*	$\frac{k_{cat9}[NFATp][Ca_4CaM - CaN]}{k_{m9} + [NFATp]}$	k_{cat9}<br>k_{m9}	3.16×10^{-1}<br>7.07×10^{-1}	s^{-1}<br>μM	[17], rates [1[illegible]
R33	Ca_{cyt4}/CaM +CaMKII <-> Ca_{cyt4}/CaM + CaMKIIp Ca_{cyt4}/CaM *activating CaMKII*	$\frac{k_{cat10}[Ca_4CaM]^4[CaMKII]}{k_{m10}{}^4 + [Ca_4CaM]^4}$	k_{cat10}<br>k_{m10}	1.2×10^2<br>4×10^0	s^{-1}<br>μM	19
R34	CaMKIIp +CaMKII <-> CaMKIIp *CaMKII autophosphorylation*	$\frac{k_{cat11}[CaMKIIp][CaMKII]}{k_{m11} + [CaMKII]}$	k_{cat11}<br>k_{m11}	1×10^0<br>1.0×10^1	s^{-1}<br>μM	19
R35	CaMKIIp + Ca_{cyt4}/CaM/CaN <-> CaMKII + Ca_{cyt4}/CaM/CaN *CaMKIIp deactivation by CaN*	$\frac{k_{cat12}[Ca_4CaM - CaN][CaMKIIp]}{k_{m12} + [CaMKIIp]}$	k_{cat12}<br>k_{m12}	1.5×10^1<br>3×10^0	s^{-1}<br>μM	19
R36	CaMKIIp + MLCK <-> CaMKIIp + MLCKact *CaMKIIp deactivation by CaN*	$\frac{k_{cat13}[CaMKIIp][MLCK]}{k_{m13} + [MLCK]}$	k_{cat13}<br>k_{m13}	1.8×10^0<br>2.47×10^0	s^{-1}<br>μM	19
R37	Ca_{cyt4}/CaM/CaN <-> CaN_{nuc} *CaN transport to the nucleus*	$k_{14}[Ca_4CaM - CaN] - k_{-14}[CaNnuc]$	k_{14}<br>k_{-14}	1.9×10^{-3}<br>9.2×10^{-4}	s^{-1}<br>s^{-1}	18
R38	NFATpnuc <-> NFATp *NFATp transport from the nucleus*	$k_{15}[NFATpnuc]$	k_{15}	9.6×10^{-4}	s^{-1}	18

R39	NFATpnuc <-> NFATnuc<br>*NFAT deactivation by CaN*	$\frac{k_{cat16}[CaNnuc][NFATpnuc]}{k_{m16} + [NFATpnuc]}$	$kcat_{16}$<br>km_{16}	7.07 x 10^{-1}<br>3.16 x 10^{-1}	s^{-1}<br>μM	18
R40	NFAT <-> NFATnuc<br>*NFAT transport to the nucleus*	$k_{17}[NFAT]$	k_{17}	1.54 x 10^{-3}	s^{-1}	18
R41	Ca_{cyt} -> Ca_{nuc}<br>*Calcium transport to the nucleus*	$k_{18}([Ca_{cyto}] - [Ca_{nuc}])[NPC]$	k_{18}	1.66 x 10^{-2}	$\frac{\mu m^3}{molecules\ s}$	This worl
R42	Ca_{nuc} -> Ca_{nuc_deg}<br>*Calcium decay in the nucleus*	$k_f[Ca_{nuc}]$	k_f	5.00 x 10^{-3}	s^{-1}	This worl

Supplementary Table 2. Initial Concentration and Diffusion coefficient of reactants.

Species	Initial Concentration (µM)	Diffusion Coefficient ($\mu m^2/s$)	References
Calcium	0.1 µM	220	Knot, Cooling [5,20]
IP3	0.015 µM	10	Cooling, Dickinson[5,21]
Ligand	10 µM	-	Agonist added
Calcium_nuc	0.122 µM	220	Value obtained after equilibration
Calcium_ER	400 µM	220	Fink[11,14]
Receptor	12 μm^{-2} *varied in spatial	0.1	Cooling, Bers[5,22]
L_R_GD	0 μm^{-2}	0.1	Unstimulated state
R_GD	1.07 μm^{-2}	0.1	Cooling, Bers[5,22]
GT	0 μm^{-2}	0.1	Unstimulated state
GD	10000 μm^{-2}	0.1	Cooling, Lukas[5,13]
PLC	90.9 μm^{-2}	0.1	Cooling, Lukas[5,13]
PLC_GT	0 μm^{-2}	0.1	Unstimulated state
PIP2	4000 μm^{-2}	2.5	Cooling, Xu, Golebiewska[5,23,24]
DAG	0.015 µM	237	This work
LR	0 μm^{-2}	0.1	Unstimulated state
PLC_Ca	9.09 μm^{-2}	0.1	Cooling, Lukas[5,13]
PLC_Ca_GT	0 μm^{-2}	0.1	Unstimulated state
L_R_GD_P	0 μm^{-2}	0.1	Unstimulated state
Rinh	7.7825 μm^{-2}	0.1	Fink, Eungdamrong[8,11,14]
Ract	9.056 μm^{-2}	0.1	Fink, Eungdamrong[8,11,14]
RinhCa	3.5375 μm^{-2}	0.1	Fink, Eungdamrong[8,11,14]
RactCa	2.264 μm^{-2}	0.1	Fink, Eungdamrong[8,11,14]
SERCA	45 μm^{-2}	0.1	Fink, Eungdamrong[8,11,14]

ER_erMembrane	2 μm^{-2}	--	Fink, Eungdamrong[8,11,14]
CaM	6 μM	10	[17,25]
CaN	9.853×10^{-1} μM	10	[18], this work
CaN*	1.413×10^{-2} μM	10	[18], this work
CaN*_nuc	2.918×10^{-2} μM	10	[18], this work
Ca_2CaM (Ca2nCaM+Ca2cCaM)	7.012×10^{-3} μM	10	[18], [25]
Ca_4CaM	4.05×10^{-7} μM	10	[18], [25]
CaMKII	1 μM	7	This work, [25]
Ca2CaMMLCK	0 μM	0.05	[26]
MLCK	5 μM	0.05	[15,26]
NFATp	1.617×10^{-2} μM	0.1	[18], based on [27]
NFAT	1.17×10^{-4} μM	0.1	[18], based on [27]
NFATnuc	3.542×10^{-2} μM	0.1	[18], [27]
NFATpnuc	1.88×10^{-4} μM	0.1	[18], [27]
NPC	1 μm^{-2}	1	This work

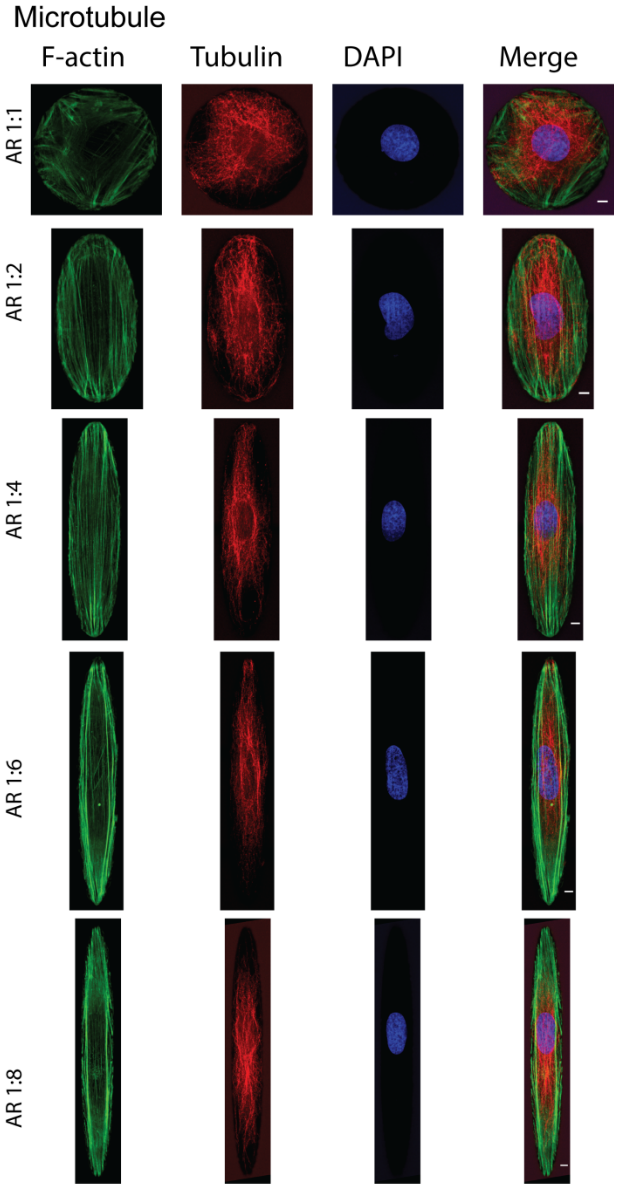

Supplementary Figure 1. Microtubule organization in VSMC with AR 1:1 to AR 1:8. Shown are staining of F-actin (green), alpha-tubulin (red) and DAPI in VSMC complying with AR 1:8. Scale bars shown in the merged channel are 10 µm.

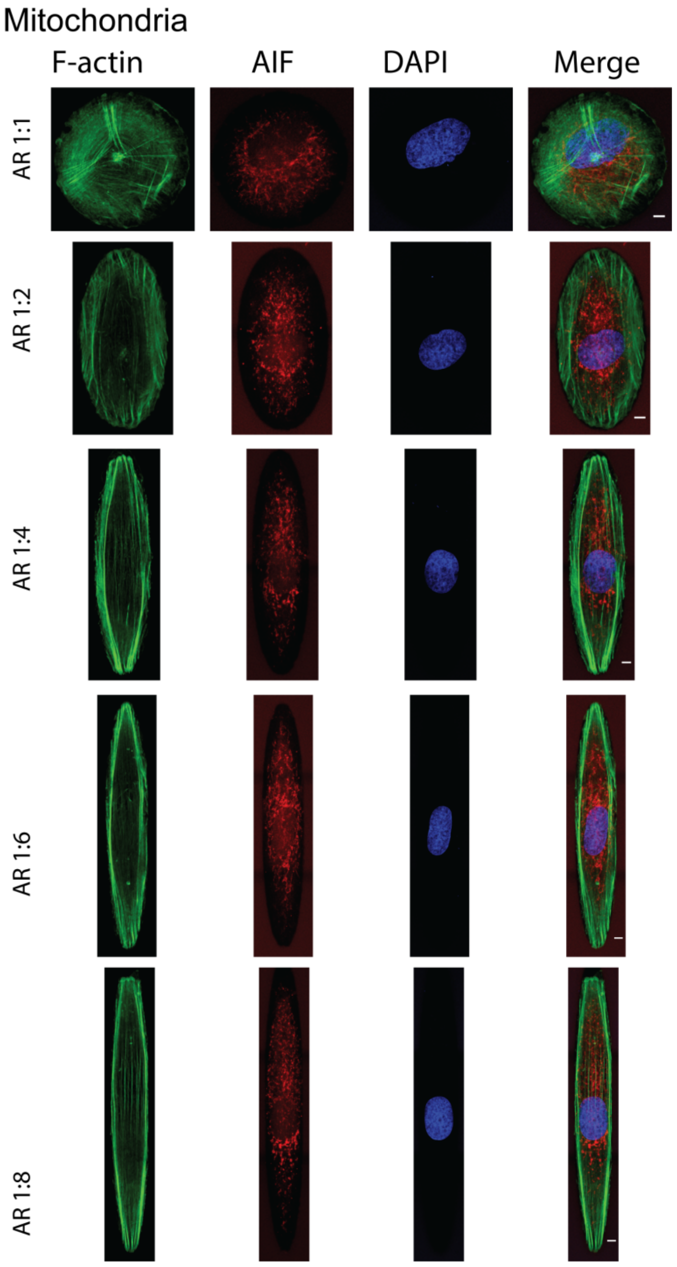

Supplementary Figure 2. Mitochondrial organization in VSMC with AR 1:1 to AR 1:8. Shown are staining of F-actin (green), mitochondrial marker Apoptosis Inducing Factor (AIF) (red) and DAPI in VSMC complying with AR 1:8. Scale bars shown in the merged channel are 10 µm.

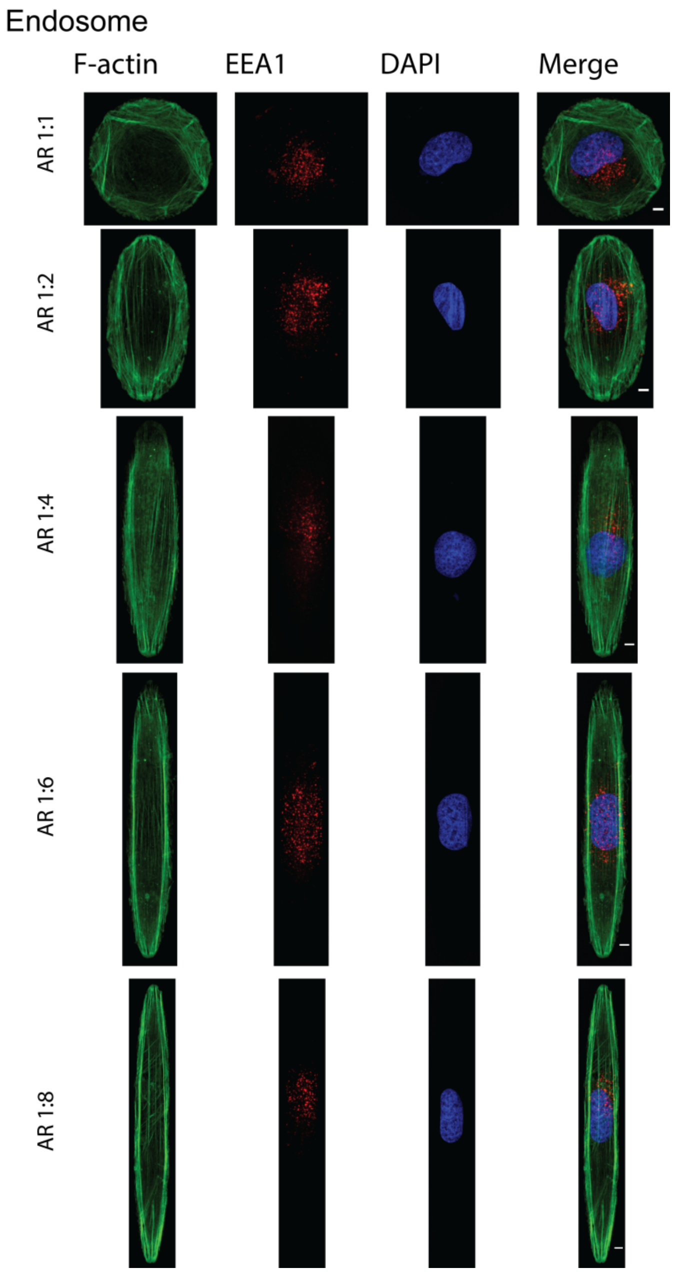

Supplementary Figure 3. Early endosome organization in VSMC with AR 1:1 to AR 1:8. Shown are staining of F-actin (green), endosomal marker Early Endosome Antigen 1 (EEA) (red) and DAPI in VSMC complying with AR 1:8. Scale bars shown in the merged channel are 10 µm.

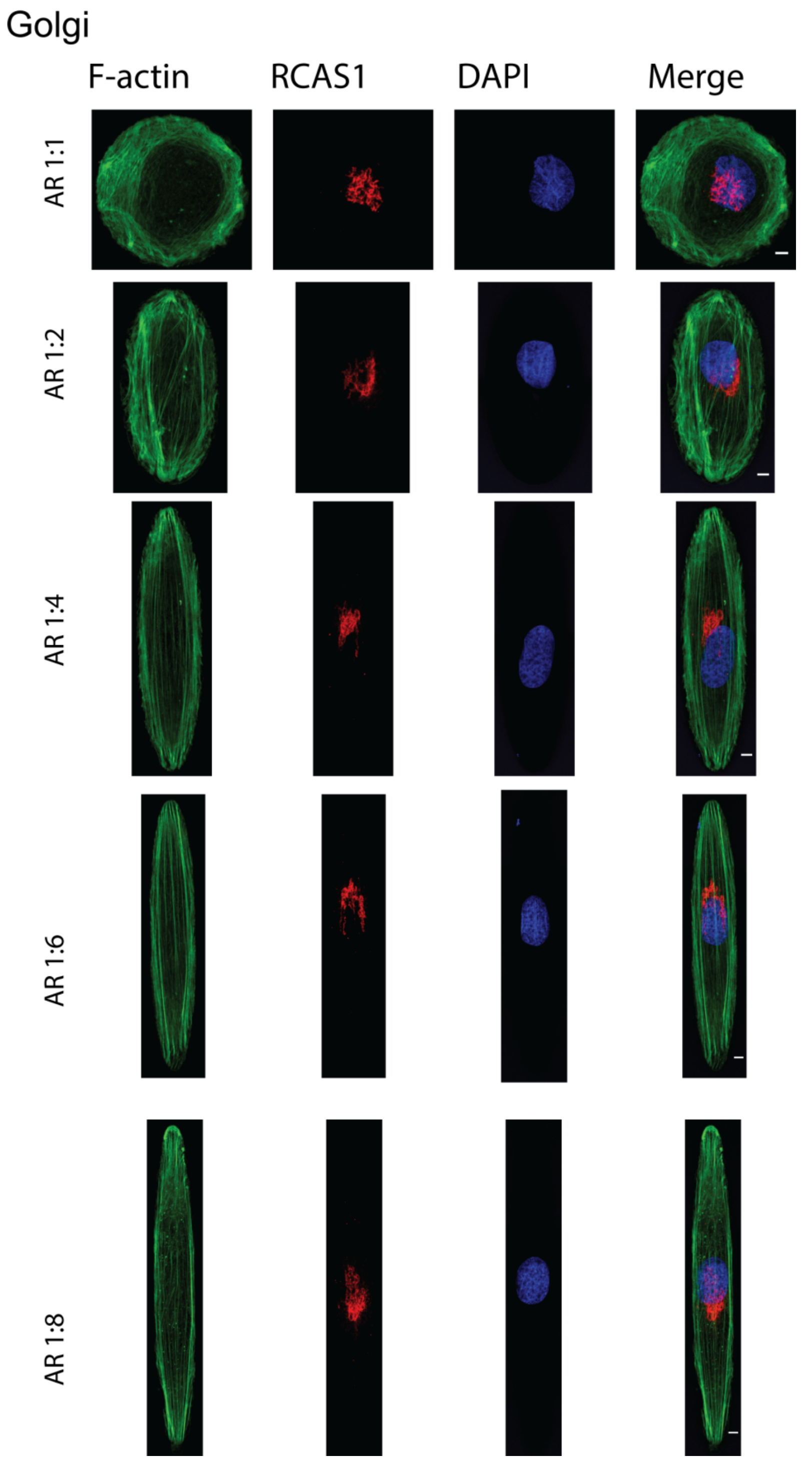

Supplementary Figure 4. Golgi organization in VSMC with AR 1:1 to AR 1:8. Shown are staining of F-actin (green), Golgi marker, receptor-binding cancer antigen expressed on SiSo cells (RCAS1) (red) and DAPI in VSMC complying with AR 1:8. Scale bars shown in the merged channel are 10 μm.

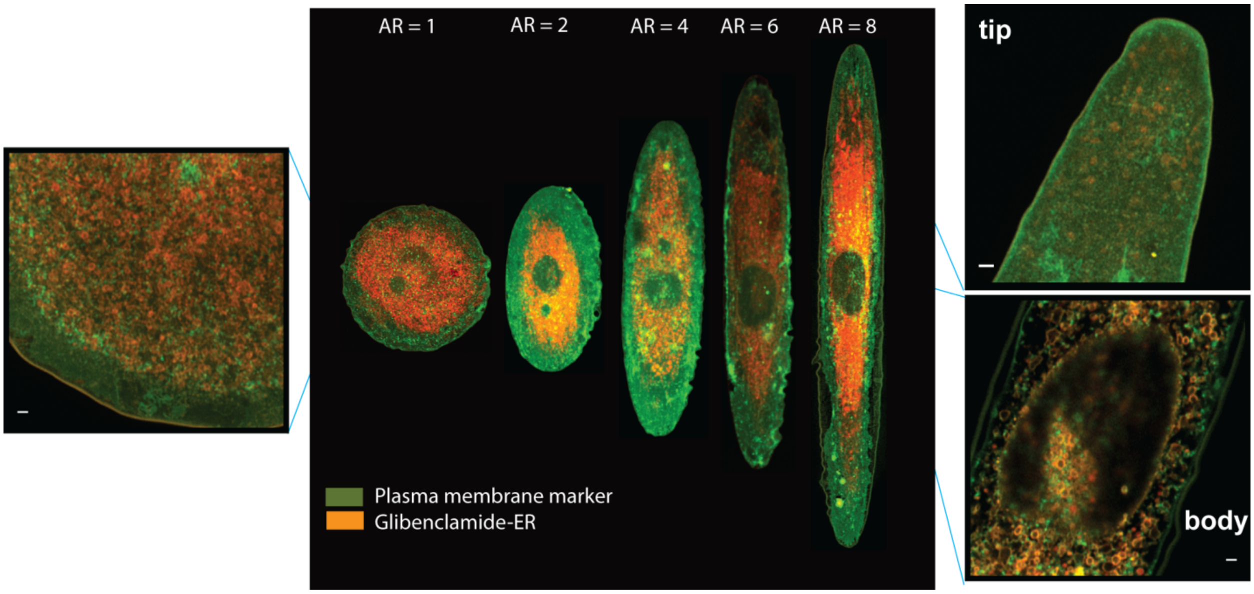

Supplementary Figure 5. Live cell Airy Scan imaging of SR membrane in VSMC with AR 1:1 to AR 1:8. Shown are confocal and Airy scan images (inset) of plasma membrane marker (CellMask Plasma Membrane marker, Invitrogen), green and SR marker (Bodipy-Glibenclamide ER) in red. Scale bars are 0.5 µm.

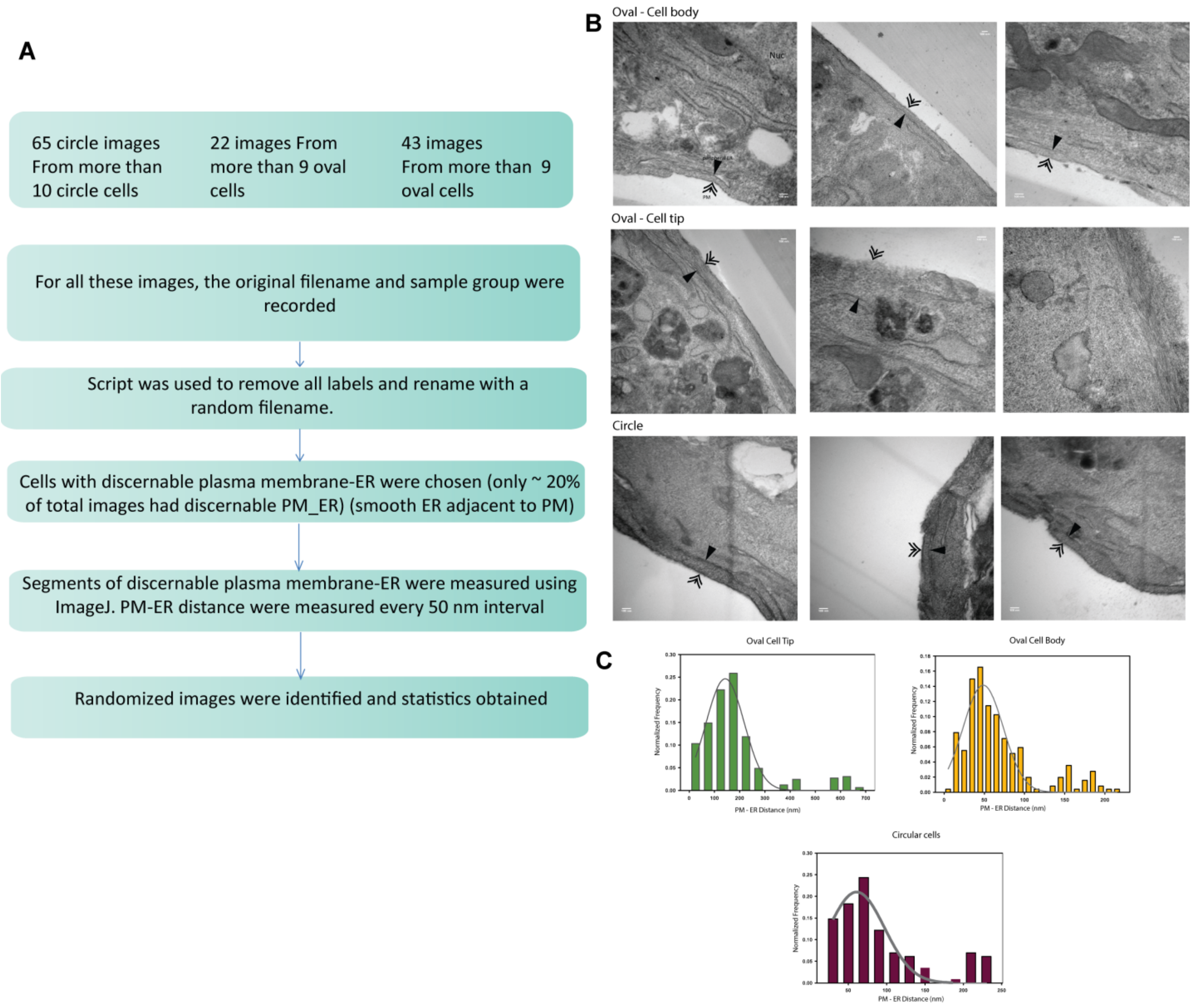

Supplementary Figure 6. TEM quantification of PM-SR distances from electron micrographs. Peripheral SR was identified by its close apposition to the plasma membrane and the absence of attached ribosomes. PM-peripheral SR was measured at 50-nm intervals. A) Post-imaging processing of EM micrographs to quantify PM-SR distances B) Representative images of cells from elliptical cell body, elliptical cell tip and circular cell tip. C) Histograms of distances obtained. Scale bars shown are 50 nm.

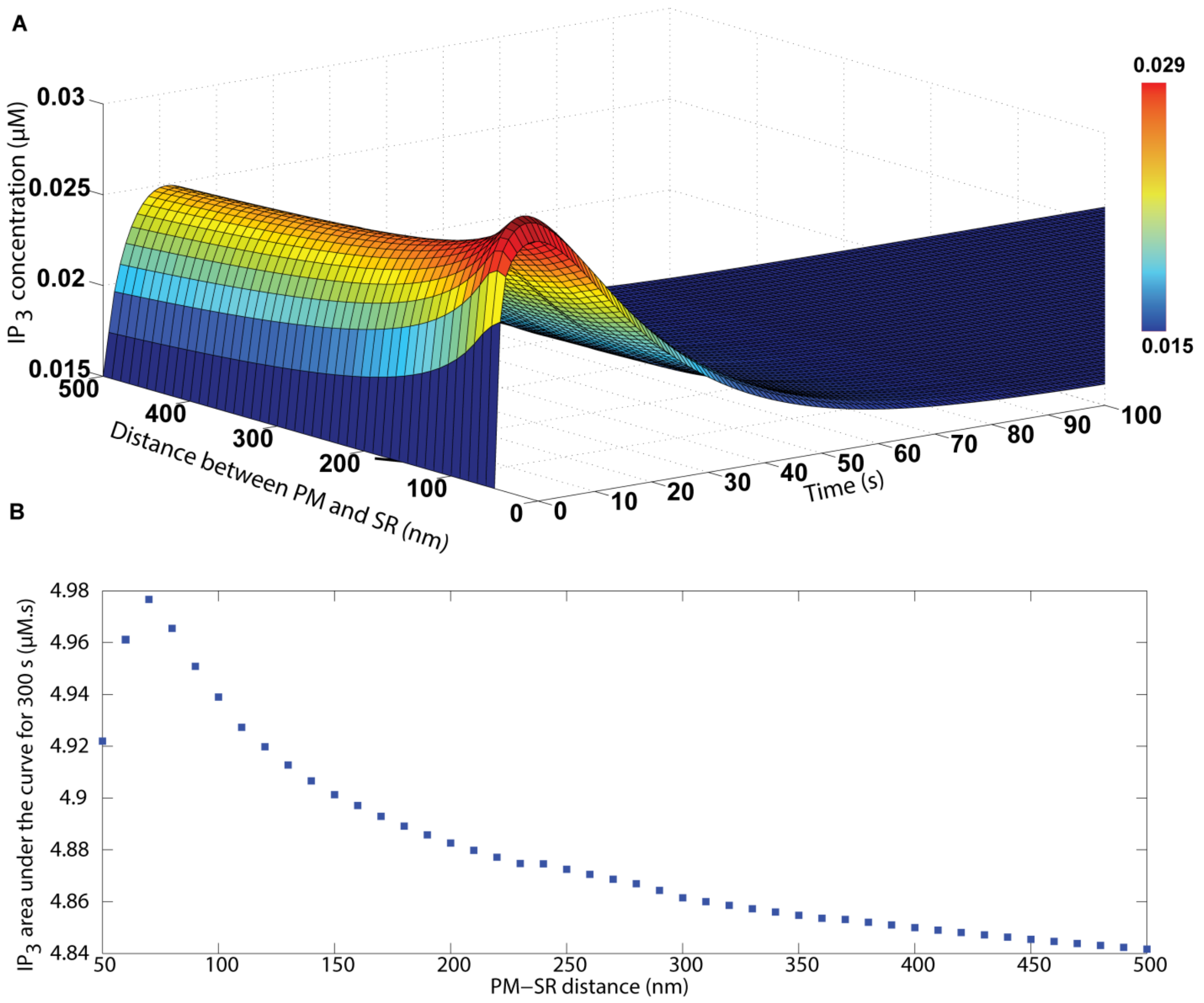

Supplementary Figure 7. The spatio-temporal profile of IP_3 at different PM-SR distances and resulting AUC from the COMSOL model. IP_3 concentration, at a location between the PM and SR, as a function of time for different PM-SR distances in rectangular geometries shown in Figure 3B. (B) AUC for IP_3 shows an increase between 50 and 80 nm with a peak at 70 nm and decreases for increasing PM-SR distances from there on, suggesting that the small PM-SR distances are best suited for IP_3 signaling.

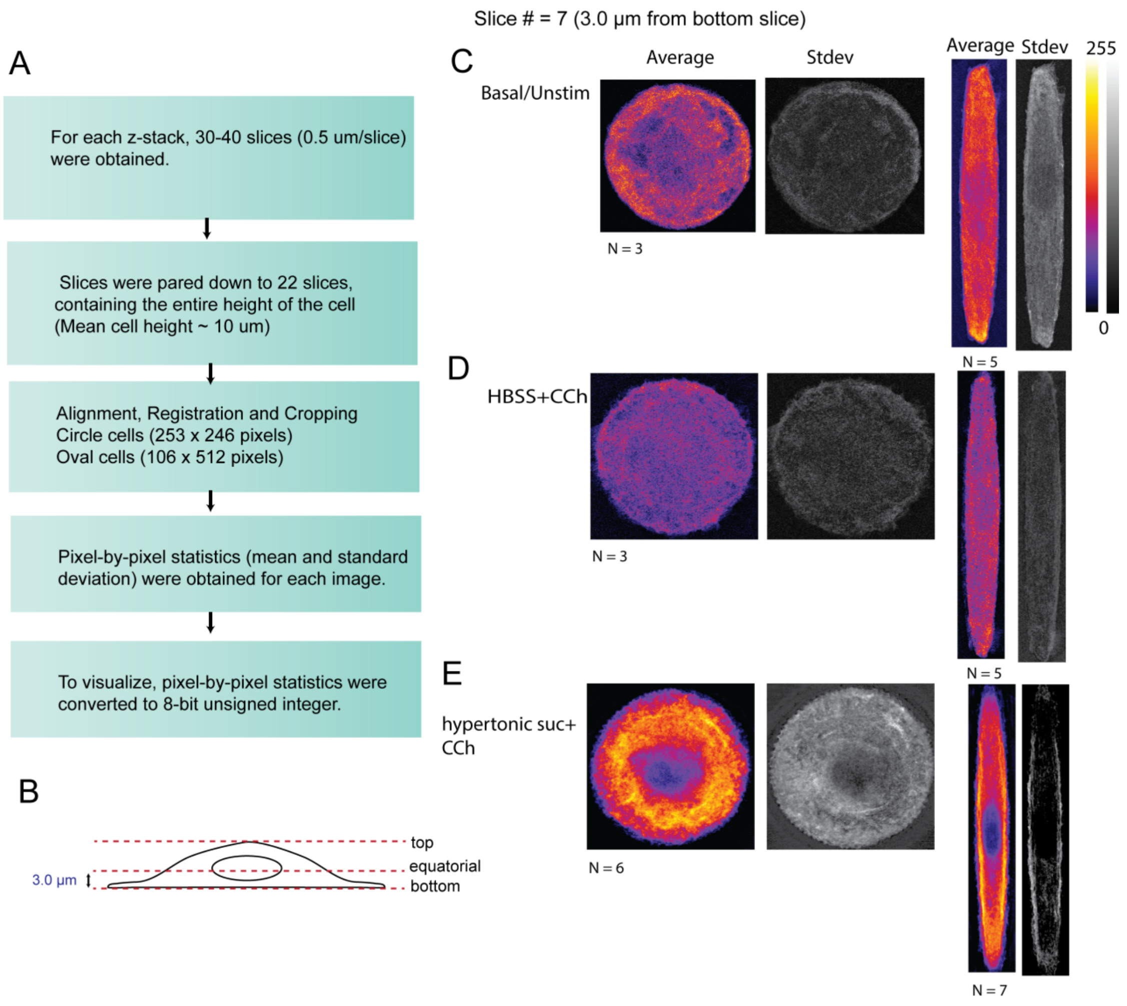

Supplementary Figure 8. Quantitative Immunofluorescence workflow to determine distribution of M3R under different conditions. A) Processing of Z-stacks obtained from individual cells B) Images were obtained at z = 3.0 µm from the bottom of the cell. Representative images obtained at C) basal/unstimulated D) stimulated with 10 µM Carbachol in HBSS and E) Stimulated with 10 µM Carbachol in the presence of hypertonic sucrose at 4 ºC to inhibit receptor endocytosis. Scalebars shown are 10 µm.

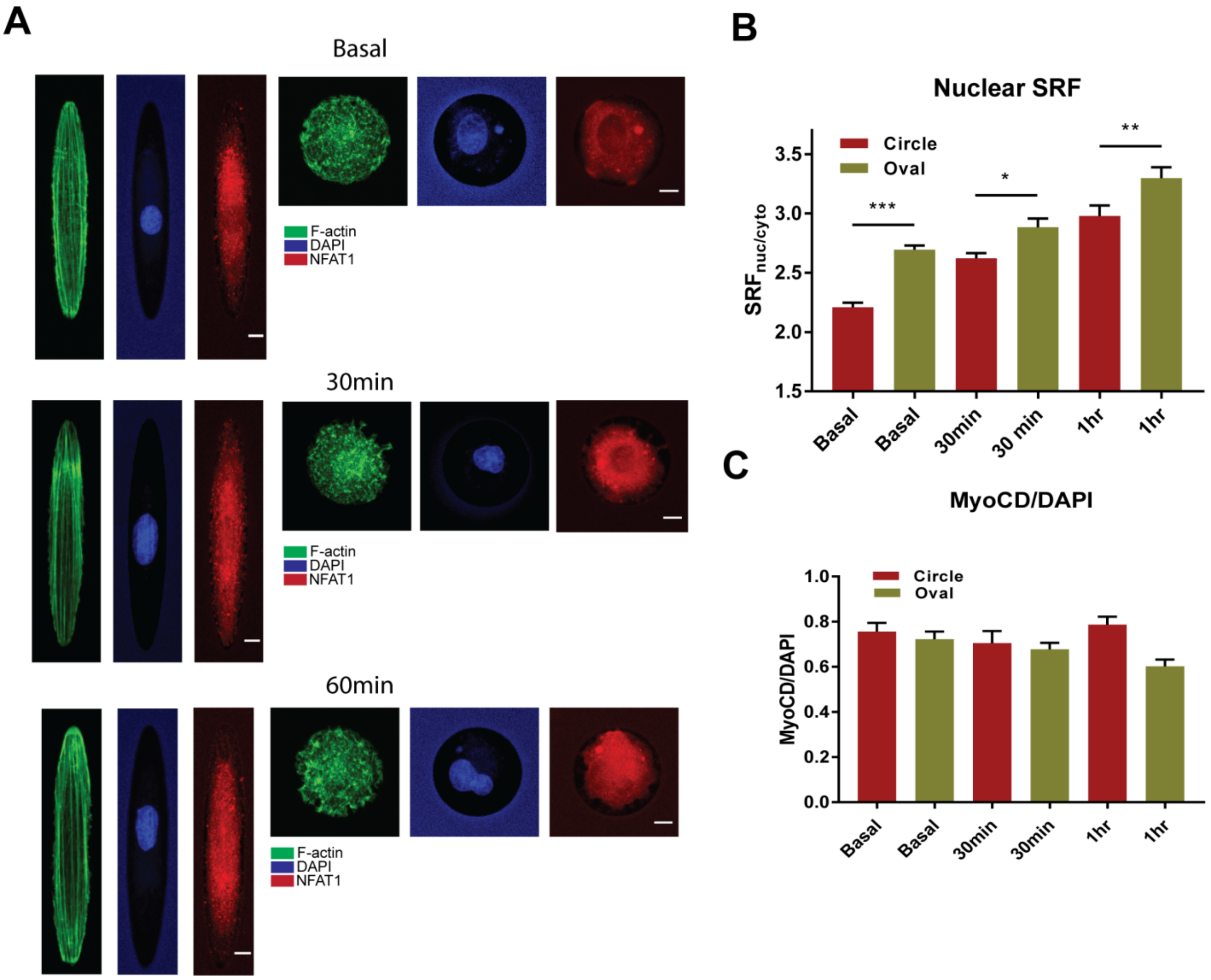

Supplementary Figure 9. NFAT and SRF are impacted by changes in shape while myocardin is not. A) Representative images of NFAT after stimulation with carbachol showing nuclear translocation of NFAT. Scale bars shown in NFAT (red) are 10 µm.

B) SRF localization into nucleus is impacted by cell shape (shown are mean $\pm$ SEM, $N_{circle,}{}^{basal} = 118$, $N_{oval,}{}^{basal} = 120$, $N_{circle,}{}^{30min} = 152$, $N_{oval,}{}^{30min} = 133$, $N_{circle,}{}^{1hr} = 99$, $N_{oval,}{}^{1hr} = 132$), C) MyoCD was constitutively in the nucleus. No change in myocd nuclear increase was seen with carbachol stimulation (shown are mean $\pm$ SEM, $N_{circle,}{}^{basal} = 17$, $N_{oval,}{}^{basal} = 5$, $N_{circle,}{}^{30min} = 9$, $N_{oval,}{}^{15min} = 17$, $N_{circle,}{}^{1hr} = 26$, $N_{oval,}{}^{1hr} = 13$). (*, **, *** denote $p < 0.05$, $p < 0.001$ and $p < 0.0001$ respectively, denoting statistical significance between the two cell shape, circle in red and oval in green, using two-tailed Student's t-test).

A

Cell Aspect Ratio	Compartment	Area (μm^2)	Perimeter (μm)
1:1	Cytoplasm	512	158
	ER	1301	446
	Nucleus	153	44
1:2	Cytoplasm	512	172
	ER	1297	310
	Nucleus	153	44
1:4	Cytoplasm	512	215
	ER	1293	390
	Nucleus	155	45
1:6	Cytoplasm	512	255
	ER	1300	460
	Nucleus	155	45
1:8	Cytoplasm	512	290
	ER	1304	511
	Nucleus	152	48
1:10	Cytoplasm	512	320
	ER	1307	582
	Nucleus	154	48

B

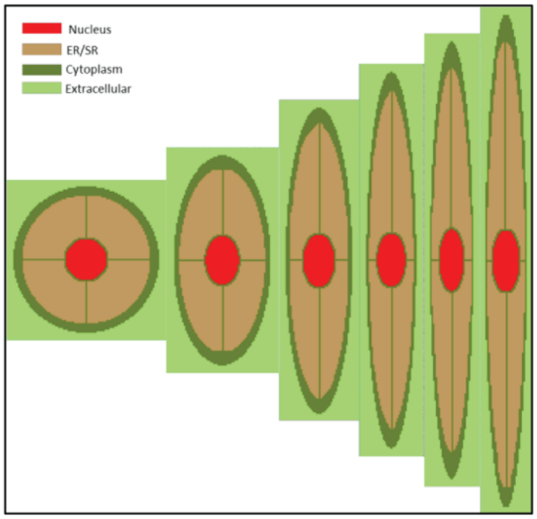

C

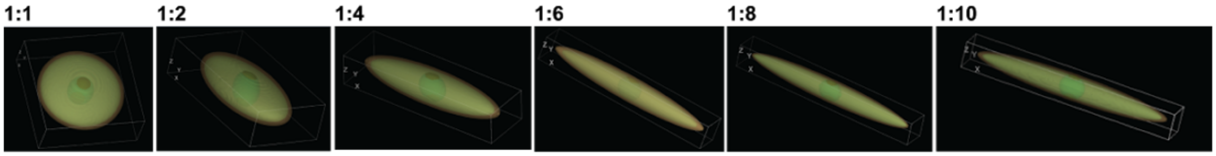

Supplementary Figure 10. A) Geometric region details used for the spatial model using *Virtual Cell* and B) resulting 2D geometries with increasing aspect ratio. C) 3D geometries of cells with constant volume and increasing surface area to volume ratio representing plasma membrane, SR membrane and nucleus in increasing elliptical aspect ratios.

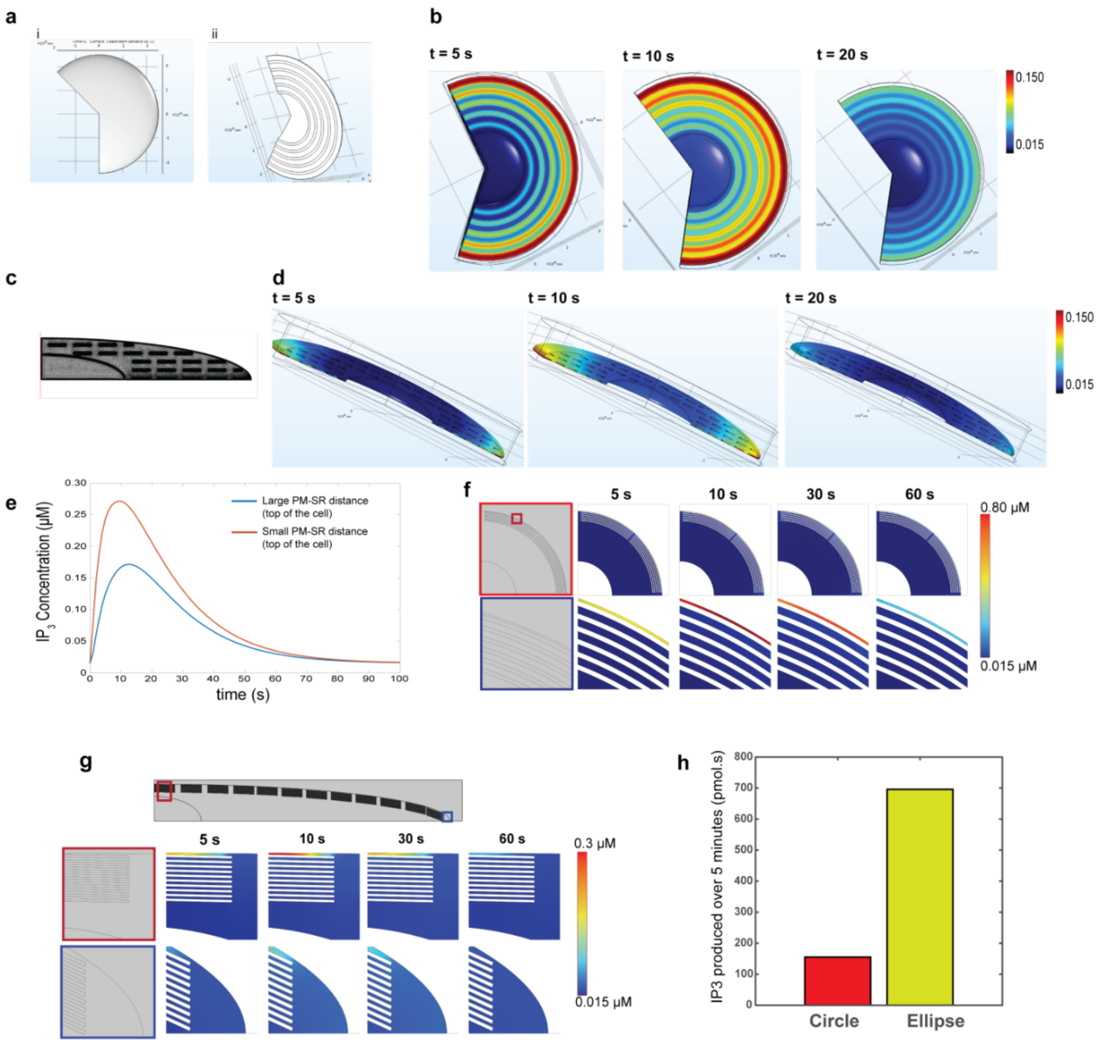

Supplementary Figure 11. 3D spatial models provide mechanistic understanding of cell elongation-induced Ca^{2+} potentiation. 3D simulations show the effect of cell elongation on inter-organelle distances on IP_3/Ca^{2+} potentiation using COMSOL a) 3D geometry to represent a cell with a circular 2D cross section and the ER as tori placed at different distances from the PM with the finite element mesh. b) IP_3 spatial dynamics at 5, 10, and 20s in the geometry shown in panel a. c) 3D geometry to represent a cell with an elliptical cross section with rings of ER. d) IP_3 spatial dynamics at 5, 10, and 20 s in the ellipsoidal geometry. e) IP_3 dynamics at two different points near and far from the PM showing the PM-SR distance affects IP_3 transience. f –h) IP_3 concentrations in 2D circular geometries (f) and 2D elliptical geometries (g). h) IP_3 concentrations over space (the entire domain) and time were integrated, since integration of signal over time provides a buffer for time scale and damps out the effect of temporal fluctuations and spatial integration allows for conversion from concentration (moles per unit volume) to amount (moles). Shown are graphs comparing integrated IP_3 in circular and elliptical geometries.

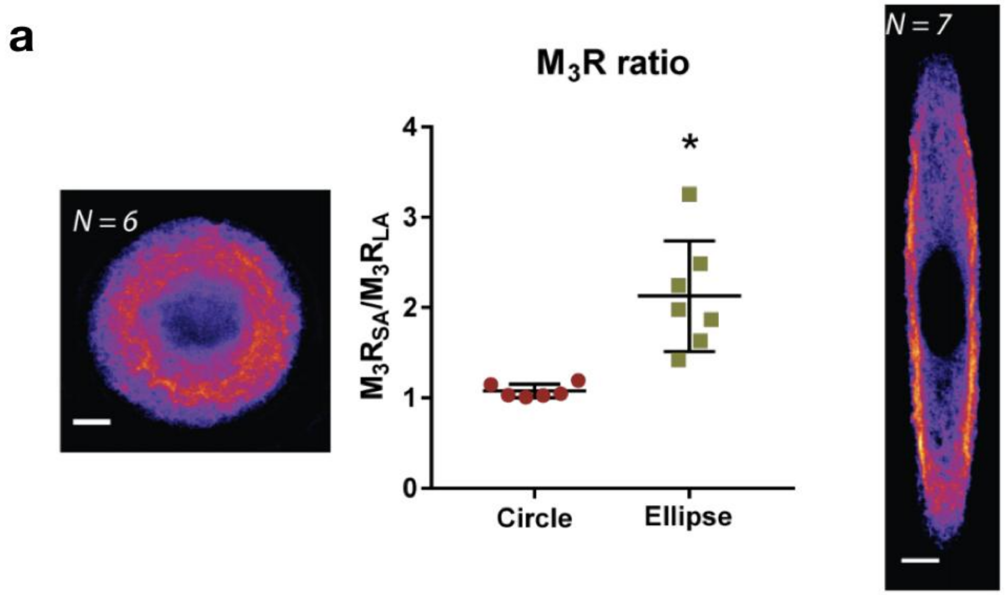

Supplementary Figure 12. Shape impacts M_3R/Ca^{2+} signaling in VSMC. (a) Average intensity of M_3R along the length of the cell in circular (*N=6)* and oval *(N=7)* VSMC. Differences in anisotropic distribution of M_3R were quantified by obtaining the ratio of M_3R staining intensities on the short and long axes (M_3R_{SA}/M_3R_{LA}, mean±SEM ellipse=2.1 ± 0.6 A.U. *N* = *7*, circle= 1.1 ± 0.1 A.U., *N*=*6*, *P=0.0016, two-tailed t-test). Scale bars shown are 10 μm.

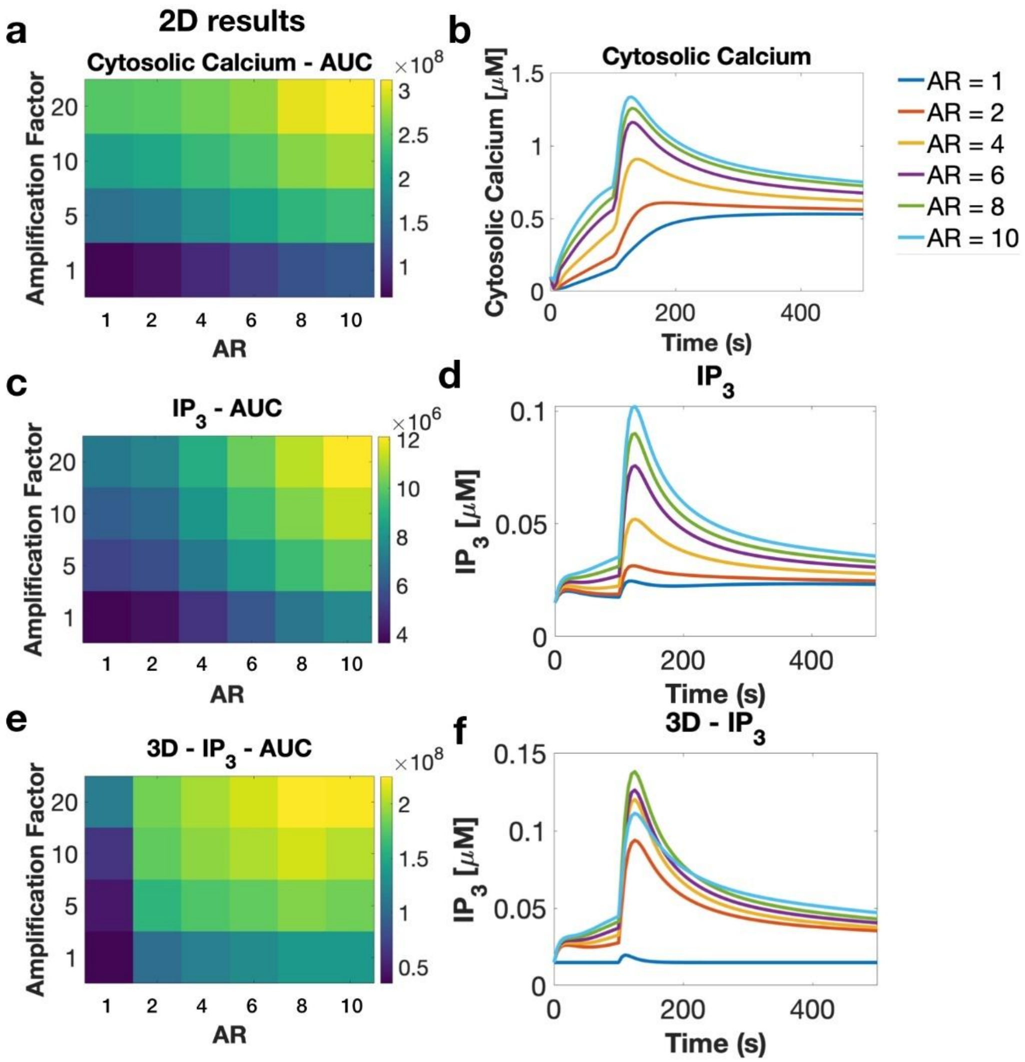

Supplementary Figure 13. Simulation results for 2D and 3D geometries. a-d) 2D simulation results for cytosolic calcium and IP_3 dynamics. AUC and temporal dynamics for different ARs and SR flux amplification factors. e-f) 3D simulation results for IP_3 dynamics for geometries of different ARs and SR flux amplification factors. Inset: legend for all temporal simulation results.

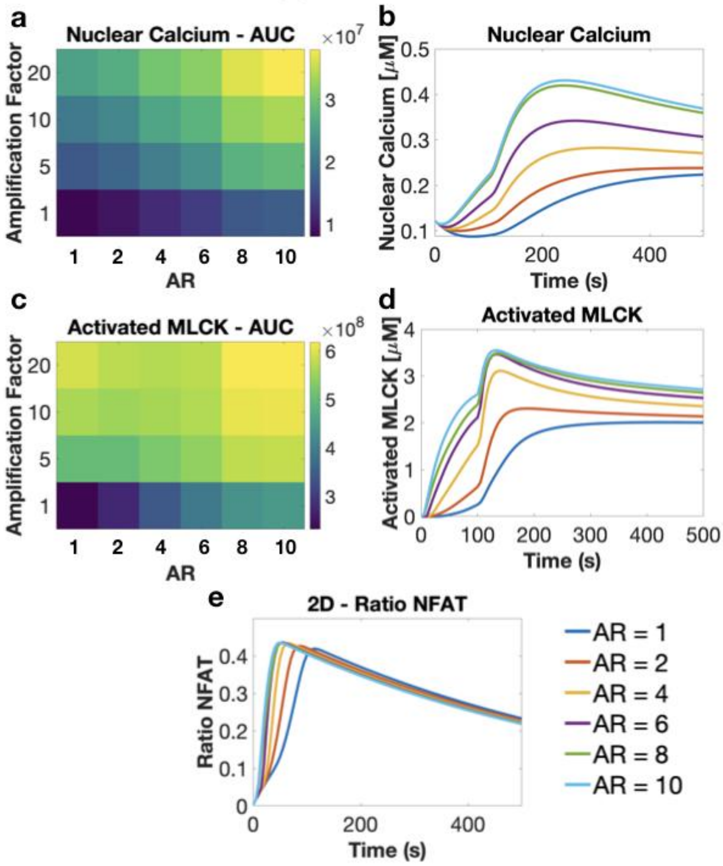

Supplementary Figure 14. 2D simulation results for downstream signaling. a-e) 2D simulation results for nuclear calcium, activated MLCK, and NFAT dynamics. AUC and temporal dynamics for different ARs and SR flux amplification factors. Inset: legend for all temporal simulation results.

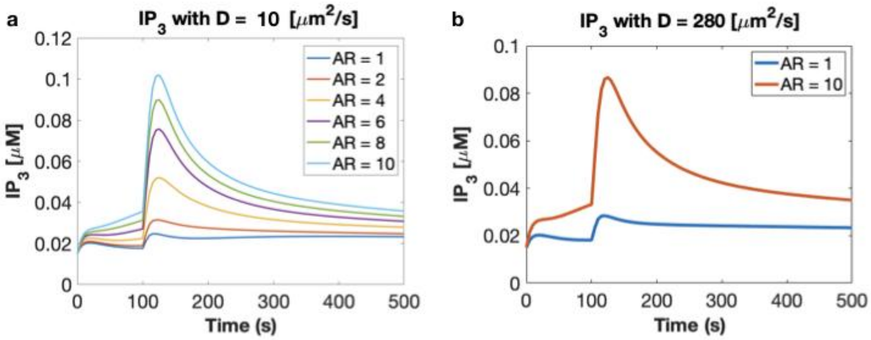

Supplementary Figure 15. Effect of IP_3 diffusion coefficient. a-b) IP_3 dynamics for different IP_3 diffusion coefficients 10 versus 280. We see the same trend regardless of diffusion coefficient.

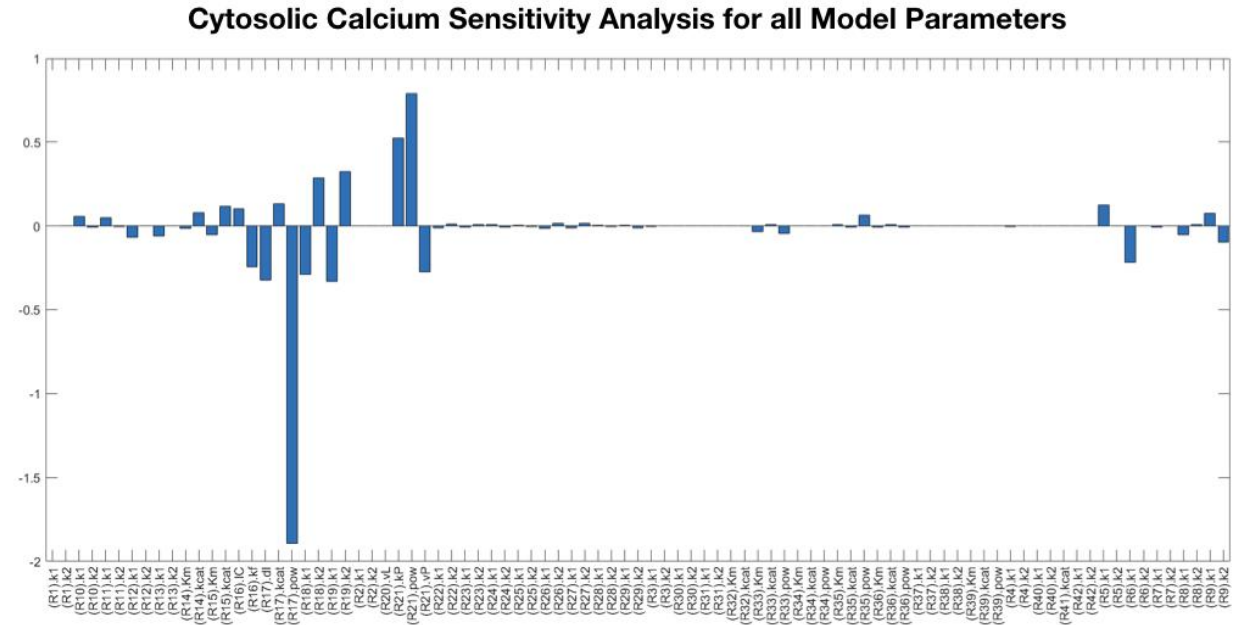

Supplementary Figure 16. Sensitivity Analysis shows how different model parameters affect cytoplasmic calcium transients. The model is particularly sensitive to R17, which means that calcium release from the SR due to IP3R is particularly important to the model.

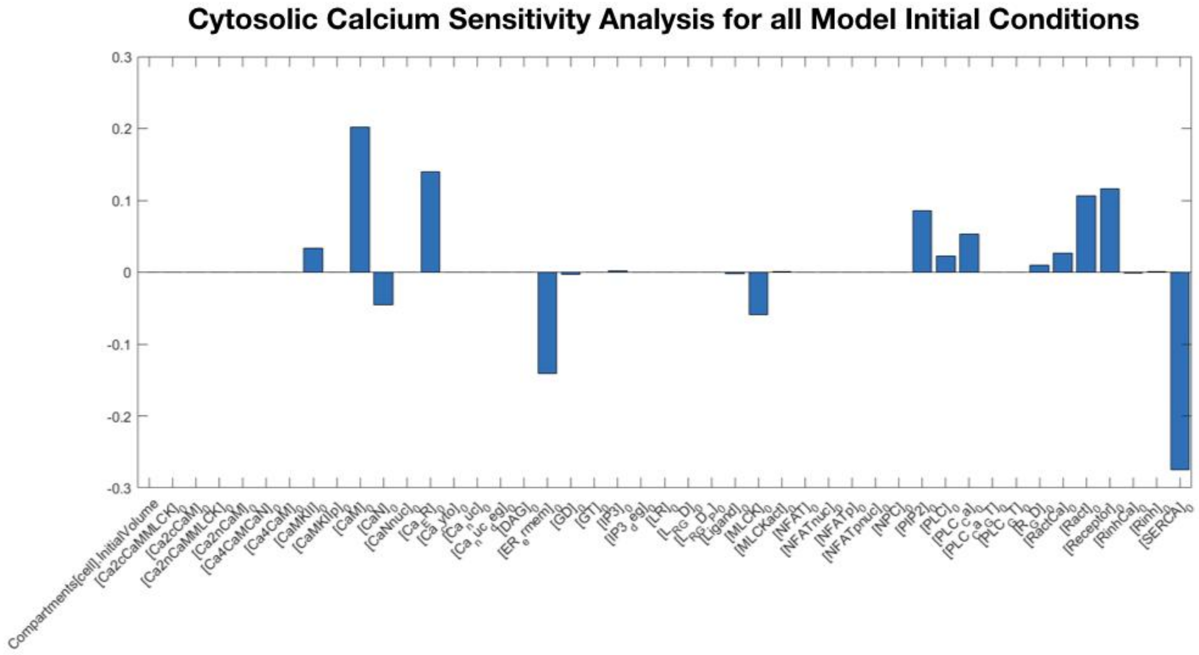

Supplementary Figure 17. Sensitivity Analysis for cytoplasmic calcium transients with respect to model initial conditions. We see that several species have a larger impact than others. In particular, cytoplasmic calcium is sensitive to SERCA and ER_{ermem} density, and CaM and Ca_{ER} initial concentrations.

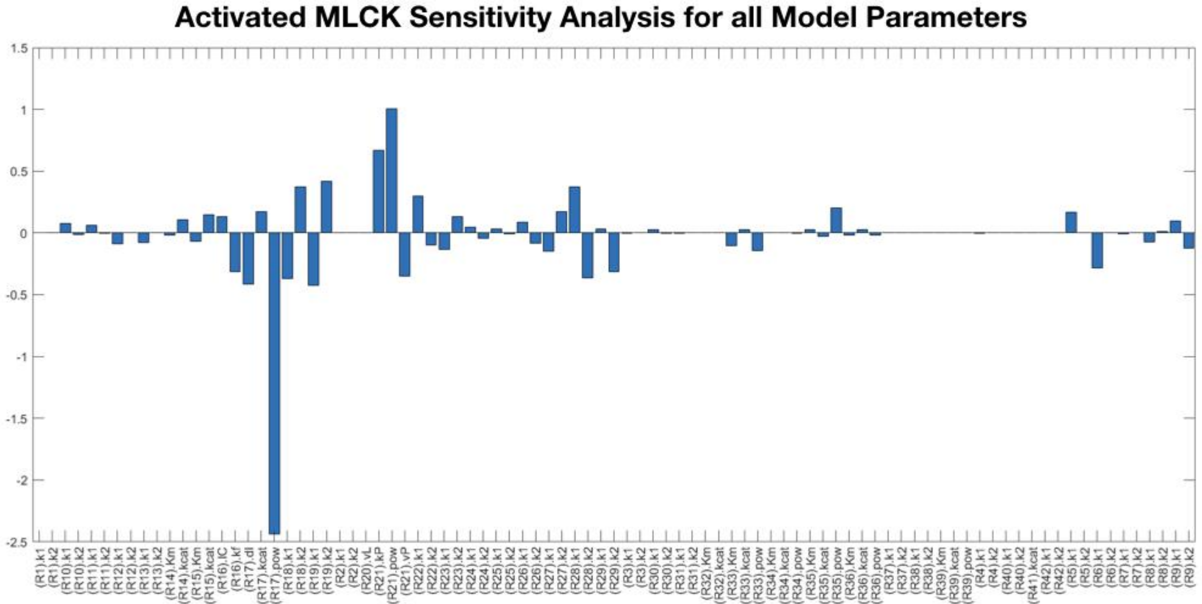

Supplementary Figure 18. Sensitivity Analysis shows how all model parameters affect activated MLCK transients. We see that similarly to cytoplasmic calcium, activated MLCK depends on calcium dynamics associated with the SR, R17 and R21 in particular.

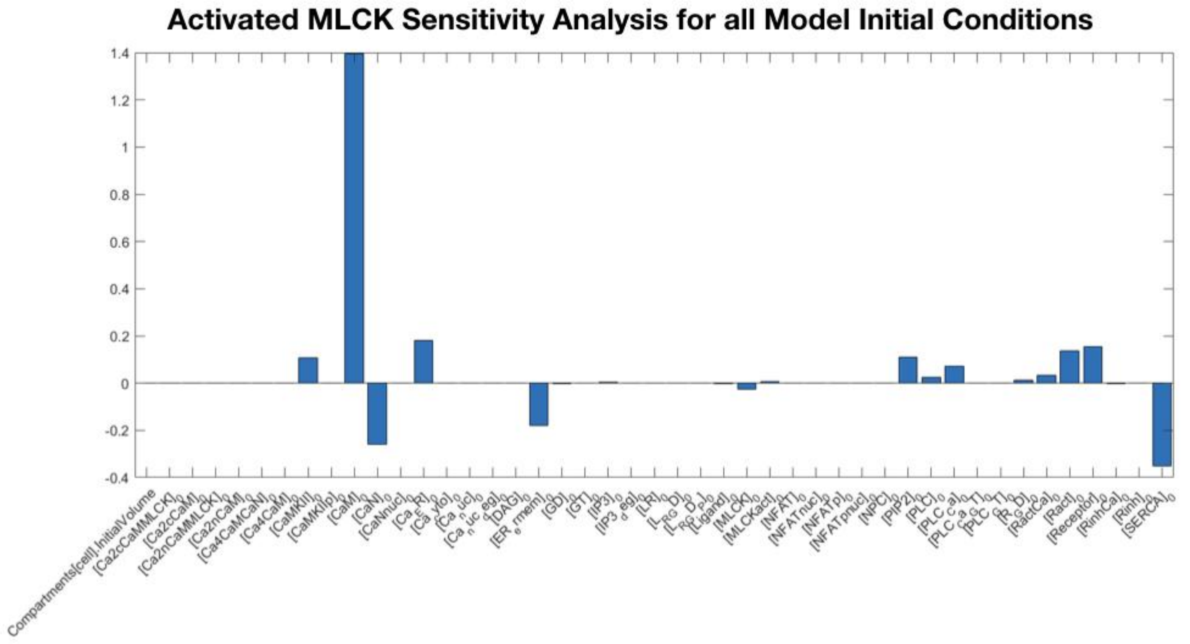

Supplementary Figure 19. Sensitivity Analysis for activated MLCK transients with respect to model initial conditions. Activated MLCK is particularly sensitive to CaM initial concentration which makes sense because MLCK activation directly depends on CaM.

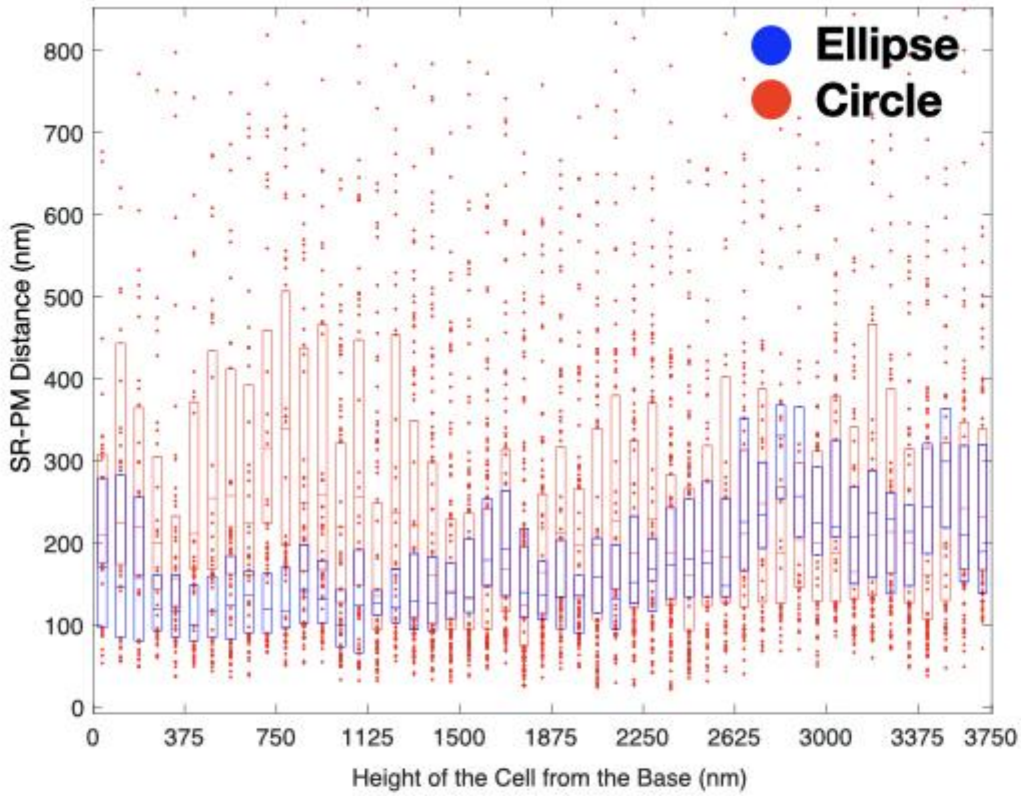

Supplementary Figure 20. 3D serial block face scanning electron microscopy was used to quantify the PM-SR distance in circular versus elliptical cells. The PM-SR distance per dyad was computed at each axial position and plotted as a function of representative circular and elliptical cells. 5309 and 1692 PM-SR distances were taken for the circular and elliptical cells, respectively.

RAW CALCIUM DATA AND ANALYSES

Experimental calcium traces, their raw data, and the MATLAB script used to calculate their AUCs are listed below. Results from the data are found in Figures 5 and 6.
Each plot represents a cell conforming to a micropattern.

RAW PLOTS FOR CYTOSOLIC CIRCLE

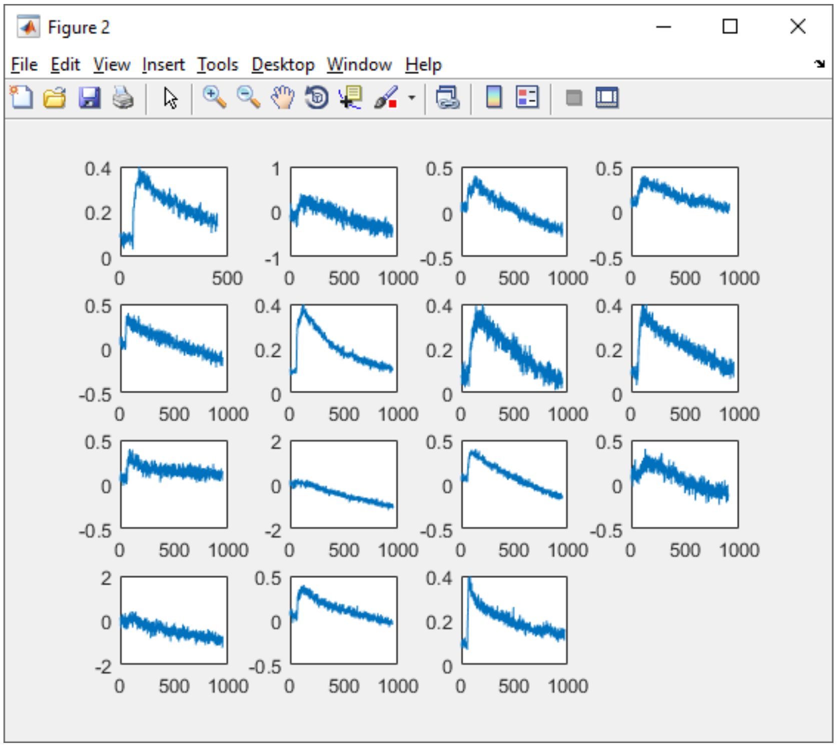

RAW DATA FOR NUCLEAR CIRCLE

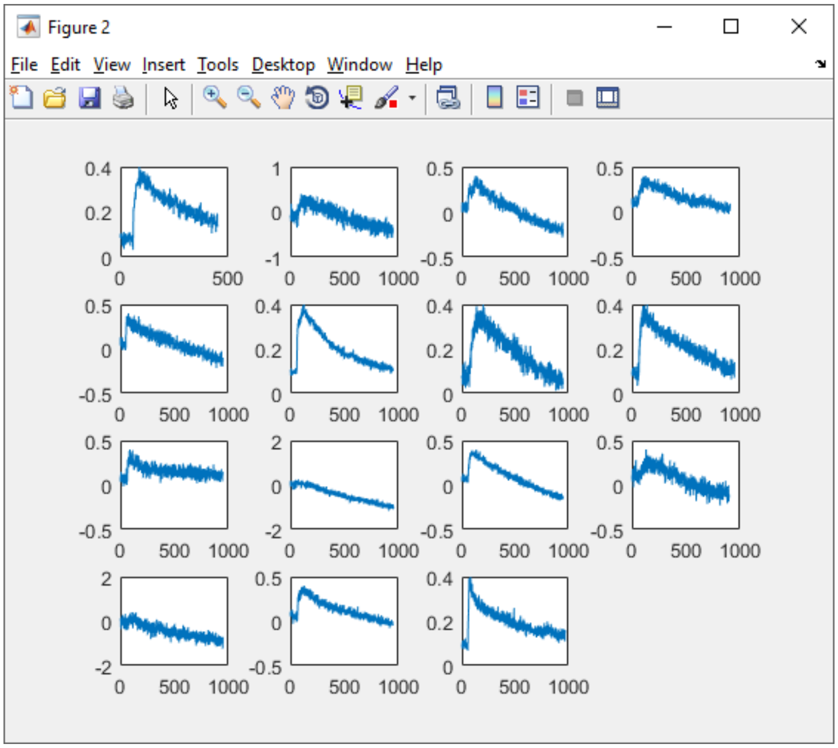

RAW DATA FOR CYTOSOLIC OVAL

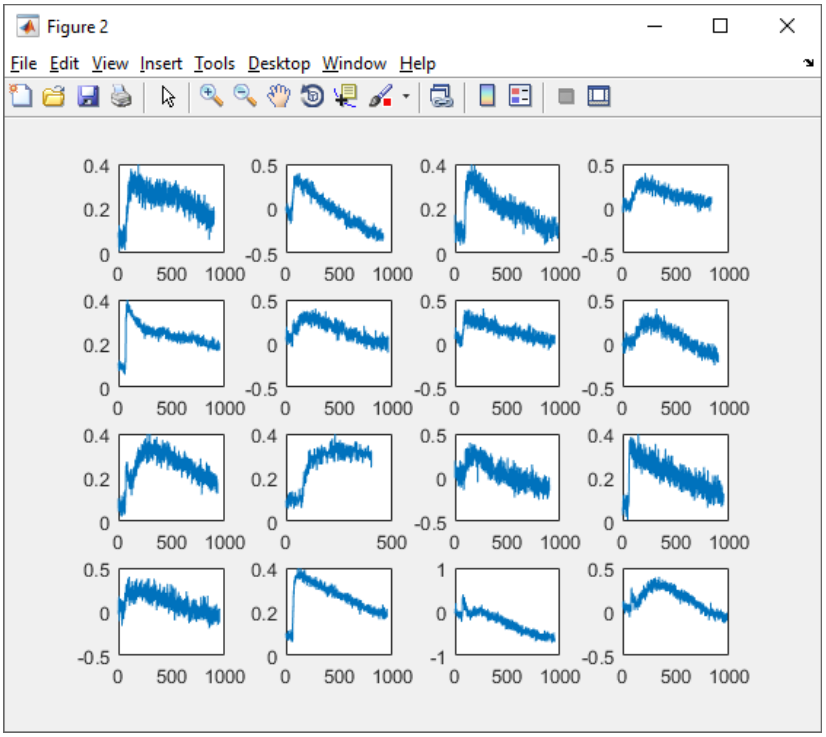

RAW DATA FOR NUCLEAR OVAL

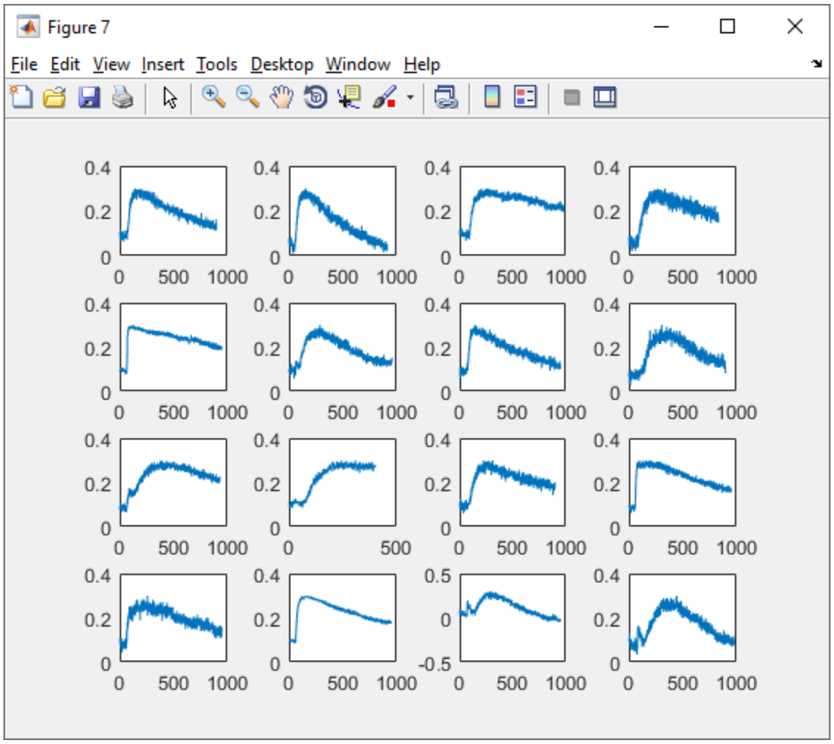

RAW DATA OF EXPERIMENTAL AUC OBAINED USING THE CUSTOM-MADE AUC FUNCTION

CYTOSOLIC CALCIUM

Circle	Oval
99.10929	220.4497
115.7618	110.1947
164.0739	192.8608
86.57138	227.0699
185.7712	157.679
186.5009	203.7415
167.3561	118.8767
165.1082	223.3568
31.51364	112.7875
100.7844	191.146
102.2025	125.671
48.92968	240.0209
158.9907	61.16456
181.0728	157.637

NUCLEAR CALCIUM

Circle	Oval
247.1468	224.5708
203.6572	212.9696
231.6694	258.1552
160.1104	271.9229
208.7487	198.0883
215.0813	207.7107
208.4184	216.9704
232.57	260.6754
163.7488	228.6693
172.7847	261.4111
153.4669	223.6992
186.8566	270.0343
193.1922	156.9277
231.2051	192.417

SCRIPTS FOR ANALYZING IMAGING DATA FOR ELLIPSE AND CIRCULAR CELLS
Brief Description of the scripts

Raw movie data were first imported using imageJ. An ROI with a specified size was used to specify nuclear and cytosolic regions. Raw intensity data was then normalized based on BAPTA and 100 uM A23187. AUC was calculated using the trapz function, as described by the following:

```
function [auc_ans] = auc2(data, start_time,interval)
%data = circ_cyt_conc_tc;
%start_time = 50;
%interval = 400;
col = size(data,2);
end_time = interval + start_time;
auc_ans = nan(size(data,2), 1);
for i = 1:col
   data2 = data(:,i);
   %auc_ans(i) = trapz(data2(start_time:end_time))-trapz([start_time end_time],
[data2(start_time) data2(end_time)]);
```

```
    auc_ans(i) = trapz(data2(start_time:end_time))-trapz([start_time end_time],[data2(start_time) 
data2(start_time)]);
end
auc_ans = auc_ans(:);
```

MATLAB SCRIPT FOR ANALYZING IMAGING DATA FOR ELLIPSE AND CIRCULAR CELLS

```
%% To make normalized matrices
all_norm_int_nuc = [];
all_norm_int_cyt = [];

%% Opening raw movie files showing calcium data

IJ = ij.IJ();
macro_path_nuc = 'Macr_nuc.ijm';
IJ.runMacroFile(java.lang.String(macro_path_nuc)); % Extracting data for the nuclear region
nuc = MIJ.getResultsTable;
int_nuc = nuc(:,2);
norm_int_nuc = int_nuc/mean(int_nuc(1:20));
macro_path_cyt = 'Macr_cyt.ijm';
IJ.runMacroFile(java.lang.String(macro_path_cyt)); % Extracting data for the cytosolic region
cyt = MIJ.getResultsTable;
int_cyt = cyt(:,2);
norm_int_cyt = int_cyt/mean(int_cyt(1:20));
plot(1:size(norm_int_nuc,1), norm_int_nuc, 'ro', 1:size(norm_int_cyt,1),norm_int_cyt, 'bo');
norm_int_cyt(end+1:2000) = NaN;
norm_int_nuc(end+1:2000) = NaN;
all_norm_int_nuc = [all_norm_int_nuc, norm_int_nuc];
all_norm_int_cyt = [all_norm_int_cyt, norm_int_cyt];
```

IMAGEJ SCRIPT FOR ANALYZING CALCIUM DATA

%%ImageJ macros
Macr_nuc.ijm

```
k = getTitle();
run("Z Project...", "projection=[Max Intensity]");
selectWindow('MAX_'+k);
roiManager("reset");
run("Specify...", "width=5 height=5 x=115.17 y=115.17 oval constrain centered scaled");
run("In [+]");
run("In [+]");
waitForUser("select nuclear ROI");
roiManager("Add");
roiManager("Select",0);
selectWindow(k);
roiManager("Multi Measure");
```

Macr_cyt.ijm

```
k = getTitle();
run("Z Project...", "projection=[Max Intensity]");
selectWindow('MAX_'+k);
roiManager("reset");
run("Specify...", "width=5 height=5 x=115.17 y=115.17 oval constrain centered scaled");
run("Clear Results");
waitForUser("select cytosol ROI");
roiManager("Add");
roiManager("Select",0);
selectWindow(k);
roiManager("Multi Measure");
while(nImages>0) {
                                        selectImage(nImages);
                        close();
```

```
                                        }

for tt = 1:size(data,2)%go col by col, 1 col == 1 cell
    sample = data(:,tt);    time = 1:length(sample);
    figure
    k = round(linspace(1,1000,20));
    plot(sample)
    pause
    min_guess = input('Min time?');
    delta_t = 200;
    for i = 1:length(k);
        grad_sample = diff(sample, k(i))/delta_t;
        [max_val, loc] = max(sample);
        [pred_val, pred_loc] = max(abs(grad_sample(min_guess:loc)));
        subplot(4,5,i)
        plot(time, sample, (pred_loc+min_guess), sample(pred_loc+min_guess), 'ro')
        title(num2str(pred_loc+min_guess))
    end
    pause %a figure will show with the dif guess peaks and the time stimulation as title
end

%% make all_norm matrices
all_norm_int_nuc = [];
all_norm_int_cyt = [];
all_int_nuc = [];
all_int_cyt = [];
IJ = ij.IJ();
macro_path_nuc = 'Macr_nuc.ijm';
IJ.runMacroFile(java.lang.String(macro_path_nuc)); % executing the macro for the nuclear region
nuc = MIJ.getResultsTable; %exporting the results from the nuclear intensities
int_nuc = nuc(:,2);
norm_int_nuc = int_nuc/mean(int_nuc(1:20)); % normalizing the nuclear intensities
macro_path_cyt = 'Macr_cyt.ijm';
IJ.runMacroFile(java.lang.String(macro_path_cyt)); % executing macro for the cytosolic region
cyt = MIJ.getResultsTable;
int_cyt = cyt(:,2);
norm_int_cyt = int_cyt/mean(int_cyt(1:20));
plot(1:size(norm_int_nuc,1), norm_int_nuc, 'ro', 1:size(norm_int_cyt,1),norm_int_cyt, 'bo');
%% Add appropriate curves to the all_matrix
norm_int_cyt(end+1:2000) = NaN; % padding NaNs to the matrices
norm_int_nuc(end+1:2000) = NaN;
int_cyt(end+1:2000) = NaN;
int_nuc(end+1:2000) = NaN;
all_norm_int_nuc = [all_norm_int_nuc, norm_int_nuc];
all_norm_int_cyt = [all_norm_int_cyt, norm_int_cyt];
all_int_nuc = [all_int_nuc, int_nuc];
all_int_cyt = [all_int_cyt, int_cyt];
```

%Time Normalization

```
function [data_corr,ave_plot] = time_norm(data, time_stim)
padding = 50;
for i = 1:size(data,2)
    data_local = data(:,i);
    time_start = time_stim(i)- padding;
    corr_time = data_local(time_start:length(data_local)); %extract corrected_time
    corr_time(length(corr_time)+1:length(data_local)) = NaN;
    data_corr(:,i) = corr_time;
end
figure
plot(1:length(data_local), data_corr)
title('All plots');
ave_plot = (mean(data_corr'))';
figure
plot(1:2000, ave_plot)
title('average_plot')


function new_vector = minmaxnorm(vector, new_max, new_min)
new_vector = ((vector-min(vector))/(max(vector)-min(vector)))*(new_max-new_min)+new_min;
```

```
function new_vector = minmaxnorm(mat, new_max, new_min)
new_vector = nan(size(mat,1), size(mat,2));
for i = 1:size(mat,2);
    vector = mat(:,i);
    baseline = mean(vector(1:40));
    new_vector(:,i) = ((vector-baseline)/(max(vector)-baseline)*(new_max-new_min))+new_min;
end

function [data_corr,ave_plot] = time_norm(data, time_stim)
padding = 50;
figure
for i = 1:size(data,2)
    data_local = data(:,i);
    time_start = time_stim(i)- padding;
    corr_time = data_local(time_start:length(data_local)); %extract corrected_time
    corr_time(length(corr_time)+1:length(data_local)) = NaN;
    time = 1:length(data_local);
    data_corr(:,i) = corr_time;
    subplot(4,4, i)
    plot(time, corr_time);
    %ylim([0, 0.5])
end

ave_plot = (nanmean(data_corr'))';
figure
plot(1:2000, ave_plot)
title('average_plot')

circ_cyt_int_norm = minmaxnorm(circ_cyt_int, 0.4, 0.1);
plot(1:2000,circ_cyt_int_norm)
oval_cyt_int_norm = minmaxnorm(oval_cyt_int, 0.4,0.1);
circ_nuc_int_norm = minmaxnorm(circ_nuc_int, 0.4,0.1);
oval_nuc_int_norm = minmaxnorm(oval_nuc_int, 0.3,0.1);

[circ_cyt_conc_tc, circ_cyt_ave] = time_norm(circ_cyt_int_norm, circ_start);
[circ_nuc_conc_tc, circ_nuc_ave] = time_norm(circ_nuc_int_norm, circ_start);
[oval_cyt_conc_tc, oval_cyt_ave] = time_norm(oval_cyt_int_norm, oval_start);
[oval_nuc_conc_tc, oval_nuc_ave] = time_norm(oval_nuc_int_norm, oval_start);

figure; plot(1:2000, [circ_cyt_ave, oval_cyt_ave]);
figure; plot(1:2000, [circ_nuc_ave, oval_nuc_ave]);
```

CONVERSION OF RATIOMETRIC TO CONCENTRATION

```
circ_cyt_rat_conc = minmaxnorm(circ_cyt_rat, 0.4, 0.1);
oval_cyt_rat_conc = minmaxnorm(oval_cyt_rat, 0.4, 0.1);
circ_nuc_rat_conc = minmaxnorm(circ_nuc_rat, 0.4, 0.1);
oval_nuc_rat_conc = minmaxnorm(oval_nuc_rat, 0.4, 0.1);

%time normalize the cells
[circ_cyt_tc, circ_cyt_ave] = time_norm(circ_cyt_rat_conc,circ_start);
[circ_nuc_tc, circ_nuc_ave] = time_norm(circ_nuc_rat_conc,circ_start);
[oval_cyt_tc, oval_cyt_ave] = time_norm(oval_cyt_rat_conc,oval_start);
[oval_nuc_tc, oval_nuc_ave] = time_norm(oval_nuc_rat_conc, oval_start);

endtime_set = [800, 700, 600, 500, 400, 300, 200];
for i = 1:length(endtime_set)
    endtime = endtime_set(i);
    % calculate for auc
    auc_circ_cyt = auc2(circ_cyt_tc, 50, endtime);
    auc_oval_cyt = auc2(oval_cyt_tc, 50, endtime);
    auc_circ_nuc = auc2(circ_nuc_tc, 50, endtime);
    auc_oval_nuc = auc2(oval_nuc_tc, 50, endtime);
    auc_circ_cyt = padnan(auc_circ_cyt, 20);
    auc_oval_cyt = padnan(auc_oval_cyt, 20);
    auc_circ_nuc = padnan(auc_circ_nuc, 20);
```

```
    auc_oval_nuc = padnan(auc_oval_nuc, 20);
    figure; subplot(1,2,1); boxplot([auc_circ_cyt, auc_oval_cyt])
    subplot(1,2,2); boxplot([auc_circ_nuc, auc_oval_nuc])
end

figure; boxplot([auc_circ_cyt, auc_oval_cyt]);
figure; boxplot([auc_circ_nuc, auc_oval_nuc]);

AAA = [auc_circ_cyt, auc_oval_cyt, auc_circ_nuc, auc_oval_nuc];
[H,P] = ttest2(AAA(:,3), AAA(:,4));



time = unnamed(:,1);
circle = unnamed(:,2);
oval = unnamed(:,3);
start_time = 1;
end_time = 800;
circle_AUC = trapz(circle(start_time:end_time))-trapz([start_time end_time], [circle(start_time) circle(end_time)]);
oval_AUC = trapz(oval(start_time:end_time))-trapz([start_time end_time], [oval(start_time) oval(end_time)]);
function AUC = auc(vector, start_time, end_time)
AUC = trapz(vector(start_time:end_time))-trapz([start_time end_time], [vector(start_time) vector(end_time)]);
end
```

```
function [auc_ans] = auc2(data, start_time,interval)
%data = circ_cyt_conc_tc;
%start_time = 50;
%interval = 400;
col = size(data,2);
end_time = interval + start_time;
auc_ans = nan(size(data,2), 1);
for i = 1:col
    data2 = data(:,i);
    %auc_ans(i) = trapz(data2(start_time:end_time))-trapz([start_time end_time], [data2(start_time)
data2(end_time)]);
    auc_ans(i) = trapz(data2(start_time:end_time))-trapz([start_time end_time],[data2(start_time) data2(start_time)]);
end
auc_ans = auc_ans(:);
```

FOR CALCULATING SIMULATION AREA UNDER THE CURVE

```
function results = compare_curves(cyt_shape1, cyt_shape2, nuc_shape1, nuc_shape2)
load('G:\IYENGAR LAB FILES\2017\Feb\2-27-2017\spatial_simulation_reuslts.mat');
time = 1:501;
nuc_label1={'NPC=0.05', 'NPC=0.2', 'NPC=0.37', 'NPC=0.5', 'NPC=0.7', 'NPC=0.9', 'NPC=1.0'};
nuc_label2={'NPC=0.01', 'NPC=0.04', 'NPC=0.05', 'NPC=0.07', 'NPC=0.09', 'NPC=0.1', 'NPC=0.2',...
    'NPC=0.3', 'NPC=0.37', 'NPC=0.5', 'NPC=0.7', 'NPC=0.8', 'NPC=0.9', 'NPC=1.0'};
start_time = 101;
end_time = 400;
%AUC_cyt_circle = nan(((size(cyt_shape1,2))*(size(cyt_shape2,2))),1);
AUC_cyt_circle = [];
AUC_cyt_oval = [];
AUC_nuc_circle = [];
AUC_nuc_oval = [];
AUC_cyt_diff = [];
AUC_nuc_diff = [];
peak_diff_cyt = [];
peak_diff_nuc = [];
results = [AUC_cyt_circle, AUC_cyt_oval, AUC_nuc_circle, AUC_nuc_oval, AUC_cyt_diff, AUC_nuc_diff, peak_diff_cyt, peak_diff_nuc];
%AUC_
%AUC_cyt_oval = [];
```

```
n = 0;
num_plots = 12;
figure
hold on

for nuc1 = 1:(size(cyt_shape1, 2))
    for nuc2 = 1:(size(cyt_shape2, 2))
        n = n+1;
        if n <= num_plots

            % Cytosolic calcium time course subplot
            subplot(3,4,n)
            y1 = cyt_shape1(:,nuc1);
            y2 = cyt_shape2(:,nuc2);
            plot(time, y1, 'r', time, y2, 'b')
            %legend(['circ' nuc_label(nuc1)], ['oval' nuc_label(nuc2)]);
            leg1 = char(strcat('circ-', nuc_label1(nuc1)));
            leg2 = char(strcat('oval-', nuc_label2(nuc2)));
            legend(leg1, leg2)
            xlim([101 400]);
            xlabel('time (s)') ;
            ylabel('Ca (uM)');
            title(['circ', num2str(nuc1), 'vs', 'oval', num2str(nuc2)])

            % Calculate the AUC parameters
            AUC_cyt_1 = trapz(y1(start_time:end_time))-trapz([start_time end_time], [y1(start_time) y1(end_time)]);
            AUC_cyt_2 = trapz(y2(start_time:end_time))-trapz([start_time end_time], [y2(start_time) y2(end_time)]);
            AUC_delta  = abs((AUC_cyt_1 - AUC_cyt_2)/(AUC_cyt_1))*100;
            AUC_cyt_circle = [AUC_cyt_circle; AUC_cyt_1];
            AUC_cyt_oval = [AUC_cyt_oval; AUC_cyt_2];
            AUC_cyt_diff = [AUC_cyt_diff; AUC_delta];

            %Calculate peak differences
            peak1 = time(y1 == max(y1));
            peak2 = time(y2 == max(y2));
            peak_diff = peak1 - peak2;
            peak_diff_cyt = [peak_diff_cyt; peak_diff];
            title(['circ', num2str(nuc1), 'vs', ' oval', num2str(nuc2), 'peakd', num2str(peak_diff)])


            n = n+1;
            %Cyt AUC subplot
            subplot(3,4,n)
            bar([AUC_cyt_1 AUC_cyt_2])
            title(num2str(AUC_delta))
            set(gca, 'xticklabel', {'circle', 'oval'})

            n = n+1;
            % Nuclear calcium time course subplot
            subplot(3,4,n)
            ynuc1 = nuc_shape1(:,nuc1);
            ynuc2 = nuc_shape2(:,nuc2);
            plot(time, ynuc1, 'r', time, ynuc2, 'b')
            legx1 = char(strcat('circ-nuc', nuc_label1(nuc1)));
            legx2 = char(strcat('oval-nuc', nuc_label2(nuc2)));
            legend(legx1, legx2)
            xlim([101 400]);
            xlabel('time (s)') ;
            ylabel('Ca (uM)');

            %Calculate AUC parameters
            AUC_nuc_1 = trapz(ynuc1(start_time:end_time))-trapz([start_time end_time], [ynuc1(start_time) ynuc1(end_time)]);
            AUC_nuc_2 = trapz(ynuc2(start_time:end_time))-trapz([start_time end_time], [ynuc2(start_time) ynuc2(end_time)]);
            AUC_delta = abs((AUC_nuc_1 - AUC_nuc_2)/(AUC_nuc_1))*100;
            AUC_nuc_circle = [AUC_nuc_circle; AUC_nuc_1];
            AUC_nuc_oval = [AUC_nuc_oval; AUC_nuc_2];
            AUC_nuc_diff = [AUC_nuc_diff;AUC_delta];

            %Calculate peak differences
            peak_nuc1 = time(ynuc1 == max(ynuc1));
```

```
    peak_nuc2 = time(ynuc2 == max(ynuc2));
    peak_nuc_diff = peak_nuc1 - peak_nuc2;
    peak_diff_nuc = [peak_diff_cyt; peak_nuc_diff];
    title(num2str(peak_nuc_diff))

    n = n+1;
    %Nuc AUC subplot
    subplot(3, 4, n)
    bar([AUC_nuc_1 AUC_nuc_2]);
    set(gca, 'xticklabel', {'circle', 'oval'})
    title(num2str(AUC_delta));

elseif n == num_plots+1;
    figure
    hold on

    %Cytosolic calcium timecourse
    subplot(3,4,1)
    y1 = cyt_shape1(:,nuc1);
    y2 = cyt_shape2(:,nuc2);
    plot(time, y1, 'r', time, y2, 'b')
    %legend(['circ' nuc_label(nuc1)], ['oval' nuc_label(nuc2)]);
    leg1 = char(strcat('circ-', nuc_label1(nuc1)));
    leg2 = char(strcat('oval-', nuc_label2(nuc2)));
    legend(leg1, leg2)
    xlim([101 250]);
    xlabel('time (s)');
    ylabel('Ca (uM)');

    %AUC parameters
    AUC_cyt_1 = trapz(y1(start_time:end_time))-trapz([start_time end_time], [y1(start_time) y1(end_time)]);
    AUC_cyt_2 = trapz(y2(start_time:end_time))-trapz([start_time end_time], [y2(start_time) y2(end_time)]);
    AUC_delta  = abs((AUC_cyt_1 - AUC_cyt_2)/(AUC_cyt_1))*100;
    AUC_cyt_circle = [AUC_cyt_circle; AUC_cyt_1];
    AUC_cyt_oval = [AUC_cyt_oval; AUC_cyt_2];
    AUC_cyt_diff = [AUC_cyt_diff; AUC_delta];


    %Calculate peak differences
    peak1 = time(y1 == max(y1));
    peak2 = time(y2 == max(y2));
    peak_diff = peak1 - peak2;
    peak_diff_cyt = [peak_diff_cyt; peak_diff];
    title(['circ', num2str(nuc1), 'vs', ' oval', num2str(nuc2), 'peakd', num2str(peak_diff)])
    %title(['circ', num2str(nuc1), 'vs', 'oval', num2str(nuc2)])

    %Calculate nuclear calcium time course
    subplot(3,4,2)
    bar([AUC_cyt_1 AUC_cyt_2])
    set(gca, 'xticklabel', {'circle', 'oval'})
    title(num2str(AUC_delta))
    subplot(3,4,3)
    ynuc1 = nuc_shape1(:,nuc1);
    ynuc2 = nuc_shape2(:,nuc2);
    plot(time, ynuc1, 'r', time, ynuc2, 'b')
    legx1 = char(strcat('circ-nuc', nuc_label1(nuc1)));
    legx2 = char(strcat('oval-nuc', nuc_label2(nuc2)));
    legend(legx1, legx2)
    xlim([101 400]);
    xlabel('time (s)');
    ylabel('Ca (uM)');

    %Calculate peak differences
    peak_nuc1 = time(ynuc1 == max(ynuc1));
    peak_nuc2 = time(ynuc2 == max(ynuc2));
    peak_nuc_diff = peak_nuc1 - peak_nuc2;
    peak_diff_nuc = [peak_diff_cyt; peak_nuc_diff];
    title(num2str(peak_nuc_diff))

    %Nuc AUC parameters
```

```
AUC_nuc_1 = trapz(ynuc1(start_time:end_time))-trapz([start_time end_time], [ynuc1(start_time) ynuc1(end_time)]);
AUC_nuc_2 = trapz(ynuc2(start_time:end_time))-trapz([start_time end_time], [ynuc2(start_time) ynuc2(end_time)]);
AUC_delta = abs((AUC_nuc_1 - AUC_nuc_2)/(AUC_nuc_1))*100;
AUC_nuc_circle = [AUC_nuc_circle; AUC_nuc_1];
AUC_nuc_oval = [AUC_nuc_oval; AUC_nuc_2];
AUC_nuc_diff = [AUC_nuc_diff;AUC_delta];

subplot(3,4,4)
%NUC AUC plot
bar([AUC_nuc_1 AUC_nuc_2])
set(gca, 'xticklabel', {'circle', 'oval'})
title(num2str(AUC_delta))
n = 4;
    end
  end
end
```

REFERENCES

1. Bird, R. B., Stewart, W. E. & Lightfoot, E. N. *Transport Phenomena*. vol. 7 (Wiley, 2006).
2. Kreyszig, E. *Introduction to differential geometry and Riemannian geometry*. (University of Toronto Press, 1969).
3. Bressloff, P. C. & Newby, J. M. Stochastic models of intracellular transport. *Rev. Mod. Phys.* **85**, 135–196 (2013).
4. Bressloff, P. C. & Earnshaw, B. A. Diffusion-trapping model of receptor trafficking in dendrites. *Phys. Rev. E Stat. Nonlin. Soft Matter Phys.* **75**, 041915 (2007).
5. Cooling, M., Hunter, P. & Crampin, E. J. Modeling Hypertrophic IP3 Transients in the Cardiac Myocyte. *Biophys. J.* **93**, 3421–3433 (2007).
6. Vollmer, J., Menshykau, D. & Iber, D. Simulating organogenesis in COMSOL: Cell-based signaling models. *arXiv [q-bio.QM]* (2013).
7. Loew, L. M. & Schaff, J. C. The Virtual Cell: A software environment for computational cell biology. *Trends in Biotechnology* vol. 19 401–406 (2001).

8. Eungdamrong, N. J. & Iyengar, R. Compartment-specific feedback loop and regulated trafficking can result in sustained activation of Ras at the Golgi. *Biophys. J.* **92**, 808–815 (2007).

9. Bhalla, U. S. & Iyengar, R. Emergent properties of networks of biological signaling pathways. *Science* **283**, 381–387 (1999).

10. Li, Y. X. & Rinzel, J. Equations for InsP3 receptor-mediated [Ca2+]i oscillations derived from a detailed kinetic model: a Hodgkin-Huxley like formalism. *J. Theor. Biol.* **166**, 461–473 (1994).

11. Fink, C. C. *et al.* An image-based model of calcium waves in differentiated neuroblastoma cells. *Biophys. J.* **79**, 163–183 (2000).

12. De Young, G. W. & Keizer, J. A single-pool inositol 1,4,5-trisphosphate-receptor-based model for agonist-stimulated oscillations in Ca2+ concentration. *Proc. Natl. Acad. Sci. U. S. A.* **89**, 9895–9899 (1992).

13. Lukas, T. J. A signal transduction pathway model prototype I: From agonist to cellular endpoint. *Biophys. J.* **87**, 1406–1416 (2004).

14. Fink, C. C. *et al.* Morphological control of inositol-1,4,5-trisphosphate-dependent signals. *J. Cell Biol.* **147**, 929–936 (1999).

15. Kapustina, M. *et al.* Mechanical and biochemical modeling of cortical oscillations in spreading cells. *Biophys. J.* **94**, 4605–4620 (2008).

16. Quintana, A. R., Wang, D., Forbes, J. E. & Waxham, M. N. Kinetics of calmodulin binding to calcineurin. *Biochem. Biophys. Res. Commun.* **334**, 674–680 (2005).

17. Lee, M. & Park, J. Regulation of NFAT activation: a potential therapeutic target for immunosuppression. *Mol. Cells* **22**, 1–7 (2006).

18. Khalilimeybodi, A., Daneshmehr, A. & Sharif Kashani, B. Ca2+-dependent calcineurin/NFAT signaling in β-adrenergic-induced cardiac hypertrophy. *Gen. Physiol. Biophys.* **37**, 41–56 (2018).

19. Rangamani, P., Levy, M. G., Khan, S. & Oster, G. Paradoxical signaling regulates structural plasticity in dendritic spines. *Proceedings of the National Academy of Sciences* **113**, E5298-307 (2016).

20. Knot, H. J. & Nelson, M. T. Regulation of arterial diameter and wall [Ca2+] in cerebral arteries of rat by membrane potential and intravascular pressure. *J. Physiol.* **508 (Pt 1)**, 199–209 (1998).

21. Dickinson, G. D., Ellefsen, K. L., Dawson, S. P., Pearson, J. E. & Parker, I. Hindered cytoplasmic diffusion of inositol trisphosphate restricts its cellular range of action. *Sci. Signal.* **9**, ra108 (2016).

22. Bers, D. M. Cardiac excitation-contraction coupling. *Nature* **415**, 198–205 (2002).

23. Xu, C., Watras, J. & Loew, L. M. Kinetic analysis of receptor-activated phosphoinositide turnover. *J. Cell Biol.* **161**, 779–791 (2003).

24. Golebiewska, U. *et al.* Membrane-bound basic peptides sequester multivalent (PIP2), but not monovalent (PS), acidic lipids. *Biophys. J.* **91**, 588–599 (2006).

25. Sanabria, H., Digman, M. A., Gratton, E. & Waxham, M. N. Spatial diffusivity and availability of intracellular calmodulin. *Biophys. J.* **95**, 6002–6015 (2008).

26. Hong, F. *et al.* Diffusion of myosin light chain kinase on actin: A mechanism to enhance myosin phosphorylation rates in smooth muscle. *J. Cell Biol.* **211**, 2111OIA229 (2015).

27. Gorski, S. A., Dundr, M. & Misteli, T. The road much traveled: trafficking in the cell nucleus. *Curr. Opin. Cell Biol.* **18**, 284–290 (2006).

28. Lee, Christopher T., et al. "3D mesh processing using GAMer 2 to enable reaction-diffusion simulations in realistic cellular geometries." PLoS computational biology 16.4 (2020): e1007756.
